# Dynamic Changes in Ezh2 Gene Occupancy Underlie Its Involvement in Neural Stem Cell Self-Renewal and Differentiation towards Oligodendrocytes

**DOI:** 10.1371/journal.pone.0040399

**Published:** 2012-07-12

**Authors:** Falak Sher, Erik Boddeke, Marta Olah, Sjef Copray

**Affiliations:** Department of Neuroscience, University Medical Center Groningen, University of Groningen, Groningen, The Netherlands; Radboud University Nijmegen, The Netherlands

## Abstract

**Background:**

The polycomb group protein Ezh2 is an epigenetic repressor of transcription originally found to prevent untimely differentiation of pluripotent embryonic stem cells. We previously demonstrated that Ezh2 is also expressed in multipotent neural stem cells (NSCs). We showed that Ezh2 expression is downregulated during NSC differentiation into astrocytes or neurons. However, high levels of Ezh2 remained present in differentiating oligodendrocytes until myelinating. This study aimed to elucidate the target genes of Ezh2 in NSCs and in premyelinating oligodendrocytes (pOLs).

**Methodology/Principal Findings:**

We performed chromatin immunoprecipitation followed by high-throughput sequencing to detect the target genes of Ezh2 in NSCs and pOLs. We found 1532 target genes of Ezh2 in NSCs. During NSC differentiation, the occupancy of these genes by Ezh2 was alleviated. However, when the NSCs differentiated into oligodendrocytes, 393 of these genes remained targets of Ezh2. Analysis of the target genes indicated that the repressive activity of Ezh2 in NSCs concerns genes involved in stem cell maintenance, in cell cycle control and in preventing neural differentiation. Among the genes in pOLs that were still repressed by Ezh2 were most prominently those associated with neuronal and astrocytic committed cell lineages. Suppression of Ezh2 activity in NSCs caused loss of stem cell characteristics, blocked their proliferation and ultimately induced apoptosis. Suppression of Ezh2 activity in pOLs resulted in derangement of the oligodendrocytic phenotype, due to re-expression of neuronal and astrocytic genes, and ultimately in apoptosis.

**Conclusions/Significance:**

Our data indicate that the epigenetic repressor Ezh2 in NSCs is crucial for proliferative activity and maintenance of neural stemness. During differentiation towards oligodendrocytes, Ezh2 repression continues particularly to suppress other neural fate choices. Ezh2 is completely downregulated during differentiation towards neurons and astrocytes allowing transcription of these differentiation programs. The specific fate choice towards astrocytes or neurons is apparently controlled by epigenetic regulators other than Ezh2.

## Introduction

Multipotent neural stem cells (NSCs) give rise to neurons, astrocytes and oligodendrocytes. Insight in the molecular regulatory mechanisms underlying NSC self-renewal and differentiation into each of these cell types is of fundamental importance for understanding proper brain development, for explaining brain tumor formation and for application of NSCs in regenerative therapies for various neurodegenerative disorders.

The differentiation of NSCs into a specific neural cell type is ultimately determined by an interplay between extrinsic and intrinsic factors. Several signaling pathways are intricately involved in triggering a distinct set of transcription factors, which in turn set off the transcription of genes that determine a specific neural cell type. In case of neuronal differentiation, it is essential that, besides active transcription of neuronal genes, the transcription of genes encoding for a glial fate is suppressed and in case of glial cell differentiation, vice versa [1]–[4]. It has become clear that epigenetic programming is implicated in specifying the fate of NSCs, in particular, in the silencing of genes that encode for alternative cell fates [5]. Polycomb group (PcG) proteins have emerged as central players in such repressive epigenetic programming events.

PcG proteins were originally identified in *Drosophila melanogaster* (*D. melanogaster*) as factors necessary to preserve cell-fate decisions throughout embryogenesis by silencing *Hox* genes in a body-segment-specific manner [6], [7]. PcG proteins are transcriptional repressors that function by modulating and altering higher-order chromatin structure at the site of their target genes [8], [9]. Hundreds of genes are silenced by polycomb proteins, including dozens of genes that encode crucial developmental regulators in organisms ranging from plants to human [10].

PcG proteins are structurally and functionally diverse and form large multimeric complexes of two general types: Polycomb repressive complex-1 (PRC1) and -2 (PRC2) [11]. Biochemical purification of PRC1 from mammalian cells has revealed the presence of a number of subunits including BMI1/MEL18, RING1A/RING1B/RNF2, hPC 1–3, hPH1–3, and YY1, among others [12]. PRC2 contains Eed, Suz12, and the methyltransferase Ezh2 that catalyzes histone H3 lysine 27 trimethylation (H3K27me3) [13], [14]. Both Suz12 and Eed are required for complex stability and for the methyltransferase activity of the Ezh2 [15]. A common contention of the current models is that PRC2 initiates transcriptional repression, whereas PRC1 maintains the repressive state. Ezh2 mediated H3K27 methylation is required for the function of PRC1 and ultimate target gene silencing [16]. Ezh2-mediated transcriptional silencing depends upon its evolutionarily conserved catalytic SET domain, which imparts histone methyltransferase activity to the complex [13]. Functional mutations in the components of PRCs eradicate the aptitude of embryonic stem (ES) cells to be pluripotent [17]. Indeed, genome-wide chromatin immunoprecipitation (ChIP) studies and other approaches have greatly expanded our knowledge of the PRCs and it has become evident that transcription factors and signaling components with key roles in cell fate decisions are the most frequent PRC2 targets [18].

Ezh2, the pivotal catalytic component of PRC2, has received considerable attention during recent years. In accordance with the suggested function of PRC2 in maintaining ES pluripotency and ES self-renewal, Ezh2 is highly expressed in ES cells and down regulated during differentiation towards somatic cells [19]. Using a conditional knockout strategy, Ezh2 was also shown to be required for H3K27me3 formation in pluripotent epiblast cells that ultimately give rise to derivatives of the three germ layers [20]. In addition to its role in early embryonic development, Ezh2 has also been implicated in regulating the maintenance and self-renewal of multipotent tissue-specific stem cells, such as muscle cell stem cells [21], hematopoietic stem cells [22], and epidermal stem cells [23] and by that controlling proceeding into differentiation.

Previously, we have demonstrated the expression of Ezh2 in NSCs [24]. Its expression in undifferentiated NSCs and subsequent downregulation during the differentiation into neurons or astrocytes, suggested a role of Ezh2 in the maintenance and self-renewal of these multipotent stem cells. Indeed, we have shown that forced expression of Ezh2 in astrocytes induced their dedifferentiation into NSC-like cells [25]. Moreover, recent papers have elucidated the involvement of Ezh2 and the PRC2 complex in the control of neuronal and astrocytic differentiation during neocortical development [26], [27]. Surprisingly, Ezh2 remained highly expressed in NSCs that differentiated toward the oligodendrocytic cell lineage. Ezh2 expression was present in the early proliferating oligodendrocyte precursor cells (OPCs) up to the stage of premyelinating oligodendrocytes (pOLs). In mature, myelinating oligodendrocytes Ezh2 expression was no longer detectable [24]. The differentiation stages of OPCs are most strikingly characterized by derepression, i.e. silencing of repressors of oligodendrocyte cell lineage genes and myelination genes [28]. In view of its known repressor functions, Ezh2 may play a role in the repressive epigenetic mechanisms during oligodendrocyte differentiation and maturation.

In order to further investigate the role of Ezh2 in undifferentiated NSCs and in the differentiation of oligodendrocytes, we have used chromatin immunoprecipitation and massive parallel sequencing (Chip-Seq: high-throughput sequencing) to map the Ezh2 target genes in NSCs and those in pOLs. In addition, we aimed to present further evidence for the relevance of Ezh2 expression in pOLs *in vivo*, during de- and remyelination in the cuprizone mouse model.

## Methods

All animal work was designed and carried out in accordance with the National Institutes of Health Guide for the Care and Use of Laboratory Animals and regulations of the local Experimental Animal Committee at the UMCG, University of Groningen, Groningen, The Netherlands.

### Cell Culture

#### Primary mouse neural stem cell culture

Neural stem cells (NSCs) were isolated from the telencephalon of C57/BL6 mouse embryos at embryonic day 14 (E14). Cells were cultured in T25 (Nunc, Roskilde) tissue culture flasks containing proliferation medium, consisting of Neurobasal medium supplemented with 2% B27, human recombinant epidermal growth factor (EGF; 20 ng/ml), basic fibroblast growth factor (bFGF; 20 ng/ml), 1% GlutaMAX (Invitrogen) Primocin (100 micrograms/ml; Lonza), and heparin (5 micrograms/ml; Sigma-Aldrich) in a humidified 5% CO2/95% air incubator at 37°C. Within 3–5 days, the cells grew as free-floating neurospheres. NSC differentiation was induced by withdrawing growth factors (EGF & bFGF) and heparin from the proliferation medium.

#### Primary murine oligodendrocyte cultures

Mixed glial cultures were established from the brains of postnatal C57/BL6 pups in T75 cm^2^ tissue culture flasks containing Dulbecco’s modified Eagle’s medium (DMEM) with 10% FCS. After 10–14 days *in vitro*, the mixed glial cultures were placed on an orbital shaker (150 rpm) at 37°C for one to two hours, after which the detached microglia were removed from the supernatant. To obtain oligodendrocyte precursor cells (OPCs), cultures were placed on a shaker again (240 rpm) overnight at 37°C. On the following day, the supernatants were centrifuged and the pellets were resuspended in culture medium and plated on uncoated culture dishes for 30 minutes at 37°C. Microglia and astrocytes attached to the bottom of the culture dish, whereas oligodendrocyte precursor cells (OPCs) remained floating. In consecutive panning steps the floating cells were transferred from one culture dish to another until a pure (90–95%) OPC culture was obtained. The primary OPCs were cultured in standard SATO medium. During the first 2 days of culture, SATO medium was supplemented with Sonic hedgehog (100 ng/ml; R&D Systems) platelet-derived growth factor (PDGF)-α (10 ng/ml; R&D Systems) and basic fibroblast growth factor (bFGF; 20 ng/ml). During subsequent culturing, the OPCs differentiated and maturated along standard stages. At the pOLs stage (RIP-positive) the cells were used for chromatin immunoprecipitation.

### Chromatin Immunoprecipitation (ChIP)

We followed Upstate’s method for chromatin immunoprecipitation with minor adaptations. Mouse primary NSCs and pOLs were cross-linked with 1% formaldehyde for 10 minutes, lysed in lysis buffer (5 mM PIPES, 85 mM KCl, 0.5% NP-40; Sigma), followed by nuclear lysis buffer (50 mM TRIS, 10 mM EDTA, 1% SDS). Chromatin was fragmented (500–700 bp) using a sonicator (Sonics Vibracell). Fragmented chromatin was precleared with protein A agarose/salmon sperm DNA (Millipore). Precleared chromatin was incubated overnight at 4°C with 5 micrograms of anti-Ezh2 antibody (Diagenode pAB-039-100) and isotype control (IgG). The next day, protein A agarose/salmon sperm DNA was added and samples were rotated for 2 hours at 4°C. Resultant immune complexes were washed once with low salt buffer (150 mM NaCl, 0.1% SDS, 1% Triton X-100, 2 mM EDTA, 20 mM TRIS), once with high salt buffer (500 mM NaCl, 0.1% SDS, 1% Triton X-100, 2 mM EDTA, 20 mM TRIS), once with LiCl buffer (0.25 M LiCl,1% NP-40, 1% Na-deoxycholate, 1 mM EDTA, 10 mM TRIS) and twice with TE buffer. The immune complexes were eluted by adding twice 250 ul of freshly prepared elution buffer (0.1 M NaHCO_3_, 1% SDS). The DNA protein cross-links were reversed by heating with 200 mM sodium chloride, the proteins were removed by treatment with proteinase K and DNA was precipitated using the phenol/chloroform/isoamyl alcohol method.

Within each ChIP experiment, a negative control ChIP was performed using 5 micrograms of polyclonal rabbit anti-mouse immunoglobulin (IgG). To verify the ChIP conditions, a qPCR was performed on enriched DNA using promoter primers against Cdkn2a a known target of polycomb group proteins. The enrichment of immunoprecipitated DNA with anti-Ezh2 antibody versus negative control (IgG control) was calculated with following formula: FC  = 2∧(ddCT), FC.error  =  ln(2) * ddSD * FC. (FC  =  fold change, CT  =  cycle threshold, SD  =  standard deviation and d stands for delta). Multiple enriched samples were pooled and used for high-throughput sequencing (ChIP-Seq) on the Illumina Genome Analyzer IIx. The primer sets used for the ChIP experiments can be found in Table S2.

### ChIP-Sequencing (ChIP-Seq) and Data Analysis

The enriched and IgG control DNA was processed for analysis on the Illumina Genome Analyzer IIx using the Illumina ChIP-Seq sample preparation kit (IP-102-1001). Following the instructions on kit ‘A’, bases were added to the 3′ end of the DNA fragments and the fragments were ligated to Adapters. Subsequently, the Adapter-Modified DNA fragments were run on a 2% agarose gel to generate the DNA libraries based on fragments length (200±25 bp). Finally the Adapter-Modified DNA fragment libraries were enriched by PCR. After validating (quality control) the libraries by the Bioanalyzer, the sequencing reactions were performed with standard system components and reagents.

The sequence reads were analyzed using Galaxy [29], [30], a web based platform for computational and biomedical research. We obtained approximately 12.9 million reads from ChIP-Seq-NSCs, and 11.5 million from ChIP-Seq-pOLs samples. Before mapping, the quality control checks on raw sequence data coming from high throughput sequencing pipelines were performed using FastQC (http://www.bioinformatics.bbsrc.ac.uk/projects/fastqc/), a quality control tool for high throughput sequence data (complete FastQC reports on all data sets can be seen in Information S4, Information S5 and Information S6). Illumina 1.9 reads were converted to Sanger format by running the tool FASTQ Groomer. The reads with a quality score <10 were filtered out using the Filter FASTQ tool. The reads were also trimmed (16 bp from 3′ end) using FASTQ Trimmer tools. FASTQ data tools [31] were used from the Metaserver of Galaxy (for detail see Table S4 and FASTQC reports (pdf) for all three sequence files in Information S4, Information S5 and Information S6). After quality control, all three sequences (ChIP-Seq – NSCs, ChIP-Seq-pOLs and ChIP-Seq-IgG control) were aligned against the mouse mm9 genome using Bowtie for Illumina tool [32]. For the ChIP-Seq-NSCs, over 67% of the reads were mapped uniquely to the mm9 genome, whereas for the ChIP-Seq-pOLs this was about 30%. SAM files were converted to BAM format using SAM tools http://samtools.sourceforge.net [33] **.** BAM files can be directly viewed and analyzed at the UCSS genome browser. BAM files were converted to Wig and finally to BigWig format. They are available at the GEO database via link: http://www.ncbi.nlm.nih.gov/geo/query/acc.cgi?acc=GSE31655. To identify peaks we used the Model-based Analysis of ChIP-Seq (MACS) tool (http://cistrome.org/ap/root) [34], [35]. MACS detected 1571 regions in ChIP-Seq-NSCs and 607 regions in ChIP-Seq-pOLs as peak regions by using ChIP-Seq-IgG as a control. For the gene annotations, we used peak to gene script from the Galaxy/Cistrome. To avoid redundancies we performed visual inspection of each annotated gene peak detected by MACS on UCSC Genome Browser using BAM and BigWig tracks of ChIP-Seq-Ezh2 and ChIP-Seq-IgG (comparison of actual detected reads ChIP and IgG control). Some peaks identified by MACS occurred in a region where no substantial difference between the IgG control and ChIP could be observed. We concluded such peaks as false positives and they were ignored while preparing the final list (for details see Information S1 and Figure S5). Furthermore, we used Genomic Regions Enrichment of Annotations Tool (GREAT) to analyze the association of input genomic regions to the transcription start site (TSS) of all the genes putatively regulated by the genomic regions in NSCs and pOLs (http://great.stanford.edu/public/html/index.php).

Annotations and pathways analysis of the peaks were done either by manual search in the databases or by using online software including The Database for Annotation, Visualization and Integrated Discovery (DAVID) [36], [37] and Ingenuity Pathways Analysis (IPA) (http://www.ingenuity.com/).

### ShRNA Knockdown of Ezh2 in NSCs and pOLs

Ezh2 expression in NSCs and pOLs was silenced by using Ezh2 short hairpin RNA (Ezh2-shRNA) plasmid DNA construct targeting the coding region of the Ezh2 gene transcript (MISSION DNA NM 007971; Sigma-Aldrich). Five vectors (targeting different sequences in the coding region of Ezh2 transcript) for Ezh2 silencing were tested, of which two appeared to be similarly efficient in reducing Ezh2 expression (for details about these 2 Ezh2 shRNA, encoded sh-4 and sh-5, see Information S1, Figure S1B and C and Figure S4). The effects of Ezh2 silencing on NSCs and pOLs as described in the Results section were all obtained with one of the 2 efficient Ezh2shRNAs (sh-4); to exclude the possibility that they could be due to some off-target effects, we repeated some of the silencing experiments with the other efficient Ezh2 shRNA, sh-5, targeting other sequences in the coding region of the Ezh2 gene and depicted the results in Figure S1and Figure S2. All gene transfections were performed by using an electroporesis-based transfection protocol (Lonza previously known as Amaxa, http://www.lonzabio.com/cell-biology/transfection/) specifically designed for the transfection of mouse neural cells by Amaxa. 2–3 million cells were transfected with 3 micrograms of Ezh2-shRNA plasmid DNA construct. For control transfections, the same vector (pLKO.1-puro) (containing the sequence that does not target any mouse/human gene (nc-shRNA)) was used. After transfection, the cells were kept for 24 hours in a humidified 5% CO_2_-95% air incubator at 37°C in proliferation medium (NSCs) and Sato medium (pOLs). The transfection efficiency of this procedure amounted to 60–80% and resulted in the transient expression of the transfected gene lasting up to 10–12 days. Ezh2 repression after 24 hours of transfection was established at the RNA level (with quantitative reverse transcription polymerase chain reaction [qRT-PCR]) and at protein level (with Western blotting).

### Western Blot Analysis

Sodium dodecyl sulfate (SDS)-polyacrylamide gel electrophoresis and Western blot analysis were used to detect the expression of Ezh2 and other proteins in undifferentiated NSCs and OPCs. Cell lysates were made in nuclear lysis buffer/RIPA buffer, containing the Protease Inhibitor Cocktail from Sigma. Protein samples were made in Laemmli sample buffer and boiled for 5 minutes. Proteins were separated for 1.5 hours on a 7.5–15% SDS-polyacrylamide gel electrophoresis apparatus (Bio-Rad). Subsequently, proteins were transferred to nitrocellulose membranes by using semidry transfer buffer (25 mM Tris, 150 mM glycine, and 10% [v/v] methanol) and 3 mA/cm2 current for one to two hours. Nitrocellulose membranes were blocked with Odyssey Blocking Buffer (www.licor.com). Membranes were probed with antibodies overnight at 4°C. The antibodies against housekeeping proteins were used for normalization of the expression levels of the proteins of interest. Membranes were washed four times (10 minutes each) with TBS-T. Primary antibodies were detected using fluorescent secondary antibodies with incubation at room temperature for one hour. After four washes in TBS-T, protein signals were detected by using the Odyssey Infrared Imaging System. The intensities of the protein signals were quantified using ImageJ software (NIH Image, Bethesda, MD).

Statistical analysis of the results of three experiments was done using Student’s *t* test.

The specification of the primary and secondary antibodies we used can be found in Table S3.

### RT-PCR Analysis

Total RNA was isolated from NSCs and pOLs with or without Ezh2-shRNA treatment. Cells were lysed in guanidinium isothiocyanate/mercaptoethanol buffer and RNA was extracted with phenol-chloroform and precipitated using isopropanol. To degrade possible genomic DNA traces, DNase (Fermentas) was used. 1 microgram of total RNA was used for the reverse transcriptase (RT) reaction and 5 nanograms of copy DNA was used in the subsequent quantitative polymerase chain reaction (qPCR) amplification using appropriate primer pairs for each gene. The primer pair sequences we used for each gene can be found in Table S1 **.**

### Immunocytochemistry

Cell cultures were fixed with 4% paraformaldehyde (PFA) and immunostained using antibodies against specific proteins. For the immunostaining of brain tissue sections (fixed with 4% PFA) heat-induced antigen/epitope retrieval (HIER) (10 mM sodium citrate, 0.05% Tween 20, pH 6.0) was used. The specification of the primary and secondary antibodies we used for immunocytochemistry can be found in Table S3.

### Microscopic Analysis

Microscopic analysis was performed using a Zeiss (Axioskope 2) fluorescent microscope equipped with a Leica DFC300FX camera and the Leica Microsystems LAS program. The phase contrast images were made by Axiovert 40 CFl (Zeiss) microscope.

### Cuprizone Model for Demyelination

C57BL**/**6 mice were put on a diet of 0.2% (w/w) cuprizone (Sigma-Aldrich, Zwijndrecht, The Netherlands, http://www.sigmaaldrich.com), a copper chelator. This diet leads to selective oligodendrocyte death followed by demyelination of axons mainly in the corpus callosum [38] **.** Spontaneous remyelination can be seen as early as four days after withdrawal of cuprizone. The corpus callosum was dissected under a dissection microscope from control mice, from mice put on cuprizone diet for five weeks (demyelination state) and from mice put on normal diet again for two subsequent weeks (remyelination state). Tissue lysates were prepared with RIPA buffer. From mice in the same experimental groups, brains were prepared separately for immunohistochemistry.

## Results

### Genome Wide Identification of Target Genes of Ezh2 in NSCs and pOLs

**Figure 1:**
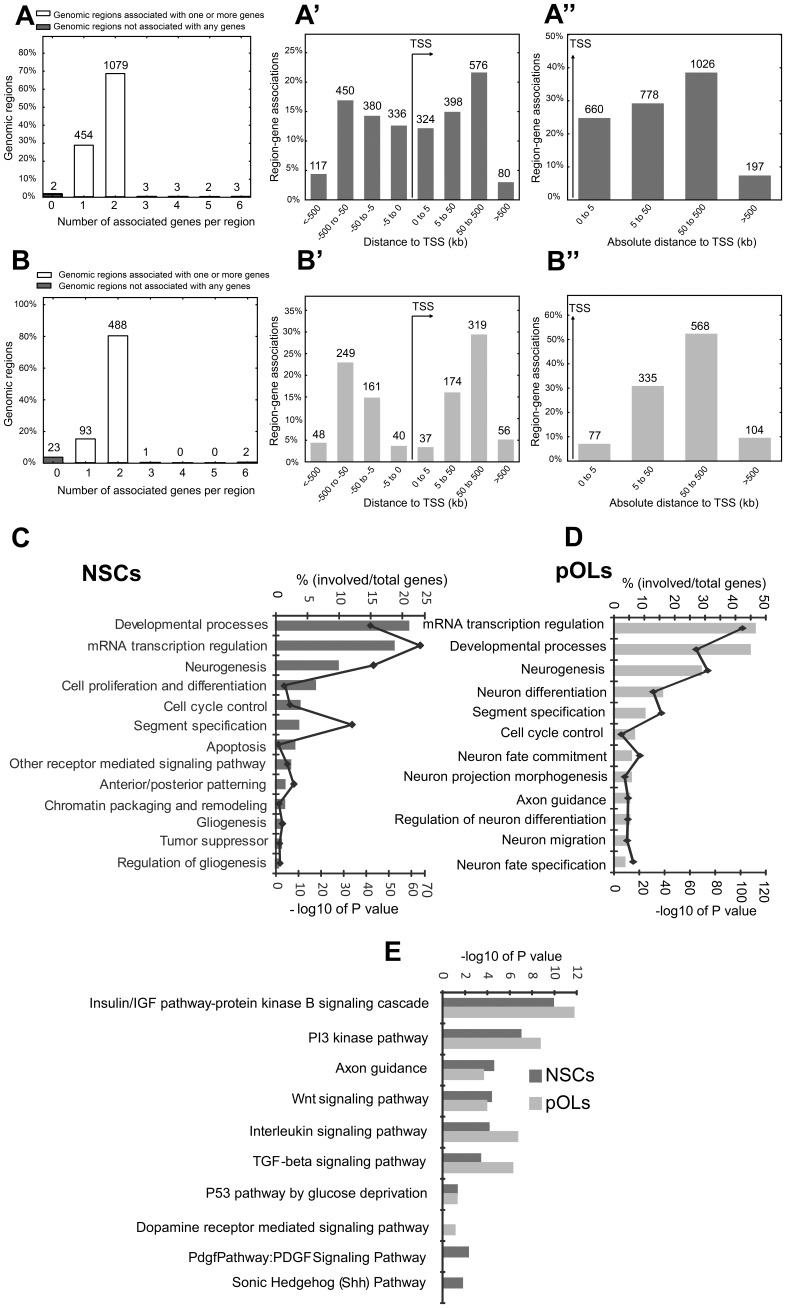
Target genes of Ezh2 in NSCs and pOLs. (**A**–**B”**) Association of input genomic regions with the transcription start site (TSS) of all the genes putatively regulated by the genomic regions in NSCs (**A**–**A”**) and pOLs (**B**–**B”**). (**A**, **B**) “Number of associated genes per region" graphs show how many genes in each genomic region is assigned as putatively regulating based on the association rule used. (Details of association rule see online: http://great.stanford.edu/public/html/help/index.php?title=Association_Rules). (**A’**, **B’**) Graphs show the distance between input regions and their putatively regulated genes (i.e. binned by orientation and distance to TSS). The distances are divided into four separate bins: 0 to 5 kb, 5 kb to 50 kb, 50 kb to 500 kb, and >500 kb. All associations precisely at 0 (i.e. on the TSS) are split evenly between the [−5 kb, 0] and [0,5 kb] bins. (**A”**, **B”**) Binned by absolute distance to TSS (only the distance to TSS is considered, not the orientation). Functional gene ontology (GO) analysis of Ezh2 target genes (DAVID online tool) in NSCs (**C**) and pOLs (**D**). The bar graphs represent the percentage of DAVID genes (since DAVID converts user input gene IDs to corresponding DAVID gene IDs) in the list associated with particular annotation term. The corresponding line graphs represent –log10 transformation of the modified Fisher Exact P-value. Fisher Exact is adopted to measure the gene-enrichment in annotation terms. The smaller the P-value, the more enriched a GO term is for genes of the given assay. (**E**) Comparison of canonical signaling pathways enriched in Ezh2 target genes in NSCs and pOLs.

To unravel the function of Ezh2 in NSCs and in premature oligodendrocytes (pOLs), we identified the target genes of Ezh2 in both cell types by chromatin immunoprecipitation (ChIP) using anti-Ezh2 antibodies, followed by ultra high-throughput massive parallel sequencing (ChIP-Seq). We used the Illumina^R^ sequencing technology for ultra high-throughput sequencing of enriched genomic DNA (IP with anti-Ezh2) and of background noise genomic DNA (IP with isotype control IgG) in both cell types. Using the metaserver of Galaxy, a web-based platform for genome analysis, we mapped the sequence files with the reference mouse genome (mm9). To identify peaks, we used the Model-based Analysis of ChIP-Seq (MACS) tool from the Galaxy/Cistrome metaserver. We extracted significant ChIP peaks after normalizing with background noise (IgG control). To obtain the annotations for genes we used the “peak2gene” script from the Galaxy/Cistrome. All annotated gene peaks detected by MACS in both cell types were visually inspected in the UCSC genome browser by loading BAM/BigWig tracks of ChIP-Seq-Ezh2 and ChIP-Seq-IgG control. The peaks detected by MACS that showed no considerable difference between ChIP-Seq-Ezh2 and ChIP-Seq-IgG signal (with respect to number of reads) at the UCSC genome browser, were rejected. Finally, we filtered 1532 annotated gene peaks in NSCs and 393 in pOLs respectively as targets of Ezh2. All of the 393 genes in pOLs were also target in the NSCs. The complete lists of target genes in both cell types are provided in information S2 and information S3. As an additional quality control we analyzed, using the Genomic Regions Enrichment of Annotations Tool (GREAT) [39] the association of input genomic regions (peaks) to the transcription start site (TSS) of all the genes putatively regulated by the given genomic regions in NSCs (Figure 1A–A”) and pOLs (Figure 1B–B”). The algorithm found that less than 5% of the input genomic regions from both cell types was not putatively associated with any gene (Figure 1A and 1B). In both NSCs and pOLs most of the input genomic regions were found to be associated with one or two genes (Figure 1A and 1B). In both cell types most of the genomic regions from our Chip-Seq study were annotated within 500 bp distance of the transcription start site (TSS) of the putatively regulated gene (Figure 1A’ and A”; Figure 1B’ and 1B”). Less than 10% of the input genomic regions annotated further away from the TSS than 500 bp. These findings are in line with an earlier report [40] in which genomic sites occupied by PRC2 were found to be within 1 kbp distance of the TSS.

The pattern of Ezh2 gene occupancy during the differentiation of NSCs suggests that Ezh2 keeps on repressing a number of genes when cells enter the oligodendrocytic cell lineage. As extensively described before [24], no Ezh2 expression (at mRNA or protein level) and so no Ezh2 target genes could be found and pulled down with ChIP in freshly differentiated neurons or astrocytes (data not shown).

In order to reveal general functional characteristics of the molecular programs involving Ezh2, we first conducted a gene ontology analysis on the target gene lists of NSCs and pOLs using the online tool Database for Annotation, Visualization and Integrated Discovery (DAVID) v6.7. (Figures 1C and D). In NSCs, we found that Ezh2 targeted genes mainly associated with gene ontology terms like Developmental processes, mRNA transcription regulation, Neurogenesis, Cell proliferation and differentiation and Cell cycle control (Figure 1C). In pOLs the major significantly enriched gene ontology terms were also Developmental processes, mRNA transcription regulation and Neurogenesis but besides these, terms that refer to neuronal specification and neuronal fate commitment were also significantly enriched (Figure 1D).

Further insight in the function of the Ezh2 target genes in NSCs and in pOLs was obtained using DAVID tools that visualize genes on BioCarta & KEGG pathways and allow identification of the top canonical signaling, disease and metabolic pathways represented by the genes that are significantly enriched in a given cell type. We observed 7 significantly enriched pathways both in NSCs and pOLs (Figure 1E). Two pathways, i.e. the PDGF- and Shh-signaling pathway, were only apparent in NSCs, whereas the dopamine receptor mediated signaling pathway was only enriched in Ezh2 targets in pOLs.

**Figure 2:**
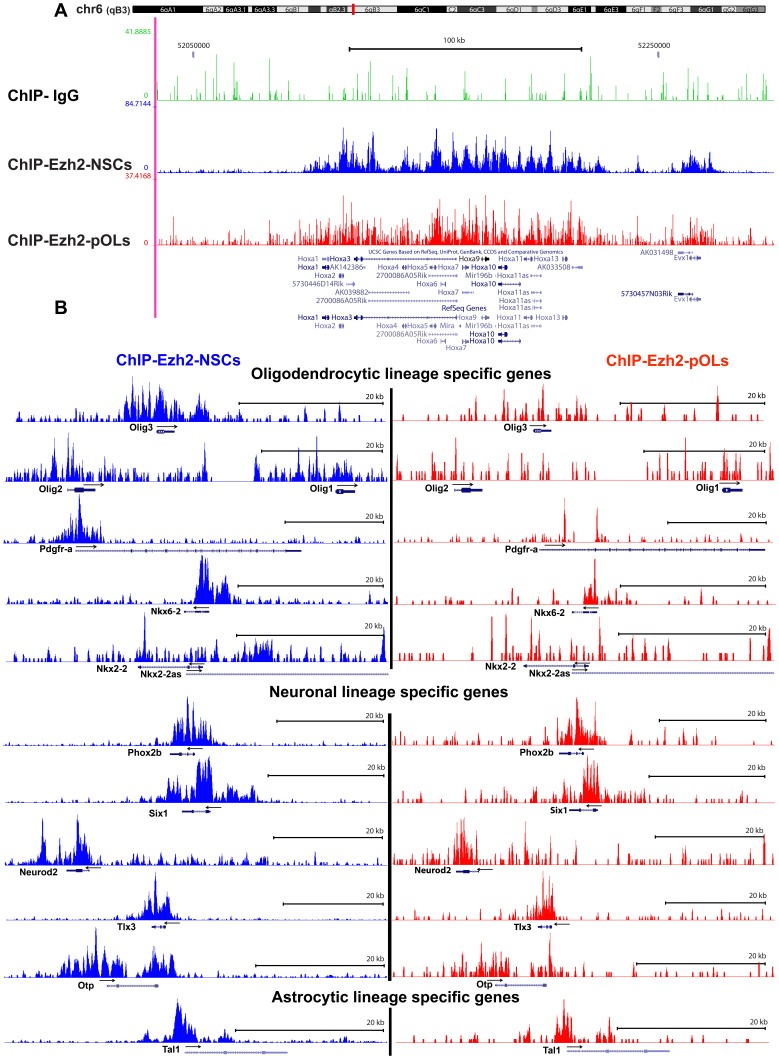
Target genes of Ezh2 in NSCs and pOLs: genome browser views. ChIP-Seq signals (peaks) are shown for anti-Ezh2 and IgG control antibodies after loading BigWig files on UCSC genome browser. (**A**) UCSC genome browser view of the known polycomb group protein target region, the Hox locus (located on chromosome 6 in the mouse), after loading three tracks: ChIP-Seq-IgG control, ChIP-Seq-NSCs and ChIP-Seq-pOLs. (**B**) ChIP-Seq–NSCs tracks show, neuronal, oligodendrocytic and astrocytic lineage determining genes as the targets of Ezh2. Upon differentiation of NSCs towards oligodendrocytes, as shown in the ChIP-Seq-pOLs tracks, Ezh2 releases the oligodendrocytic lineage determining genes, while still suppressing neuronal and astrocytic lineage genes.

Further analysis of the Ezh2 target genes coming up from the ChIP-Seq tracks in the UCSC genome browser view (Figure 2) revealed that in NSCs, genes are targeted which are involved in differentiation to each of the 3 neural cell types, i.e. oligodendrocytic lineage specific genes (e.g. *Olig2, Olig3, Pdgfra, Nkx2.2* and *6.2*), neuronal lineage specific genes (e.g. *NeuroD2*, *Tlx3* and *Phonx2b*) and astrocytic lineage specific genes (e.g. *Tal1*, also known as *Scl*). In pOLs, the oligodendrogenic lineage specific genes are no longer targeted by Ezh2, whereas the astrocytic and neuronal lineage specific genes still are. The Ezh2 targeted neuronal lineage genes not only include genes encoding general neuronal properties, but also genes involved in the differentiation of a more specific neuronal cell type. For instance, *Nurr1*, *Lmx1a/1b, En1/2, Otx2, Pitx3* and *Isl1* and *Isl2* are all Ezh2 target genes involved in dopaminergic neuron and motor neuron differentiation, respectively.

**Figure 3:**
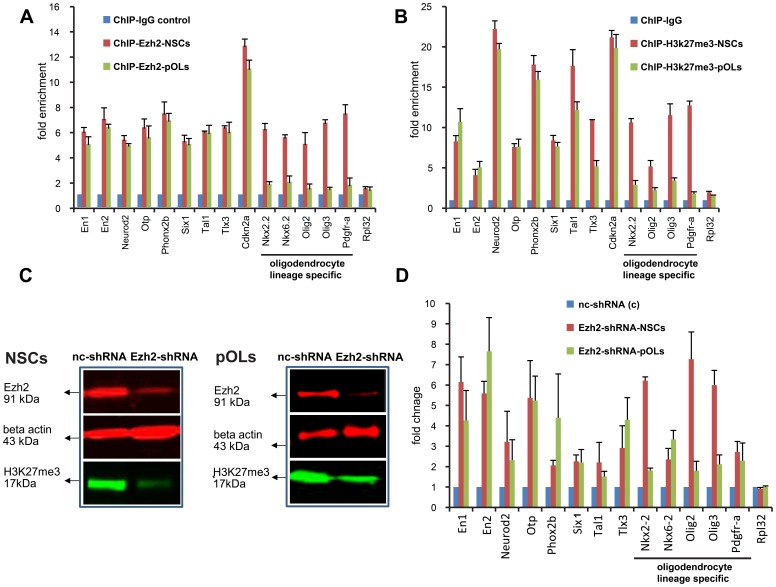
Chromatin immunoprecipitation (ChIP) using IgG isotype control, anti-Ezh2, and anti-H3K27me3 antibodies. (**A**) ChIP-Ezh2-quantitative PCR (qPCR) analysis. The graph shows quantitative PCR on DNA purified from ChIP-Ezh2, using promoter primers for genes associated with selected peaks from the ChIP-Seq data. In NSCs ChIP-Ezh2, there is high enrichment (Ezh2 occupancy) of neuronal, astrocytic and oligodendrocytic lineages determining genes while in pOLs ChIP-Ezh2 only neuronal and astrocytic lineage specific genes show enrichment (occupancy by Ezh2). (**B**) ChIP-H3K27me3-qPCR analysis. A similar pattern of gene enrichment can be observed in PCR analysis on DNA purified from ChIP-H3K27me3 (trimethylation of H3K27 is a functional mark of Ezh2): in NSCs, there is high enrichment (trimethylation of H3K27) of neuronal, astrocytic and oligodendrocytic lineages determining genes while in pOLs only neuronal and astrocytic lineage specific genes show high enrichment (higher level of trimethylation of H3K27). *Rpl32* (the gene encoding the 60S ribosomal protein L32, a non-target of Ezh2) was used as a negative control. *Acute Ezh2 reduction in NSCs and pOLs mediated by Ezh2-shRNA.* (**C**) Western blot shows the reduction in Ezh2 protein in comparison to control. Lysates were prepared after *in vitro* transfection with Ezh2-shRNA and non-coding shRNA (nc-shRNA) as control, in both NSCs and pOLs. Reduction in Ezh2 protein resulted in a decrease in the global level of H3K27 tri-methylation, an Ezh2-associated gene repression mark (17 kDa band). (**D**) Derepression of tested Ezh2 target genes after knocking down Ezh2 protein expression in NSCs and pOLs. The transcript levels were quantified by real-time PCR, normalized to GAPDH (housekeeping gene) and represented as a fold change between nc-shRNA (control) and Ezh2-shRNA in both NSCs and pOLs. Bars represent mean of three independent experiments ± S.D.

Validation of a selection of genes associated with peaks in the ChIP-Seq data using quantitative PCR analysis of DNA purified by Ezh2-ChIP (Figure 3A), confirms the evidence described above. In addition, we performed ChIP-qPCR analysis on trimethylated histone 3 lysine 27 (H3K27me3), since H3K27 trimethylation is the major functional mark of Ezh2. Quantitative PCR analysis of DNA purified from the H3K27me3-ChIP on the same selection of genes revealed a similar pattern as with Ezh2-ChIP: in NSCs, there is high enrichment (trimethylation of H3K27) of neuronal, astrocytic and oligodendrocytic lineages determining genes while in pOLs only neuronal and astrocytic lineage specific genes show high enrichment (higher level of trimethylation of H3K27) (Figure 3B).

Reduction of Ezh2 in NSCs and pOLs by silencing with Ezh2 specific shRNA decreases H3K27 tri-methylation (Figure 3C), enabling the transcription of the Ezh2 target genes and the expression of the proteins encoded by them. By PCR analysis of a selection of genes we demonstrated that, indeed, the Ezh2 target genes in NSCs and pOLs were re-expressed after Ezh2 silencing, resulting in a 3–10 fold increase in expression levels. However, for the target genes in pOLS that were no longer repressed by Ezh2, e.g. *Olig2*, this silencing did not lead to significant changes in comparison to the basal level of expression (Figure 3D).

### Acute Suppression of Ezh2 in NSCs

**Figure 4:**
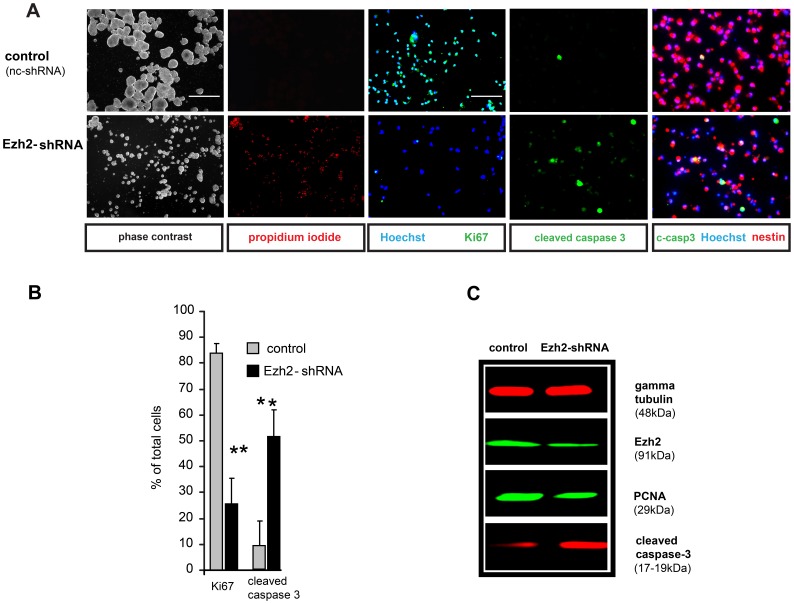
Acute reduction of Ezh2 induces apoptosis in NSCs. (A) Phase contrast images show that NSCs treated with Ezh2-shRNA cannot form normal neurospheres in proliferation medium. Immunostaining of dissociated neurospheres in proliferation medium shows a decrease in proliferation and increase in cell death after using Ezh2-shRNA compared to the control (nc-shRNA). (**B**) Quantification of proliferating (Ki67 positive) and apoptotic (cleaved caspase 3 positive) NSCs in control and NSC cultures treated with Ezh2-shRNA. (**C**) Western blot of protein samples prepared 48 hours after transfection shows a decrease in Ezh2 and PCNA (a proliferation marker) and an increase in cleaved caspase 3 bands in NSCs treated with Ezh2-shRNA. Bars represent mean of three independent experiments ± S.D.; Student-t-test was used to calculate significance. p<0.05 was considered significant. (Calibration bar in A represents 100 micrometers and 5 micrometers, which are valid for the phase contrast, propidium iodide and the Hoechst/Ki67, cleaved caspase 3, cleaved caspase 3/Hoechst/nestin photomicrographs, respectively (**A**); ** in (**B**) represents p<0.01).

**Figure 5:**
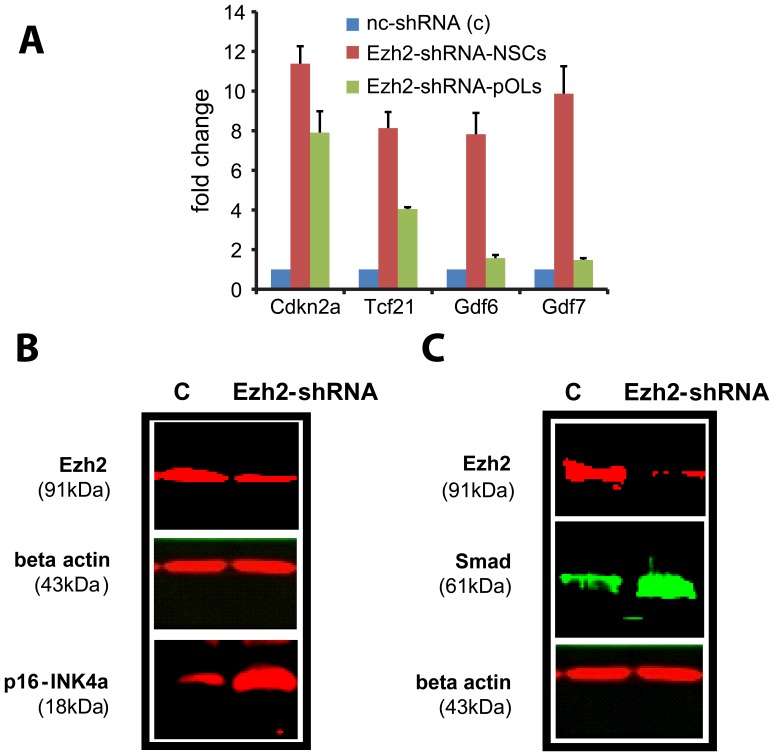
Cell cycle regulation and TGF-beta signaling in Ezh2-shRNA treated cells. (**A**) Real-time quantitative PCR analysis of total RNA extracted from nc-shRNA (control; C) and Ezh2-shRNA treated NSCs and pOLs shows increase in the transcript level of cell cycle arrest genes, however the transcript level of growth differentiation factors (Gdfs) belonging to TGF-beta superfamily increased only in Ezh2-shRNA treated NSCs (consistent with ChIP-seq data)**.** (**B**) Western blot confirms the upregulation of cell cycle arrest proteins (e.g. p16, a product of Cdkn2a) and the activation of TGF-beta signaling pathway (Smads) in Ezh2-shRNA treated NSCs as compared to the control (**C**).

To determine the significance of Ezh2 activity for NSCs maintenance and self-renewal, we followed Ezh2-shRNA transfected NSCs up to three days in culture as they developed into neurospheres during their clonal expansion. We observed dramatic changes in neurosphere morphology (Figure 4A): Ezh2 silenced neurospheres were smaller, more flattened and contained many dying cells in comparison to controls as revealed by propidium iodide staining. Immunostaining for the proliferation marker Ki67 revealed a strong reduction of proliferation activity (Figure 4A and 4B). Cleaved caspase 3 immunostaining confirmed the increase in apoptosis (Figure 4A and 4B). These effects of Ezh2-shRNA on NSCs were further confirmed by Western blot analysis using anti-PCNA (another proliferation marker) and anti-cleaved caspase 3 antibodies (Figure 4C). The results of these silencing experiments further substantiate the idea that Ezh2 mediated gene repression in NSCs is essential for maintenance (i.e. suppressing differentiation) and survival of NSCs and for controlling proliferation and cell cycle as has been shown before [24]. The results of acute Ezh2 silencing on NSC morphology and apoptosis as described above were obtained with one of the Ezh2shRNAs (sh-4); to exclude the possibility that they could be due to some off-target effects, we repeated these silencing experiments with the other efficient Ezh2 shRNA (sh-5, targeting other sequences in the coding region of the Ezh2 gene) and found similar results, depicted in Figure S1 and Figure S2. Our ChIP-Seq data analysis reveals a number of potential target genes of Ezh2 that are involved in cell cycle arrest, cell death and cellular compromise: *Cdkn2a, Tcf21* and *Six1*. Indeed, RT-qPCR analysis showed an 8 and 12 fold increase in transcript levels of *Tcf21* and *Cdkn2a* respectively (Figure 5A) after Ezh2 silencing, confirmed by Western Blotting using anti-p16-INK4a (a product of *Cdkn2a*) antibody (Figure 5B). In addition, the DAVID analysis revealed a significant involvement of Ezh2 target genes in NSCs in the TGF-beta signaling pathway. TGF-beta signaling plays an important role in various physiological and pathophysiological processes in the brain [41]. Various studies have demonstrated the involvement of TGF-beta signaling in suppressing NSC proliferation and self-renewal [41], [42]. *Gdf6* and *Gdf7*, subfamily members of the TGF-beta superfamily, appear to be Ezh2 target genes in NSCs. Suppressing Ezh2 activity should activate *Gdf6* and *Gdf7* expression, which should lead to activation of TGF-beta signaling and an upregulation of Smads, the downstream effectors of TGF-beta signaling. Indeed, with RT-qPCR analysis, we found a seven to ten fold upregulation in the transcripts of *Gdf6* and *Gdf7* in Ezh2-shRNA treated NSCs (Figure 5A). Moreover, Western blot analysis revealed an increase in the expression of Smads (Smad1, Smad2, Smad3, Smad5 and Smad8) in Ezh2-shRNA treated NSCs (Figure 5C).

The Ezh2 silencing data in NSCs point to a specific inhibition of cell cycle arrest genes (*Cdkn2a, Tcf21*) and genes involved in TGF beta signaling pathways by Ezh2, thus promoting cell proliferation and cell survival.

### Acute Suppression of Ezh2 in pOLs

**Figure 6:**
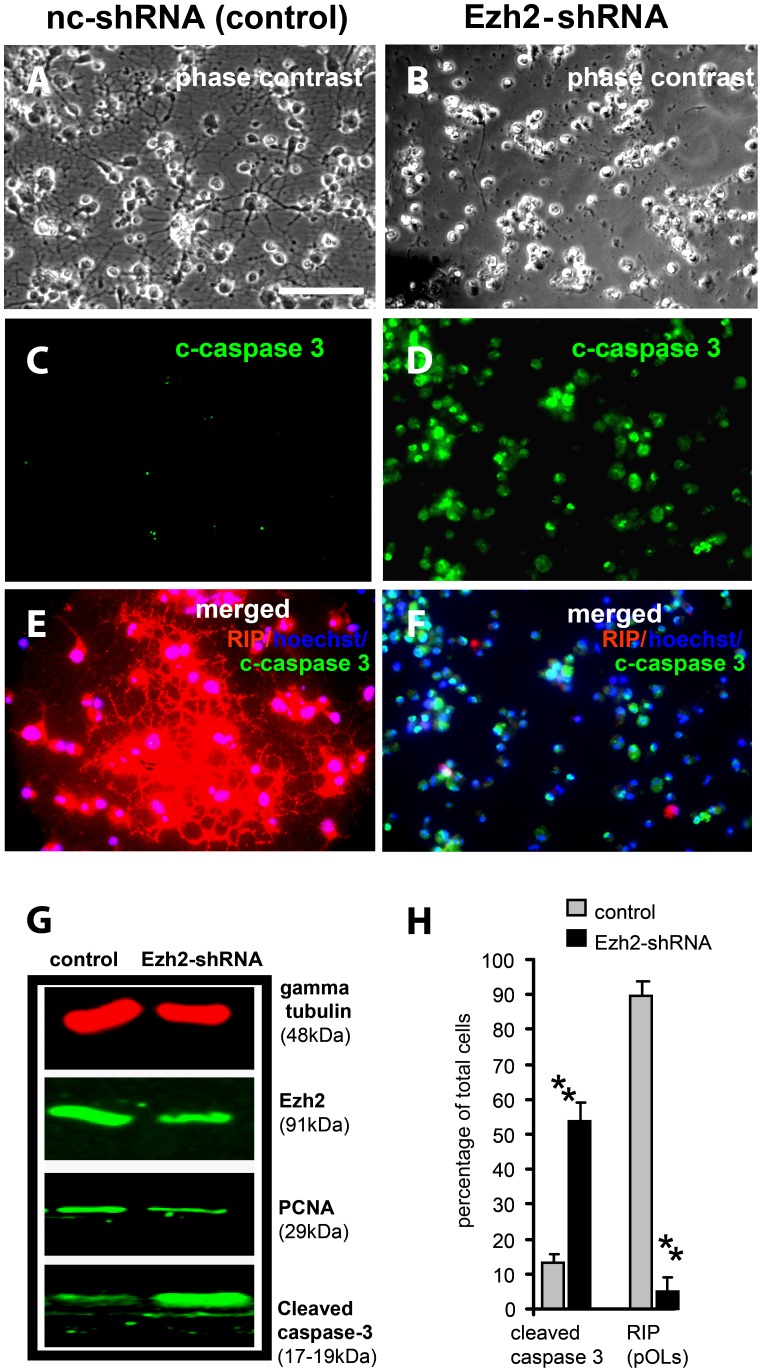
Acute suppression of Ezh2 induces apoptosis in pOLs. (**A**, **B**) Phase contrast images show that pOLs retract their projections when treated with Ezh2-shRNA. (**C**, **D**) Immunostaining for apoptosis marker, cleaved caspase 3 (green) shows increase in cell death in pOLs cultures treated with Ezh2-shRNA compared to control cultures (nc-shRNA). (**E**, **F**) Co-immunostaining using anti-cleaved caspase 3 and anti-RIP (an oligodendrocyte maker) shows restriction of cell extensions and increased cell death, in cultures treated with Ezh2-shRNA. (**G**) Western blot of protein samples prepared after 24 hours of transfection shows a decrease in Ezh2 and a strong increase in cleaved caspase 3 bands in pOLs treated with Ezh2-shRNA, compared to the cultures treated with nc-shRNA (control). PCNA (a proliferation marker) expression remained about the same in both conditions**.** (**H**) Quantification of apoptotic (cleaved caspase 3-positive) and differentiating (RIP-positive) cells in Ezh2-shRNA treated and control (nc-shRNA) pOLs. Bar graphs represent mean of three independent experiments ± S.D. (Calibration bar in (**A**) represents 100 micrometers, which is valid for all the photomicrographs (**A**–**F**); ** in (**H**) represents p<0.01).

Similar to NSCs, we investigated the significance of Ezh2-mediated gene repression for the maintenance and survival of pOLs. To that purpose we reduced Ezh2 expression by Ezh2-shRNA silencing in A2B5-positive pOLs enriched cultures (∼90%). pOLs treated with Ezh2-shRNA rapidly (within 24 hours) developed an abnormal morphology: they retracted their extensions, lost RIP immunostaining, and underwent apoptosis as indicated by high levels of cleaved caspase-3 (Figure 6). The results of Ezh2 silencing on pOL morphology and apoptosis as described above were obtained with one of the Ezh2shRNAs (sh-4); to exclude the possibility that they could be due to some off-target effects, we repeated the silencing experiments with the other efficient Ezh2 shRNA (sh-5, targeting other sequences in the coding region of the Ezh2 gene) and found similar results, depicted in Figure S1 and Figure S2. The ChIP-Seq data analysis revealed that pOLs have specific sets of genes responsible for neuronal and astrogenic cell lineage as Ezh2 target genes in common with NSCs. Indeed, most of these genes became upregulated after knocking down the expression of Ezh2 (see also Figure 3D) and their appearance in the differentiated pOLs must have resulted in prominent cell stress. Besides that, just like in NSCs, also in pOLs, cell cycle arrest and cell death genes like *Cdkn2a, Tcf21* are Ezh2 target genes, and their re-expression (see Figure 5A) after Ezh2 silencing may have caused apoptosis in a similar way as in the NSCs.

### Expression of Ezh2 in NSCs and pOLs in the Mouse Brain

**Figure 7:**
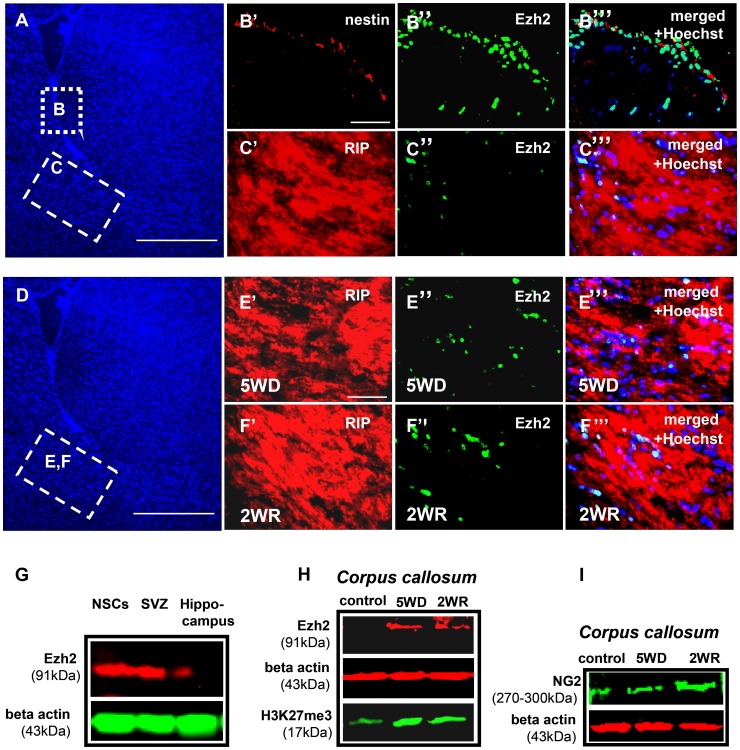
NSCs and pOLs express Ezh2 in mouse brain. (A) Hoechst (nuclear) stained section of mouse brain with rectangles indicating the subventricular zone (B) and the corpus callosum (C). (B–B”) Immunohistochemistry of mouse brain sections containing the subventricular zone (SVZ). (B’)  =  anti-nestin, (B”)  =  anti-Ezh2, (B”’) overlay of (B’) and (B”) and Hoechst nuclear staining (blue). (C–C”’) Immunohistochemistry of mouse brain sections containing the corpus callosum. (C’)  =  anti-RIP (RIP  =  oligodendrocyte marker), (C”)  =  anti Ezh2, (C”’) overlay of (C’) and (C”) and Hoechst nuclear staining. (**E**–**H**) ***OPCs in corpus callosum express Ezh2 during demyelination and remyelination in cuprizone fed mice.*** (**D**) Hoechst (nuclear) stained section of mouse brain with corpus callosum indicated (**E**, **F**). (**E**–**E”’**) Immunohistochemistry on corpus callosum sections of five weeks cuprizone fed mice (five weeks of demyelination  = 5WD) (**E’**)  =  anti-RIP, (**E”**)  =  anti-Ezh2, (**E”**’)  =  overlay of (**E’** and **E”**) and Hoechst nuclear staining (blue). (**F**–**F”’**) Immunohistochemistry on corpus callosum section of brain of mouse kept for five weeks on cuprizone diet followed by two weeks on normal diet (two weeks remyelination  = 2WR), (**F’**)  =  anti-RIP, (**F”**) anti-Ezh2, (**F”’**)  =  overlay of (**F’**) and (**F”**) and nuclear staining. (**G**) Western blot showing the expression of Ezh2 protein (91 kDa band) *in vivo*, in the tissue lysates prepared from SVZ and hippocampus of adult mouse brain, and in *in vitro* cultured mouse adult NSCs. (**H**) Western blot showing the loading control (beta-actin), Ezh2 and H3K27me3 protein bands in lysates from corpus callosum of control, 5WD and 2WR mouse brains. In the corpus callosum of control mouse brain, the Ezh2 band was below the detection level. (**I**) Western blot on the same lysates used in (**H**) but immunoblotted with an antibody against NG2 (an early oligodendrocyte marker). (Calibration bars in (**A**) and (**D**) represent 250 micrometers; calibration bars in (**B’**) and (**E’**) represent 100 micrometers and are valid for (**B’**–**C”’**) and (**E’**–**F”’**)).

To further confirm the relevance of Ezh2 expression in NSCs and during their differentiation into oligodendrocytic cell lineage, we extended our previous *in vitro* observations to an *in vivo* model. We performed Ezh2-immunostaining on adult mouse brain sections containing the subventricular zone. NSCs in this zone appeared to be specifically stained for Ezh2 (Figure 7A, and 7B–7B”’). Ezh2-immunostaining of the adjacent corpus callosum revealed only sporadically Ezh2 staining in a few oligodendrocyte precursor cells (Figure 7A and 7C–C”’) while adult oligodendrocytes did not express Ezh2. Exposing mice to a diet containing 0.2% cuprizone for five weeks results in oligodendrocyte death and demyelination in the corpus callosum. In reaction to this demyelination, OPCs from neighboring areas invade the corpus callosum and proliferate. Restoring normal diet after this period even further stimulates OPCs recruitment, evoking remyelination activity of the corpus callosum within two weeks. Immunostaining revealed the high expression of Ezh2 in the OPCs in the corpus callosum after five weeks on diet (Figure 7D, 7E–E”’ and 7H) as well as after two weeks after restoring normal diet (Figure 7D, 7F–F”’ and 7H). Western blot analysis of isolated SVZ and corpus callosum tissue confirmed the expression patterns of Ezh2 in control and cuprizone treated mice as observed in the immunohistochemical sections (Figure 7G).

## Discussion

Previously, we have shown that the PcG protein Ezh2 is prominently expressed in NSCs and in premyelinating oligodendrocytes *in vitro* [24]. We now have extended these observations with *in vivo* data on the expression of Ezh2 in adult NSCs and most prominently in OPCs, recruited for remyelination in adult mice put on a 0.2% cuprizone diet. Our ChIP-Seq analysis revealed 1532 genes targeted by Ezh2 in NSCs of which 393 remained an Ezh2 target in OPCs up to the stage of becoming a myelin-forming oligodendrocyte. The Ezh2-silencing experiments present evidence for the involvement of Ezh2 in proliferation and self-renewal in NSCs by specifically targeting genes involved in cell death, cellular compromise, and cell cycle arrest (e.g. p16 pathway) and sets of genes involved in NSC differentiation. Genes that remain targeted by Ezh2 in pOLs appear to be partly associated with cell death signaling and prominently with neuronal and astrocytic lineage determination.

The target genes of Ezh2 that we found in NSCs as well as in pOLs substantially overlap with most of the polycomb target genes conserved in embryonic stem cells (ES cells) in flies and vertebrates [43], [44]. The repression of these target genes (which include most of the Hox genes) are all considered essential for the maintenance of a pluripotent state. Our findings suggest that the same genes are required for maintaining multipotency and seem to imply that the oligodendrocyte precursor cells may not be considered merely unipotent, but still have a multipotent signature. Indeed the studies by Kondo et al. have shown that OPCs can be easily reprogrammed into NSCs [45]. Such a continuing multipotent signature during differentiation apparently does not exist during the differentiation of NSCs into neurons or astrocytes, where there is an abrupt transition from NSC to differentiated cell accompanied by an absolute downregulation of Ezh2. Forced expression of Ezh2 in astrocytes seems to be a prerequisite to induce their dedifferentiation back to NSC-like cells [25]. The gradual stage-dependent decrease in Ezh2 expression during oligodendrocyte differentiation seems to be in line with the emerging view that PcG protein complexes dynamically regulate the gene hierarchy during cell fate choice up to different levels of differentiation [46], [47]. Our findings in the OPCs are also in line with studies on the role of Ezh2 in other tissue-specific multipotent stem cells, for instance in the skin: Ezh2-expressing basal progenitor cells in the skin give rise to several epidermal cell types and by controlling spatial and temporal epigenetic modifications Ezh2 orchestrates gene expression during the differentiation into epidermis-specific cell types in a stepwise fashion [23].

Derepression of oligodendrogenic genes, while continuing the Ezh2-mediated repression of neuronal and astrocytic lineage genes, appears to be a major step in the differentiation of NSCs towards an oligodendrocytic cell lineage. Apparently, such a strategy is not followed during the differentiation into neurons or astrocytes, when both astrogenic and neurogenic genes are no longer Ezh2 target genes. Other epigenetic regulatory mechanisms may be involved: for instance, it is possible that in these cell types the homolog of Ezh2, Ezh1, may take over and execute the trimethylation of H3K27 of the relevant target genes [48]. Otherwise, the role of REST, a major regulator of oligodendrocyte differentiation, may interfere and modify the epigenetic mechanisms in differentiating neurons and astrocytes [49].

In our studies, Ezh2 knockdown resulted in a profound decrease in NSC proliferation and an increase in cell death. We conclude that Ezh2 is critical for normal NSC survival and self-renewal. This is in line with previous studies linking Ezh2 to cell survival and proliferation [22], [50], [51]. We identified several target genes of Ezh2, like *Cdkn2a*, *Tcf21*, and *Six1*, which are all involved in cell cycle control, cell death and cellular compromise. We found a strong increase in the p16 protein encoded by *Cdkn2a* after Ezh2 silencing. P16 acts as a negative regulator of proliferation of normal cells by interacting with CDK4 and CDK6. This inhibits their ability to interact with cyclin D and to phosphorylate the retinoblastoma protein and so to maintain a stable Rb-E2F complex, which represses cell cycle progression. Our data suggest that Ezh2 silencing leads to derepression of p16 transcription and subsequent promotion of cell cycle exit. It would be interesting to check whether p16 silencing can rescue the phenotype instigated by Ezh2-shRNA. To this purpose, the use of Ezh2-conditional knockout mice would be optimal. Other target genes of Ezh2 likely involved in its control of proliferation and survival of NSCs are *Gdf6* and *Gdf7* - subfamily members of the TGF-beta superfamily, which when derepressed activate TGF-beta signaling. TGF-beta signaling is involved in various physiological and pathophysiological processes of the brain. It appears to be induced in the adult brain after injury or hypoxia and during neurodegeneration when it modulates and dampens inflammatory responses [41]. TGF-beta signaling has been thought to limit the self-repair of the brain by inhibiting neural stem cell proliferation [41], [52]. Moreover, *in vitro* assays have shown that TGF-beta signaling activation resulted in a strong suppression of NSCs proliferation and self-renewal [42]. The identification of *Gdf6* and *Gdf7* as Ezh2 target genes has implicated TGF-beta signaling for the first time in the Ezh2 controlled regulation of NSC proliferation and survival. Likewise, the Ezh2 silencing experiments in pOLs showed a similar critical role of Ezh2 in survival. Similar mechanisms as described above for NSCs may be involved. In addition, it is clear that the forced expression of genes encoding for other neural cell types in a cell already committed to an oligodendrocytic cell lineage may amount to considerable cell stress resulting in apoptosis.

So far, we assumed that Ezh2 exerts the same effect in NSCs and in pOLs. The actual activity of Ezh2 is determined by the composition of the PRC2 complex and this composition may vary in different cell types. Previous studies have shown that, in general, the core components of PRC2 are interdependent [15], [40], [53] and indeed in a first experiment in our lab we have shown that the core components of PRC2 (Ezh2, Suz12 and EED) were present in both NSCs and pOLs (see Figure S3A and Figure S3B). However, in pOLs we detected the presence of an additional isoform of Suz12, possibly a sumoylated modification of this component (see Figure S3C), which may affect PRC2 activity and the resulting recruitment of PRC1 influencing the transcription of one or more genes in a differential way. In future research, the specific regulation of the expression of each of the Ezh2 target genes should be addressed to understand in detail the role of PcG epigenetic regulation in oligodendrocyte differentiation.

## Supporting Information

Figure S1 **Knock down of Ezh2 expression by two independent Ezh2-shRNAs phenocopy each other’s effect in Ezh2 expressing neural cells. Part 1.** (**A**) Western blot showing the level of expression of Ezh2 in *in vitro* cultured neural stem cells (NSCs), pre-myelinating oligodendrocytes (pOLs), astrocytes and Oli-neu cell line (oligodendrocyte precursor cells). Astrocytes are lacking the expression of Ezh2. (**B**) Western blot. Five clones (sh-1 to sh-5) of Ezh2 short hairpin RNA (targeting different sequences in the coding region of Ezh2) plasmid DNA (cloned in pLKO.1-puro vector) were tested independently. Non-target shRNA (nc-shRNA) pLKO.1-puro vector containing a shRNA insert that does not target any human or mouse gene, was used as control (nc). Two (sh-4 & sh-5) out of five clones efficiently downregulated Ezh2 expression. Sh-4 & sh-5 were used for further experiments. (**C**) Western blot shows the loss of function of Ezh2. A clear reduction in histone 3 lysine 27 trimethylation (H3K27me3) can be seen in cells transfected with either sh-4 or sh-5 versus control shRNA (nc-shRNA). ***Effect of Ezh2-shRNA on cell morphology.*** (**D**) NSCs transfected with Ezh2-shRNA (sh-4 or sh-5) showed reduction in neurosphere size (a–a”) in comparison to the NSCs transfected with nc-shRNA. However, in astrocytes, which do not express Ezh2 (see (A)), transfection with Ezh2-shRNA had no effect on cell morphology (b–b”). Ezh2-shRNA in comparison to the nc-shRNA showed dramatic effect on the morphology of pOLs (c–c”) and Oli-neu (d–d”) both express high levels of Ezh2, as shown on the Western blot in A. Retraction of cell extensions can be clearly seen. (Calibration bar in (Da) represents 100 micrometers and it is valid for all the photomicrographs in this figure).
(TIF)

Figure S2 **Knock down of Ezh2 expression by two independent Ezh2-shRNAs phenocopy each other’s effect in Ezh2 expressing neural cells. Part 2.** (**A**) Western blot shows that two independent Ezh2-shRNAs (sh-4 & sh-5) targeting two different sequences in the coding region of Ezh2 gene have the same effect in neural stem cells (NSCs) and pre-myelinating oligodendrocytes (pOLs) (i.e. induce apoptosis as shown by the upregulation of the apoptotic marker cleaved caspase 3 (17–19 kDa band)). (**B**) Immunocytochemistry shows transfection of NSCs (nestin positive cells) and pOLs (RIP (anti oligodendrocyte) positive cells) either with sh-4 or sh-5 results in the increase in apoptotic cells (cleaved caspase 3 positive cells). Calibration bar in (B) represents 100 um, which is valid for all the photomicrographs
(TIF)

Figure S3 **Core components of PRC2 (Ezh2, Eed, & Suz12) are conserved between NSCs and pOLs.** (**A**-**B**) Immunocytochemistry of cultured NSCs and pOLs shows the expression of Ezh2, Suz12 and Eed. (Msi  =  anti-musashi, a NSC marker; RIP  =  anti-CNPase, an oligodendrocyte marker; Hoechst  =  nuclear staining, blue). (**C**) Western blot showing the bands of Ezh2, Suz12, Eed and beta-actin at expected heights. White arrow points at the additional band of Suz12 in pOLs, not present in NSCs and ESCs. (ESCs  =  embryonic stem cells; positive controls). (Calibration bars in (**A**) and (**B**) represent 50 micrometers and they are valid for all the photomicrographs in this figure).
(TIF)

Figure S4 **Detailed depiction of nc-shRNA (control) and Ezh2-shRNA.** The shRNA hairpin is cloned into the AgeI and EcoRI site of the pLK0.1 vector. The CCGG is left over from the AgeI site and this site is lost during the cloning. The CTCGAG is the “loop” sequence. The TTTTT is the Polymerase III stop sequence. The 3′ G, if present, indicates an intact EcoRI site. The Ezh2-shRNA clone has EcoRI still intact. The sense hairpin sequence (RNAi sequence) is in red and antisense is in purple.
(TIF)

Figure S5 **UCSC Genome Browser visual after loading BigWig tracks of ChIP-Seq-Ezh2 and ChIP-Seq-IgG control.** The image displays two different 50 kb regions of mouse chromosome 11. The upper track (green) represent ChIP-Seq-IgG control while lower track represent ChIP-Seq-Ezh2 (NSCs). *Lhx1, Igf2bp1* and *GiP* were identified as gene peaks by MACS. However, while the regions near or in the promoter of the *Lhx1, Igf2bp1* show considerable high ChIP signal (high number of reads) in ChIP-Seq-Ezh2 versus ChIP-Seq-IgG control track, such difference did not exist in or near the promoter of GiP (large arrow). Therefore GiP peak was concluded as false positive. Directions of small arrows indicate orientation of the transcription.
(TIF)

Table S1 **Primers used for quantitative PCR.**
(DOCX)

Table S2 **Primers used for ChIP experiments.**
(DOCX)

Table S3 **Antibodies used.**
(DOCX)

Table S4 **ChIP-Seq data.**
(DOCX)

Information S1 **Supplementary Materials and Methods.**
(DOCX)

Information S2 **Target gene list NSC.**
(TXT)

Information S3 **Target gene list pOLs.**
(TXT)

Information S4 **ChIP-Seq IgG control FASTQC report.**
(PDF)

Information S5 **ChIP-Seq NSCs FASTQC report.**
(PDF)

Information S6 **ChIP-Seq pOLs FASTQC report.**
(PDF)
