# Supplementary figures and images for: Dynamic Changes in Ezh2 Gene Occupancy Underlie Its Involvement in Neural Stem Cell Self-Renewal and Differentiation towards Oligodendrocytes
Source: PLoS One. 2012 Jul 12;7(7):e40399. doi: 10.1371/journal.pone.0040399 (PMC3395718; doi:10.1371/journal.pone.0040399)

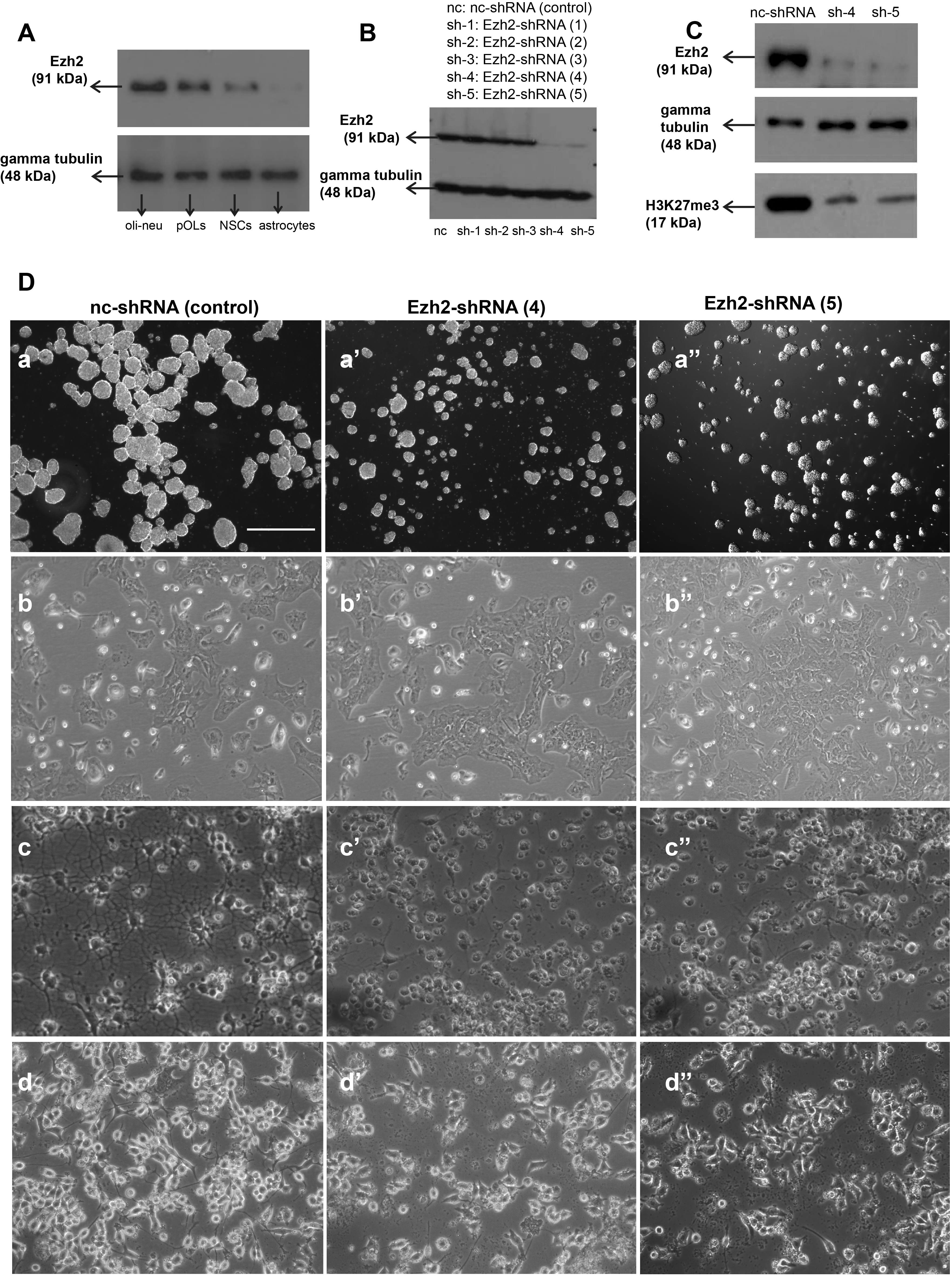

Supplement: Figure S1 — Knock down of Ezh2 expression by two independent Ezh2-shRNAs phenocopy each other’s effect in Ezh2 expressing neural cells. Part 1. (A) Western blot showing the level of expression of Ezh2 in in vitro cultured neural stem cells (NSCs), pre-myelinating oligodendrocytes (pOLs), astrocytes and Oli-neu cell line (oligodendrocyte precursor cells). Astrocytes are lacking the expression of Ezh2. (B) Western blot. Five clones (sh-1 to sh-5) of Ezh2 short hairpin RNA (targeting different sequences in the coding region of Ezh2) plasmid DNA (cloned in pLKO.1-puro vector) were tested independently. Non-target shRNA (nc-shRNA) pLKO.1-puro vector containing a shRNA insert that does not target any human or mouse gene, was used as control (nc). Two (sh-4 & sh-5) out of five clones efficiently downregulated Ezh2 expression. Sh-4 & sh-5 were used for further experiments. (C) Western blot shows the loss of function of Ezh2. A clear reduction in histone 3 lysine 27 trimethylation (H3K27me3) can be seen in cells transfected with either sh-4 or sh-5 versus control shRNA (nc-shRNA). Effect of Ezh2-shRNA on cell morphology. (D) NSCs transfected with Ezh2-shRNA (sh-4 or sh-5) showed reduction in neurosphere size (a–a”) in comparison to the NSCs transfected with nc-shRNA. However, in astrocytes, which do not express Ezh2 (see (A)), transfection with Ezh2-shRNA had no effect on cell morphology (b–b”). Ezh2-shRNA in comparison to the nc-shRNA showed dramatic effect on the morphology of pOLs (c–c”) and Oli-neu (d–d”) both express high levels of Ezh2, as shown on the Western blot in A. Retraction of cell extensions can be clearly seen. (Calibration bar in (Da) represents 100 micrometers and it is valid for all the photomicrographs in this figure). (TIF) [file pone.0040399.s001.tif]

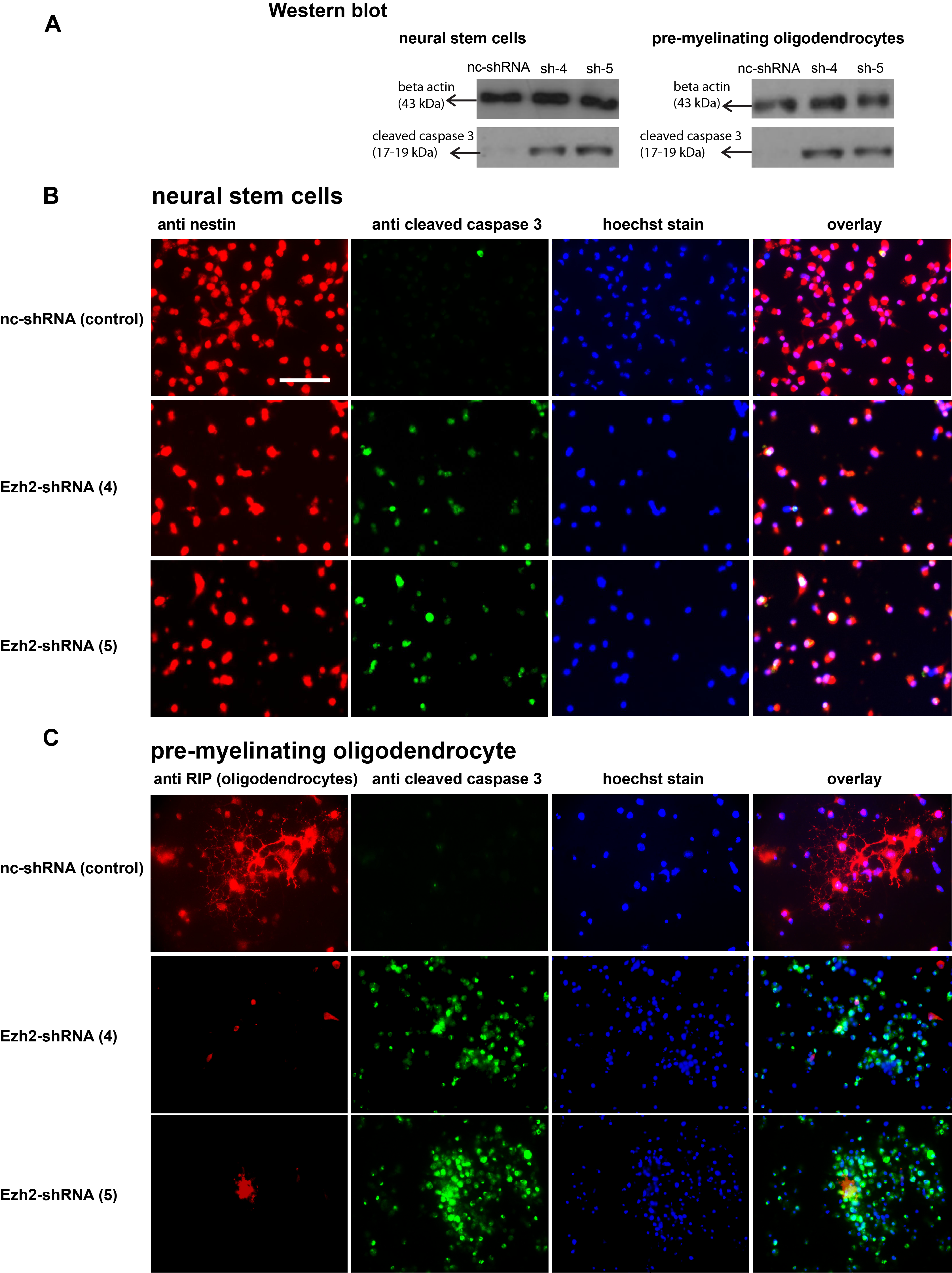

Supplement: Figure S2 — Knock down of Ezh2 expression by two independent Ezh2-shRNAs phenocopy each other’s effect in Ezh2 expressing neural cells. Part 2. (A) Western blot shows that two independent Ezh2-shRNAs (sh-4 & sh-5) targeting two different sequences in the coding region of Ezh2 gene have the same effect in neural stem cells (NSCs) and pre-myelinating oligodendrocytes (pOLs) (i.e. induce apoptosis as shown by the upregulation of the apoptotic marker cleaved caspase 3 (17–19 kDa band)). (B) Immunocytochemistry shows transfection of NSCs (nestin positive cells) and pOLs (RIP (anti oligodendrocyte) positive cells) either with sh-4 or sh-5 results in the increase in apoptotic cells (cleaved caspase 3 positive cells). Calibration bar in (B) represents 100 um, which is valid for all the photomicrographs (TIF) [file pone.0040399.s002.tif]

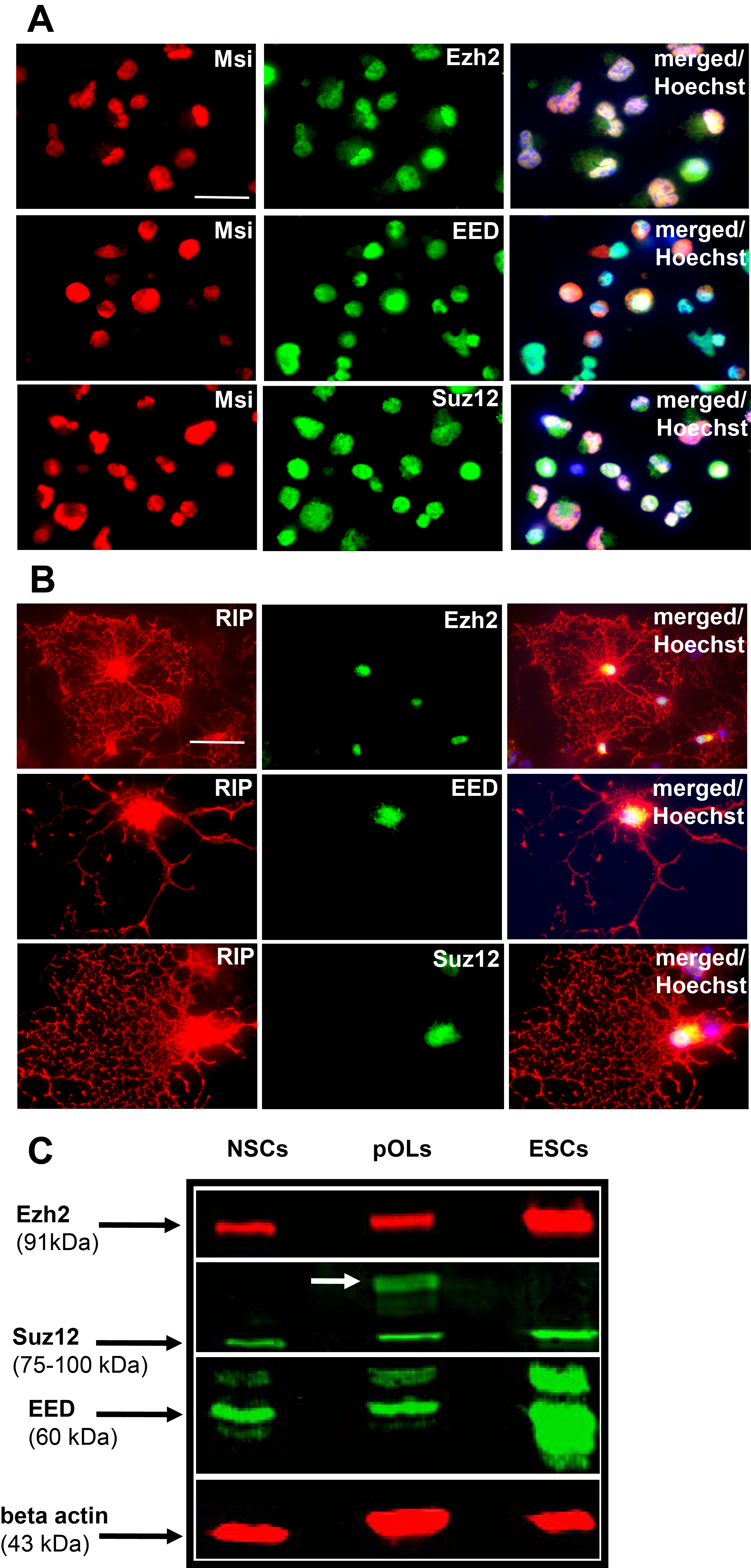

Supplement: Figure S3 — Core components of PRC2 (Ezh2, Eed, & Suz12) are conserved between NSCs and pOLs. (A-B) Immunocytochemistry of cultured NSCs and pOLs shows the expression of Ezh2, Suz12 and Eed. (Msi = anti-musashi, a NSC marker; RIP = anti-CNPase, an oligodendrocyte marker; Hoechst = nuclear staining, blue). (C) Western blot showing the bands of Ezh2, Suz12, Eed and beta-actin at expected heights. White arrow points at the additional band of Suz12 in pOLs, not present in NSCs and ESCs. (ESCs = embryonic stem cells; positive controls). (Calibration bars in (A) and (B) represent 50 micrometers and they are valid for all the photomicrographs in this figure). (TIF) [file pone.0040399.s003.tif]

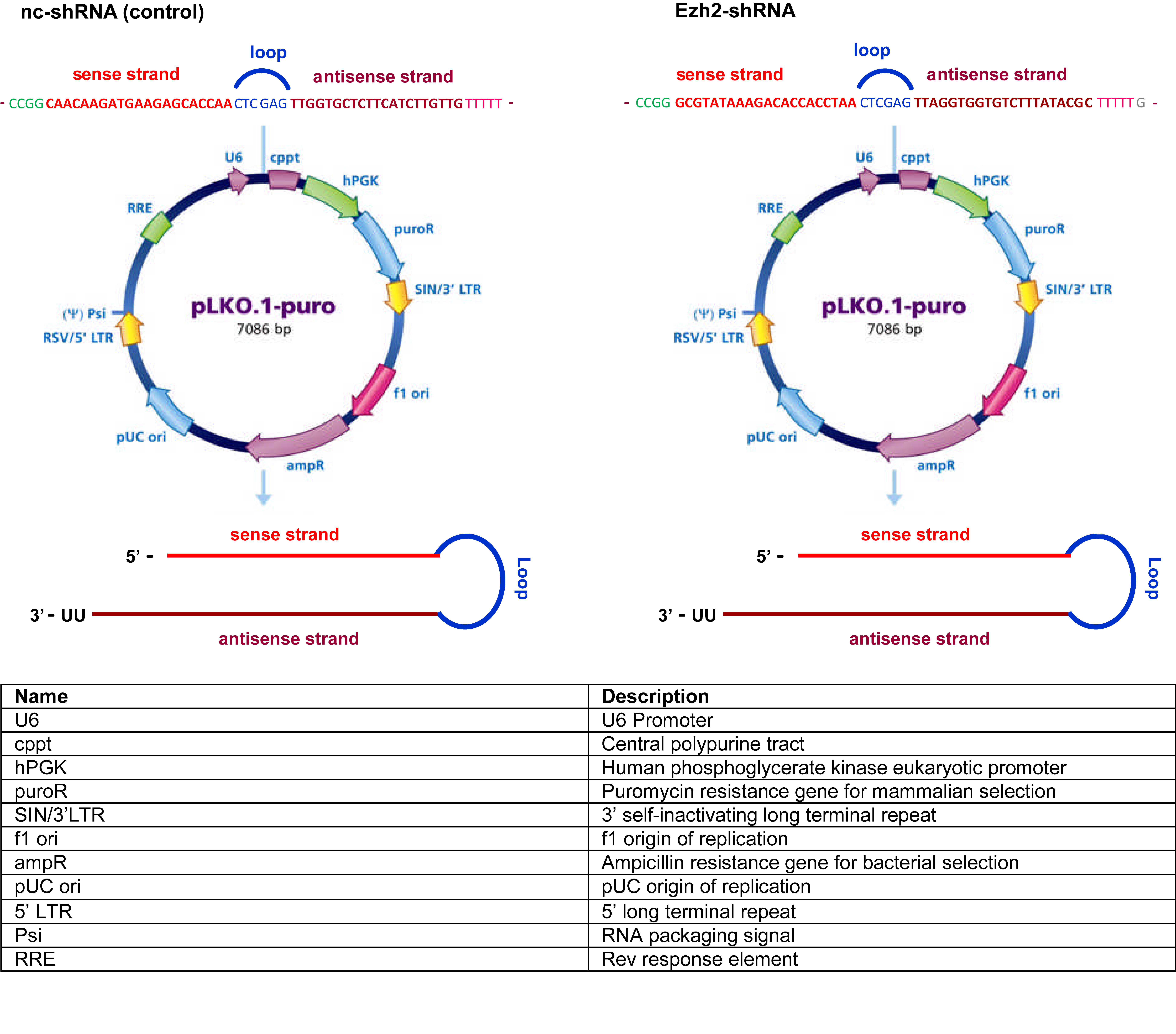

Supplement: Figure S4 — Detailed depiction of nc-shRNA (control) and Ezh2-shRNA. The shRNA hairpin is cloned into the AgeI and EcoRI site of the pLK0.1 vector. The CCGG is left over from the AgeI site and this site is lost during the cloning. The CTCGAG is the “loop” sequence. The TTTTT is the Polymerase III stop sequence. The 3′ G, if present, indicates an intact EcoRI site. The Ezh2-shRNA clone has EcoRI still intact. The sense hairpin sequence (RNAi sequence) is in red and antisense is in purple. (TIF) [file pone.0040399.s004.tif]

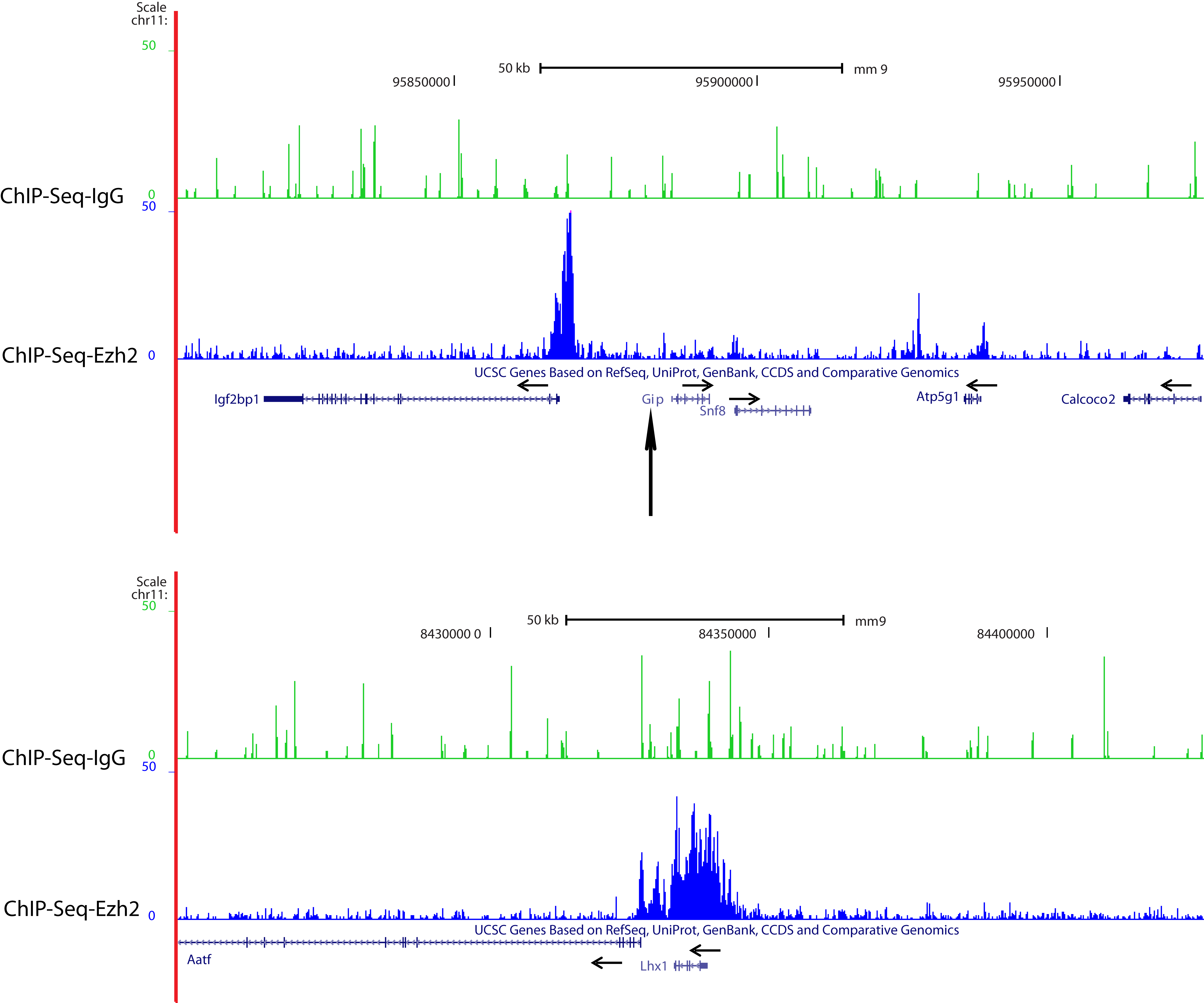

Supplement: Figure S5 — UCSC Genome Browser visual after loading BigWig tracks of ChIP-Seq-Ezh2 and ChIP-Seq-IgG control. The image displays two different 50 kb regions of mouse chromosome 11. The upper track (green) represent ChIP-Seq-IgG control while lower track represent ChIP-Seq-Ezh2 (NSCs). Lhx1, Igf2bp1 and GiP were identified as gene peaks by MACS. However, while the regions near or in the promoter of the Lhx1, Igf2bp1 show considerable high ChIP signal (high number of reads) in ChIP-Seq-Ezh2 versus ChIP-Seq-IgG control track, such difference did not exist in or near the promoter of GiP (large arrow). Therefore GiP peak was concluded as false positive. Directions of small arrows indicate orientation of the transcription. (TIF) [file pone.0040399.s005.tif]
