# Supplementary material for: Dynamic Changes in Ezh2 Gene Occupancy Underlie Its Involvement in Neural Stem Cell Self-Renewal and Differentiation towards Oligodendrocytes
Source: PLoS One. 2012 Jul 12;7(7):e40399. doi: 10.1371/journal.pone.0040399 (PMC3395718; doi:10.1371/journal.pone.0040399)
Supplement: Table S1 — Primers used for quantitative PCR. (DOCX) [file pone.0040399.s006.docx]

**Table S1.** Primers used for quantitative PCR

| **Gene (NCBI Symbol)** | **FW Sequence (5' -> 3')** | **BW Sequence (5' -> 3')** |
| --- | --- | --- |
| Neurod2 | AAGCCAGTGTCTCTTCGTGG | GCCTTGGTCATCTTGCGTTT |
| Phox2b | GGGCTAAGTTTCGCAAGCAG | CAGTGCTGTCGGGATCAGTG |
| Six1 | ATGCTGCCGTCGTTTGGTT | CCTTGAGCACGCTCTCGTT |
| En1 | CGTCCGTCCTCTGGTCCACG | GCGGCCGCTTGTCTTCCTTC |
| En2 | CCGGCCTTCTTCAGGTCCCA | GCGAGGCCGCTTGTCCTCTT |
| Tal1 | GACCCGCAACTAGAGGGAC | CCGTTGAGCAGGACTAGGT |
| Otp | CTAGCGCTGCGTATCGGGCT | TGGAACCAGACCTGCACCCG |
| Tlx3 | GCCACCCAAGCGTAAGAAG | CAGGTACTTTTGGCGATGGAA |
| Nkx2-2 | AAGCATTTCAAAACCGACGGA | CCTCAAATCCACAGATGACCAGA |
| Nkx6.2 | AAGTCTGCCCCGTCTCAAC | GGTCTGCTCGAAAGTCTTCTC |
| Olig2 | TCCCCAGAACCCGATGATCTT | CGTGGACGAGGACACAGTC |
| Olig3 | GGAGAGTCGTCTGAACTCGG | CTGGACCATATCGCCCTGTG |
| Gdf6 | TATCGCGCCCCTAGAGTACG | ATGCTAATGGGAGTCAGTTTGG |
| Gdf7 | GAGGGCGTTTGCGACTTTC | CTGCTTGTAGACCACGTTGTT |
| Cdkn2a | CGCAGGTTCTTGGTCACTGT | TGTTCACGAAAGCCAGAGCG |
| Tcf21 | CCCACTAAGAAAAGCCCGCTC | CCGTTCTCGTACTTGTCGTTG |
| Pdgfra | TATCCTCCCAAACGAGAATGAGA | GTGGTTGTAGTAGCAAGTGTACC |
| Rpl32 | TTAAGCGAAACTGGCGGAAAC | TTGTTGCTCCCATAACCGATG |
