# Supplementary material for: Dynamic Changes in Ezh2 Gene Occupancy Underlie Its Involvement in Neural Stem Cell Self-Renewal and Differentiation towards Oligodendrocytes
Source: PLoS One. 2012 Jul 12;7(7):e40399. doi: 10.1371/journal.pone.0040399 (PMC3395718; doi:10.1371/journal.pone.0040399)
Supplement: Table S2 — Primers used for ChIP experiments. (DOCX) [file pone.0040399.s007.docx]

**Table S2.** Primers used for ChIP experiments

| **Region** | **FW Sequence (5' -> 3')** | **BW Sequence (5' -> 3')** |
| --- | --- | --- |
| Pdgfr-a (1) | TCAAAAACCCATCATCTTCCT | TCTGGGCCTCGCTAGAAATA |
| Pdgfr-a (2) | GTGTGTGTGTGGAGGGGAAA | AGTCTGGCCCCAGATGTTT |
| Tal1 | AGGCTGGTTTCGTTATGTCG | TGTTGGACAGGACCACACAT |
| Olig2 | AATTAGCCGGGTGACATCAG | GGTTCCGCTGGTTTTTATAGC |
| Olig3 | CATAAGGCCGCATCTCTTGT | AGGAGACCGAGTTCAGACGA |
| Nkx2-2 | GGACGTTGCTTCCTGAAAAA | AACCCGTTAGCATTCAGCAC |
| Nkx6-2 | CAGTGCCTTCAGCCAATCAG | CTACGGGAAAGCGAGAGGAC |
| Phox2b | AAAAAGCGCCAGCAATAAGA | CTCCACACTGGCCTCCTTAC |
| Six1 | CGGCAGGCGGGAGCCAGCGA | CCTCGGCGGGTGGGAGGCCA |
| Tlx3 | GCCTGTCGGGCCCGCGCACT | GGCTGGGCGGGGAGGCTGCT |
| Otp | CGAGGCCAAGGTGGCCGCTGCT | AGTCCCCCACTCCGCCCGCC |
| En1 | GGGGGAGGGGCGTGGGCACAA | GCGCGCGCGGGACTTTGCGG |
| En2 | ACCGAAGGAGGCGGCGGCGG | CCAGCGGCGGCGGAGAGCGT |
| Cdkn2a | TGTACAGAATCCTAGCACTGATACAGCAAC | GATCCCAACAACCCTAGCTCAAACAAC |
| Neurod2 | GTATGCCTAGGATGGGCTGA | TGGGAAAAGGTCACAGGTTC |
| Rpl32 | CATTTCTCAGGCACATCTTAGTT | GAGTCTTAGGTCCTGGCAGA |
