# Supplementary material for: Dynamic Changes in Ezh2 Gene Occupancy Underlie Its Involvement in Neural Stem Cell Self-Renewal and Differentiation towards Oligodendrocytes
Source: PLoS One. 2012 Jul 12;7(7):e40399. doi: 10.1371/journal.pone.0040399 (PMC3395718; doi:10.1371/journal.pone.0040399)
Supplement: Table S3 — Antibodies used. (DOCX) [file pone.0040399.s008.docx]

**Table S3.** Antibodies used

| **Primary Antibody Name/Product Name** | **Host species** | **Company** | **Cat. No.** |
| --- | --- | --- | --- |
| Anti Ezh2 (WB, Immuno-histo-cytochemisry) | Mouse | BD Transduction Laboratories™ | 612666 |
| Anti Ezh2 (ChIP Grad) | Rabbit | Diagenode | CS-039-100 |
| Rabbit Control IgG - ChIP Grade (Isotype controls) |  | Abcam | ab46540 |
| Anti-EED antibody | Rabbit | Millipore | 09-774 |
| Anti-Suz12 antibody | Rabbit | Diagenode | CS-029-100 |
| Beta Actin antibody | Mouse | Abcam | ab6276 |
| Anti-Oligodendrocytes, clone NS-1 (RIP) | Mouse | Millipore | MAB1580 |
| Anti-Musashi-1 | Rabbit | Millipore | AB5977 |
| ChIPAb+ Trimethyl-Histone H3 (Lys27) | Rabbit | Millipore | 17-622 |
| Ki67 antibody - Proliferation Marker | Rabbit | Abcam | ab15580 |
| P16 (Cdkn2a) | Mouse | SANTA CRUZ BIOTECHNOLOGY, INC. | sc-1661 |
| Anti Smad (H-465) antibody | Rabbit | SANTA CRUZ BIOTECHNOLOGY, INC. | sc-7153 |
| Human/Mouse GLI-1 MAb (Clone 388516), | Rat | R & D | MAB3324 |
| Anti PDGFR-α antibody | Rabbit | SANTA CRUZ BIOTECHNOLOGY, INC. | SC-338 |
| Cleaved Caspase-3 | Rabbit | Cell Signaling Technology | 9664S |
| PCNA antibody-Proliferation Marker | Mouse | Abcam | ab29 |
| Gamma Tubulin antibody | Mouse | Abcam | ab11316 |
| Anti-beta catenin antibody | Mouse | The Developmental Studies Hybridoma Bank | PY654 |
| Anti-NG2 Chondroitin Sulfate Proteoglycan (NG2) | Rabbit | Millipore | AB5320 |
| Beta Actin antibody | Rabbit | Abcam | ab8227 |
| Anti-Nestin | Mouse | Millipore | MAB353 |
|  |  |  |  |
| **Secondary Antibody Name/Product Name** | **Host species** | **Company** | **Cat. No.** |
| Goat anti-Rabbit IgG, (H+L) FITC conjugate | Goat | Millipore | AP307F |
| Alexa Fluor® 488-donkey anti-rabbit IgG (H+L) | Donkey | Invitrogen | A-21206 |
| Alexa Fluor® 488-goat anti-mouse IgG (H+L) | Goat | Invitrogen | A11001 |
| Cy3 AffiniPure Goat Anti-Mouse IgG (H+L) | Goat | Jackson ImmunoResearch | 115-165-003 |
| Cy3 AffiniPure Goat Anti-Rabbit IgG (H+L) | Goat | Jackson ImmunoResearch | 111-165-003 |
| IRDye 680 Goat-anti-Rat Antibody (WB) | Goat | LI-COR Biosciences | 926-32229 |
| IRDye 680 Donkey-anti-Mouse Antibody (WB) | Donkey | LI-COR Biosciences | 926-32222 |
| IRDye 800CW Donkey-anti-Rabbit Antibody (WB) | Donkey | LI-COR Biosciences | 926-32213 |
