# Supplementary material for: Dynamic Changes in Ezh2 Gene Occupancy Underlie Its Involvement in Neural Stem Cell Self-Renewal and Differentiation towards Oligodendrocytes
Source: PLoS One. 2012 Jul 12;7(7):e40399. doi: 10.1371/journal.pone.0040399 (PMC3395718; doi:10.1371/journal.pone.0040399)
Supplement: Table S4 — ChIP-Seq data. (DOCX) [file pone.0040399.s009.docx]

**Table S4.** ChIP-Seq data

**QC and manipulation before mapping.** FASTQ Groomer was used to Filter FASTQ reads by quality score (QS) and length. Sequences having QS less than 10 qs were filtered. FASTQ Trimmer by column was applied to trim the sequences (16bp from 3 ends).

| **alignment software** | **sequence** | **total reads (sanger/Illumina 1.9)** | **after filtering out low QS reads** | **matched reads** | **average length** | **mismatches permitted** | **reference genome** |
| --- | --- | --- | --- | --- | --- | --- | --- |
| Bowtie for Illumina | ChIP-Seq IgG control | 8293125 | 3740234 | 1144753 | 60 | 2 | mm9 |
|  | ChIP-Seq - NSCs | 12948618 | 5548680 | 3727877 | 60 | 2 | mm9 |
|  | ChIP-Seq-pOLs | 11533664 | 5095659 | 1527094 | 60 | 2 | mm9 |

Complete FASTQC reports for all three sequence files can be seen as pdf files (ChIP-Seq-NSCs-FastQC Report, ChIP-Seq-pOLs-FastQC Report, ChIP-Seq-IgG-control-FastQC Report).
