# Supplementary material for: Dynamic Changes in Ezh2 Gene Occupancy Underlie Its Involvement in Neural Stem Cell Self-Renewal and Differentiation towards Oligodendrocytes
Source: PLoS One. 2012 Jul 12;7(7):e40399. doi: 10.1371/journal.pone.0040399 (PMC3395718; doi:10.1371/journal.pone.0040399)
Supplement: Information S1 — Supplementary Materials and Methods. (DOCX) [file pone.0040399.s010.docx]

**Supplementary Materials and Methods**

**Knock down of Ezh2 expression in neural stem cells (NSCs) and pre-myelinating oligodendrocytes (pOLs)**

To knock down Ezh2 expression in NSCs and pOLs we used a powerful gene-specific silencing technique that utilizes the RNA interference (RNAi) mechanism. From Sigma Mission, we purchased sequence verified viral vector (pLKO.1-puro) based short hairpin RNA library (five clones targeting different sequences in the coding regions of Ezh2 gene) against Ezh2 (Ezh2-shRNA). Sequence-verified shRNA lentiviral plasmid vectors for mouse Ezh2 gene were cloned into the pLKO.1-puro vector (see Figure S4). shRNAs are processed into siRNAs intracellularly and expressed from amphotropic lentivirus particles, resulting in efficient loss of function of the target gene.

For gene transfections we used electroporesis-based transfection protocol (Lonza, previously known as Amaxa; http: //www.lonzabio.com/cell-biology/transfection/) specifically designed for the transfection of mouse neural cell types. 2–3 million cells were transfected with 3 micrograms of shRNA plasmid DNA vector. The transfection efficiency of this procedure for mouse neural cells amounted to 60–80% and resulted in the transient expression of the transfected gene lasting up to 10–12 days. A range of knockdown efficiencies was observed. Two (we encoded them as sh-4 and sh-5) out of five clones appeared to be efficiently knocking down Ezh2 expression (see Figure S1B). The plasmids containing the following sequences

sh-4: - CCGGGCGTATAAAGACACCACCTAACTCGAGTTAGGTGGTGTCTTTATACGCTTTTTG-

sh-5: - CCGGGCACAAGTCATCCCGTTAAAGCTCGAGCTTTAACGGGATGACTTGTGCTTTTTG-

showed maximum efficiencies and were used for further experiments. For control transfections we purchased MISSION Non-Target shRNA control vector, a lentivirus plasmid vector. The vector contains the following shRNA insert

-CCGGCAACAAGATGAAGAGCACCAACTC-GAGTTGGTGCTCTTCATCTTGTTGTTTTT-

that does not target any human or mouse gene, making it useful as a negative control in experiments using the MISSION shRNA library clones. The short-hairpin sequence contains 4 base pair mismatches to any known human or mouse gene, which allows examining the effect of transfection of a short-hairpin on gene expression and interpreting the knockdown effect seen with shRNA clones. The detailed depiction of control (nc-shRNA) and Ezh2-shRNA can be seen in Figure S4.

A well-known molecular function of Ezh2 is the trimethylation of histone 3 lysine 27 (H3K27me3). We performed western blot analysis by using anti H3K27me3 antibody. We observed a clear reduction in H3K27me3 level (Figure S1C) in the NSCs transfected with either sh-4 or sh-5, compared to the control (cells transfected with nc-shRNA), indicating the specific effect of two independent Ezh2-shRNAs on the Ezh2 gene function.

To test the specific effect of Ezh2-shRNA on the phenotype of the cells we used different neural cell types. Figure S1A shows on western blot the level of expression of Ezh2 in cultured NSCs, pOLs, astrocytes and Oli-neu cell line (oligodendrocyte precursor cells). Astrocytes lack the expression of Ezh2. Transfection of Ezh2-shRNAs (sh-4 or sh-5) resulted in a clear reduction in size of the neurospheres in comparison to the nc-shRNA (S1Da/a’/a’’). However in astrocytes, which do not express Ezh2, transfection of the Ezh2-shRNA had no effect on the cell morphology (S1Db/b’/b’’). Similarly both Ezh2-shRNAs in comparison to the nc-shRNA showed dramatic effect on the morphology of pOLs (S1Dc/c’/c’’) and Oli-neu (S1Dd/d’d’’) (both express high levels of Ezh2 as shown on the western blot in Figure S1A). pOLs and the Oli-neu cells retracted their extensions, became rounded and started to die after 24 hours of transfection. These experimental data indicate that Ezh2 is necessary for the proper functioning and survival of the Ezh2 expressing neural cell types.

To further assess the indispensability of Ezh2 in neural stem cells (NSCs) and pre-myelinating oligodendrocytes (pOLs) (the two neural cell type that express high levels of Ezh2) we knocked down endogenous Ezh2 expression with specific shRNAs in NSCs and pOLs. To exclude the possibility of any off-target effects of the transfected shRNAs, two Ezh2-shRNAs (sh4 and sh5), known to target different sequences within the coding region of the Ezh2 gene, have been used. Knocking down Ezh2 with either sh-4 or sh-5 in proliferating NSCs and differentiating pOLs resulted in the upregulation of the apoptotic marker cleaved caspase 3 in these cells (shown by Western blot in Figure S2A and by immunocytochemistry in Figure S2B and Figure S2C). These results demonstrate that knock down of endogenous Ezh2 in NSCs and pOLs induces their apoptosis. Moreover, we ascertained that the observed phenomenon is truly due to the down regulation of Ezh2, rather than some unknown off target effect of the applied shRNAs, since both sh-4 and sh-5 shRNAs (targeting different parts of the Ezh2 gene) showed the same effect (i.e. induction of apoptosis in NSCs and pOLs) while the scrambled non-coding shRNA had no effect at all.

**Peak detection**

Model-based Analysis of ChIP-Seq (MACS) tool (<http://cistrome.org/ap/root>) was used to identify the peaks in the mapped reads. We used ChIP-Seq-Ezh2 and ChIP-Seq-IgG (control) BAM files as input to MACS. MACS detected 1571 regions in ChIP-Seq-NSCs and 607 regions in ChIP-Seq-pOLs as peak regions by setting ChIP-Seq-IgG as a control. We used the “peak to gene” function of the MACS for the peak annotations. To avoid redundancies, we performed visual inspection of each annotated gene peak detected by MACS on UCSC Genome Browser by loading BAM and BigWig tracks of ChIP-Seq-Ezh2 and ChIP-Seq-IgG control (comparison of ChIP signal between ChIP-Seq-Ezh2 and ChIP-Seq-IgG control). By visualizing each gene peak we found, that some peaks identified by MACS occurred in the regions where no substantial difference between the IgG control and ChIP could be observed. We concluded such peaks as false positives and were ignored while preparing the final list. For instance Lhx1, Igf2bp1 and GiP were identified as gene peaks by MACS. During the UCSC Genome Browser visual inspection we found that the track ChIP-Seq-Ezh2 shows high ChIP signal (high number of reads) versus ChIP-Seq-IgG in or near the promoter regions of Lhx1 and Igf2bp1 (Figure S5). However, there is no considerable difference in ChIP signal between ChIP-Seq-Ezh2 and ChIP-Seq-IgG control in or near the promoter region of GiP. Therefore while preparing the final list of target genes of Ezh2, we ignored GiP and the other peaks showing no considerable difference between ChIP-Seq-Ezh2 and ChIP-Seq-IgG tracks on UCSC Genome Browser.
