# Supplementary material for: Dynamic Changes in Ezh2 Gene Occupancy Underlie Its Involvement in Neural Stem Cell Self-Renewal and Differentiation towards Oligodendrocytes
Source: PLoS One. 2012 Jul 12;7(7):e40399. doi: 10.1371/journal.pone.0040399 (PMC3395718; doi:10.1371/journal.pone.0040399)
Supplement: Information S4 — ChIP-Seq IgG control FASTQC report. (PDF) [file pone.0040399.s013.pdf]

FastQC Report

Wed 13 Jul 2011

ChIP-Seq IgG control.fastqsanger

Summary

- Basic Statistics
- Per base sequence quality
- Per sequence quality scores
- Per base sequence content
- Per base GC content
- Per sequence GC content
- Per base N content
- Sequence Length Distribution
- Sequence Duplication Levels
- Overrepresented sequences
- Kmer Content

Basic Statistics

Measure	Value
Filename	ChIP-Seq IgG control.fastqsanger
File type	Conventional base calls
Encoding	Sanger / Illumina 1.9
Total Sequences	3740234
Sequence length	60
%GC	44

Back to summary

Per base sequence quality

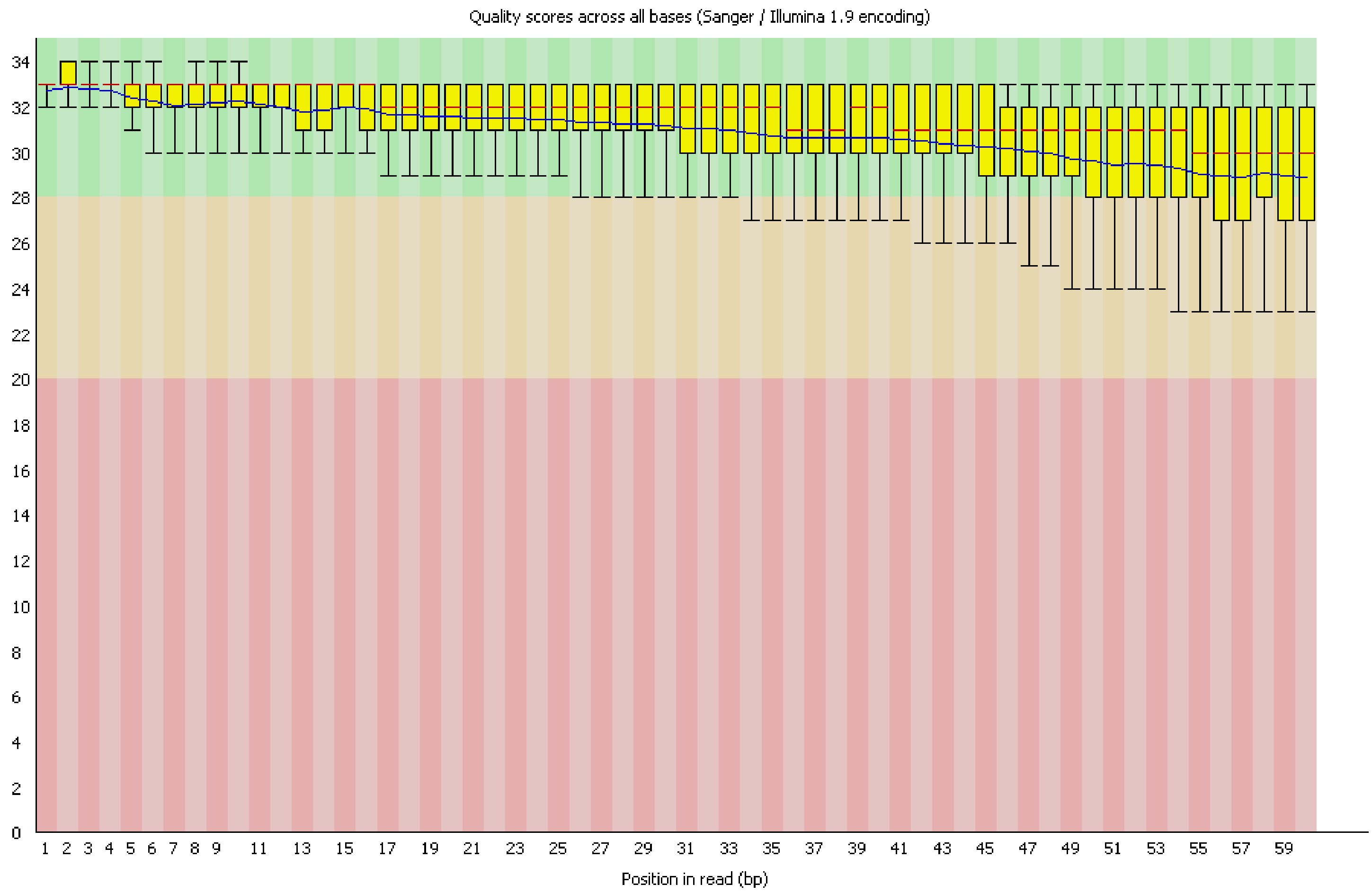

Back to summary

Per sequence quality scores

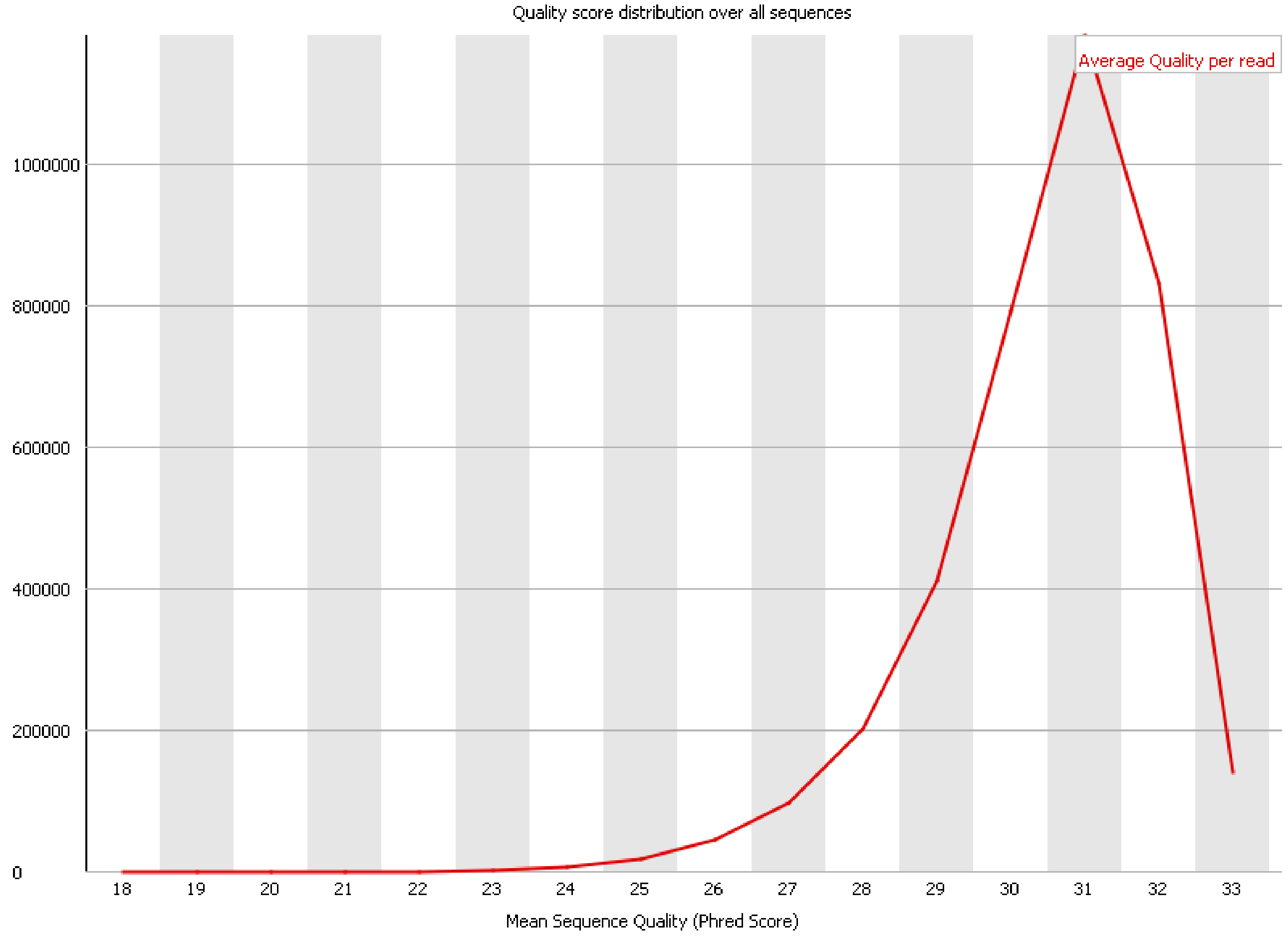

[Back to summary]

Per base sequence content

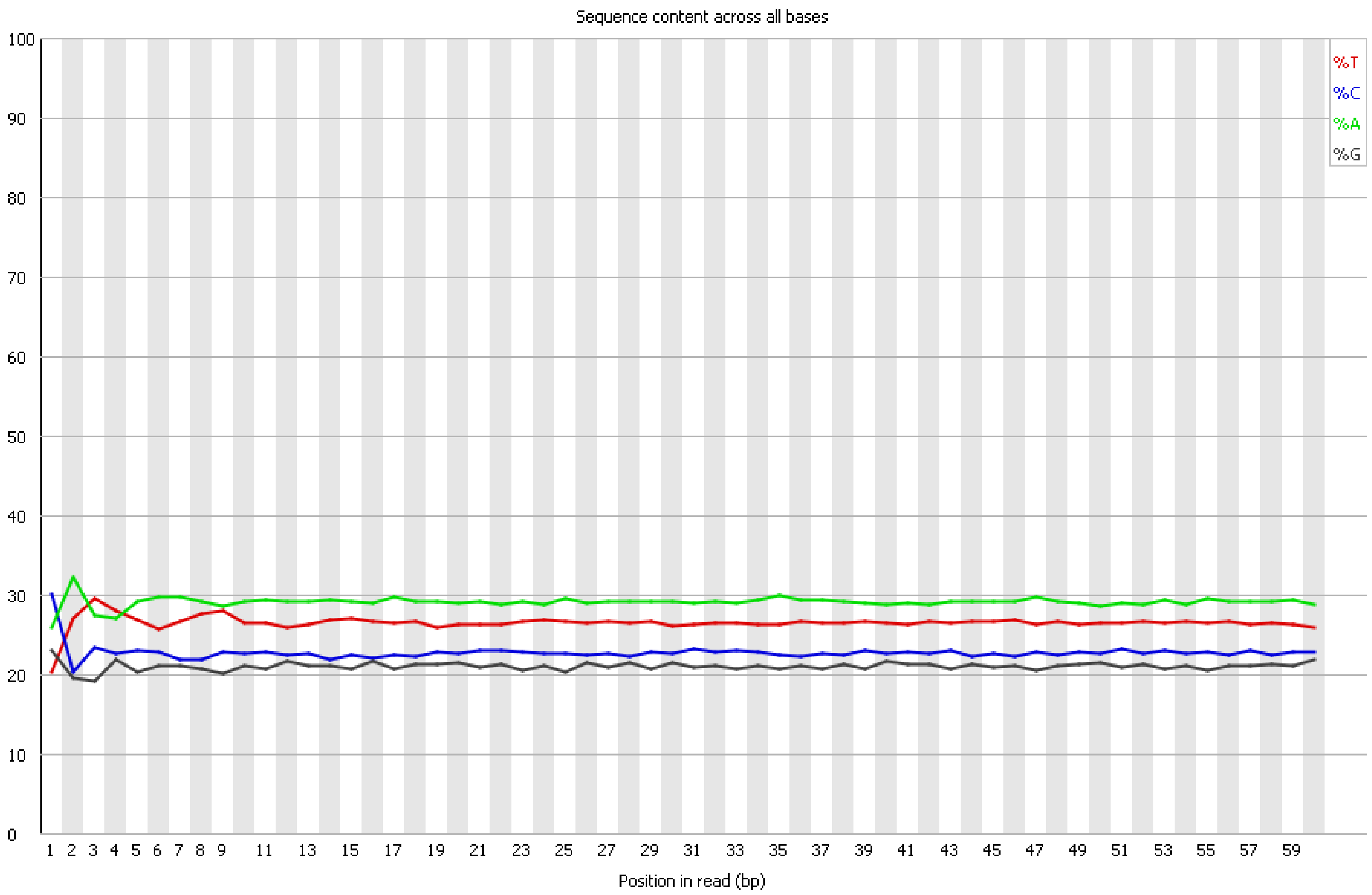

[Back to summary]

Per base GC content

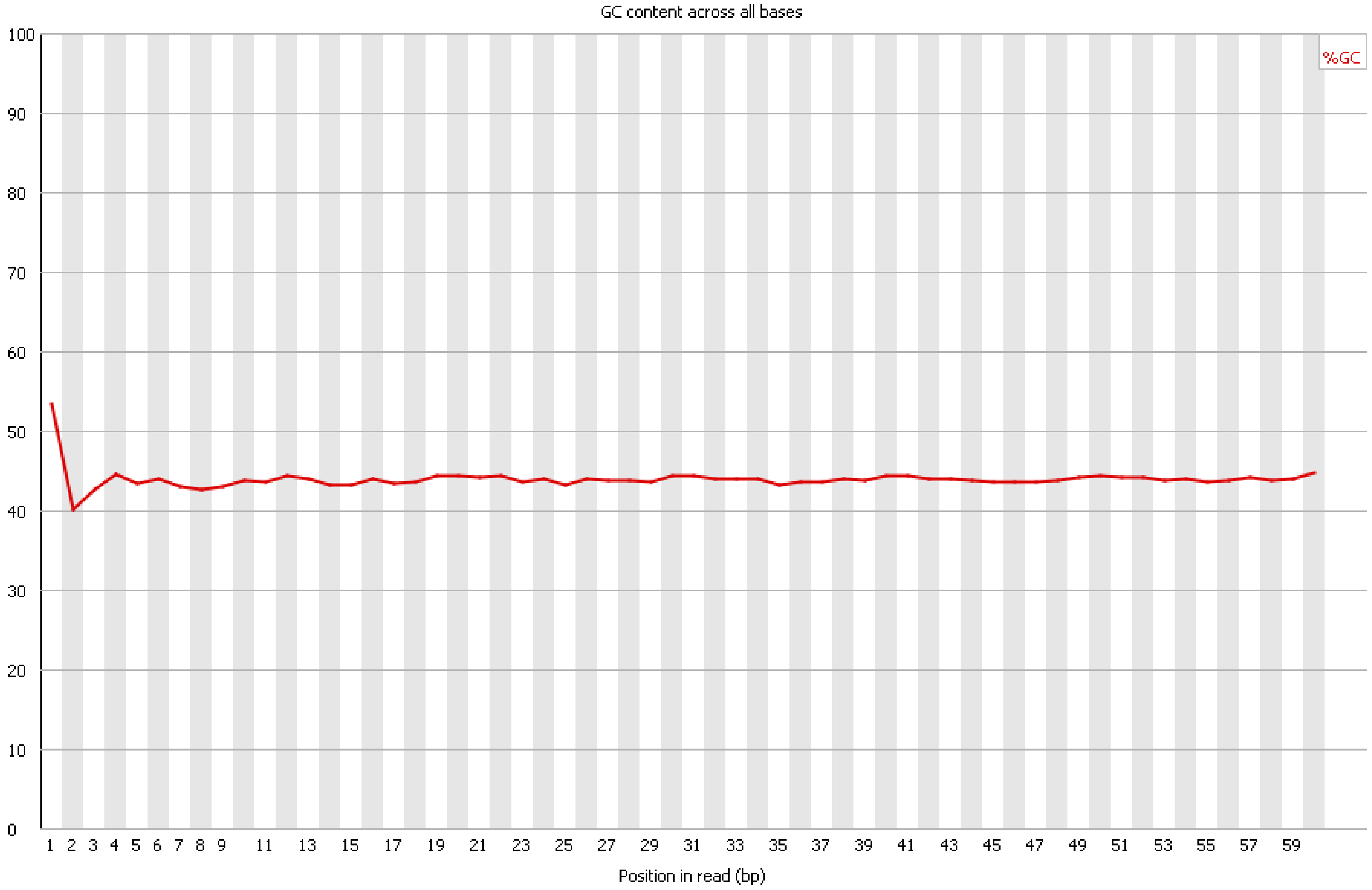

Back to summary

Per sequence GC content

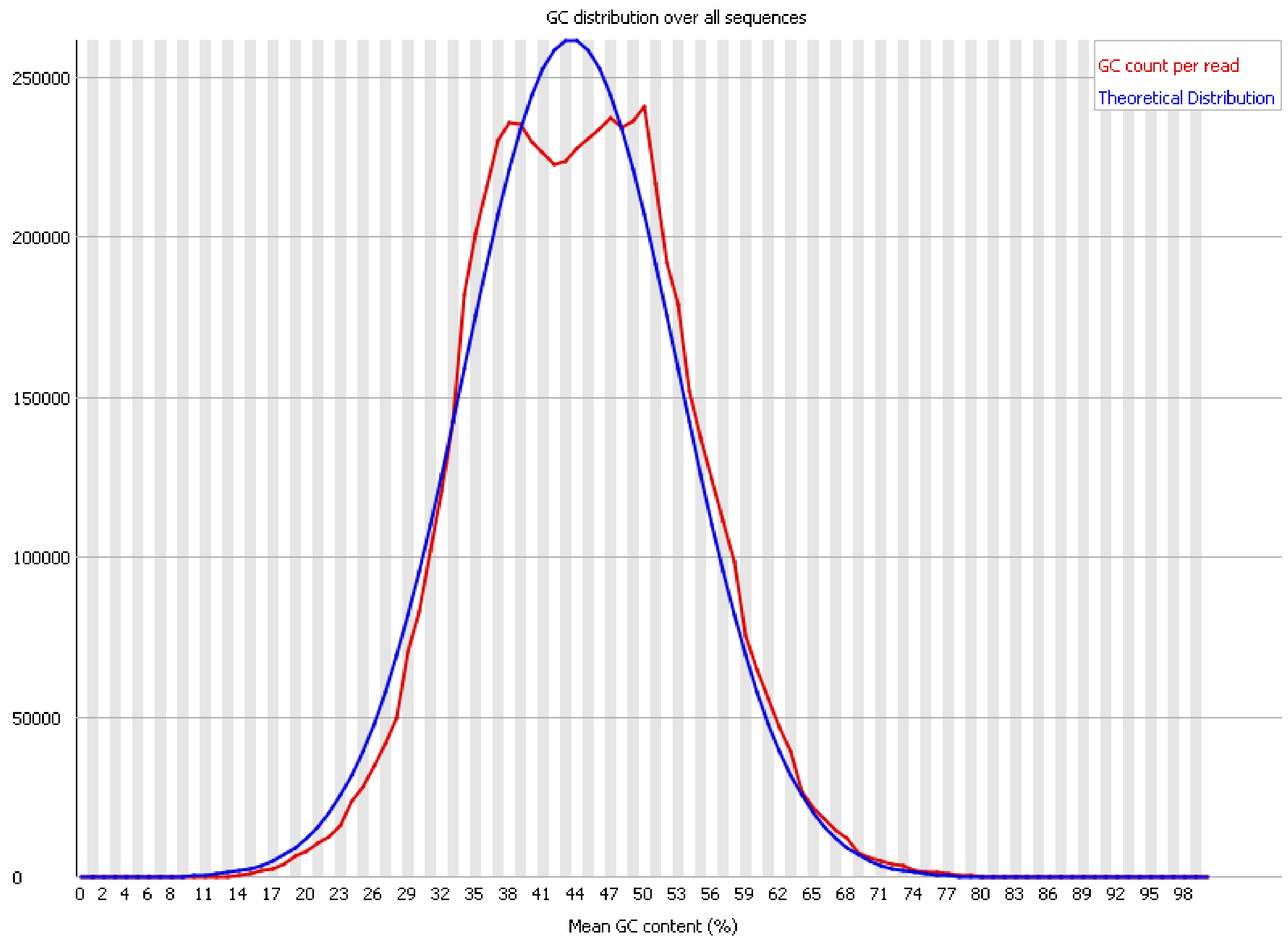

[Back to summary]

Per base N content

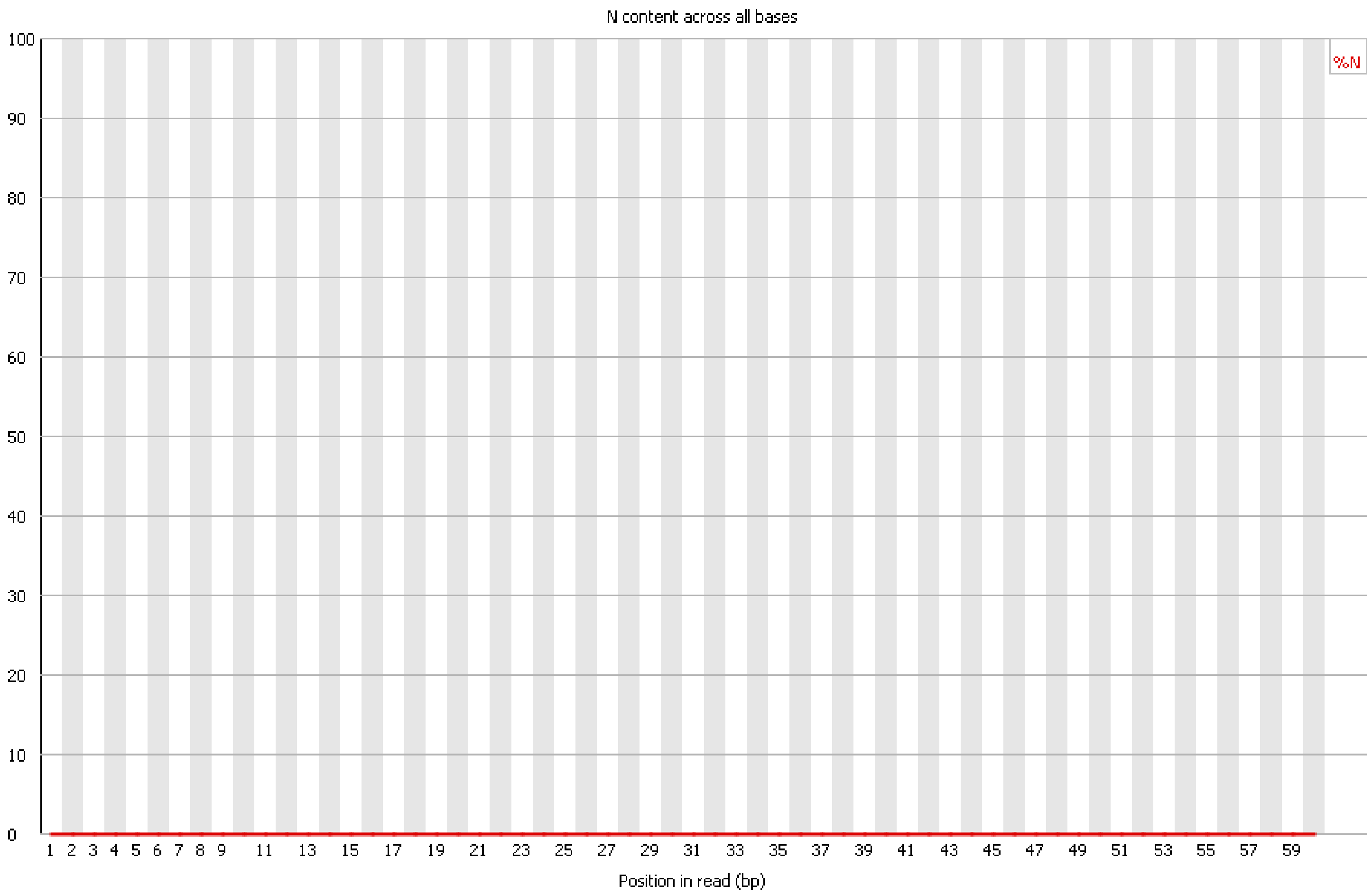

Back to summary

Sequence Length Distribution

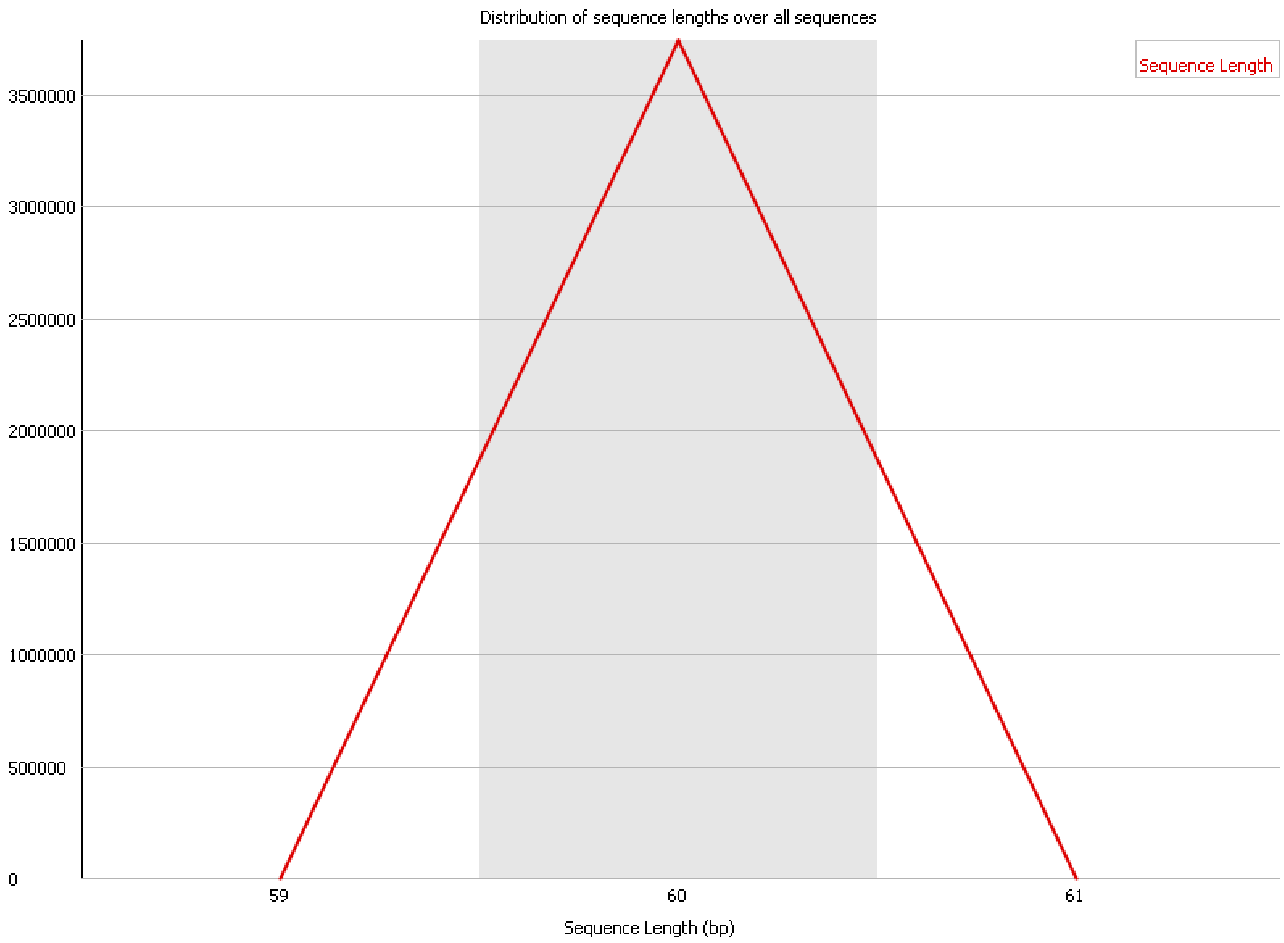

[Back to summary]

Sequence Duplication Levels

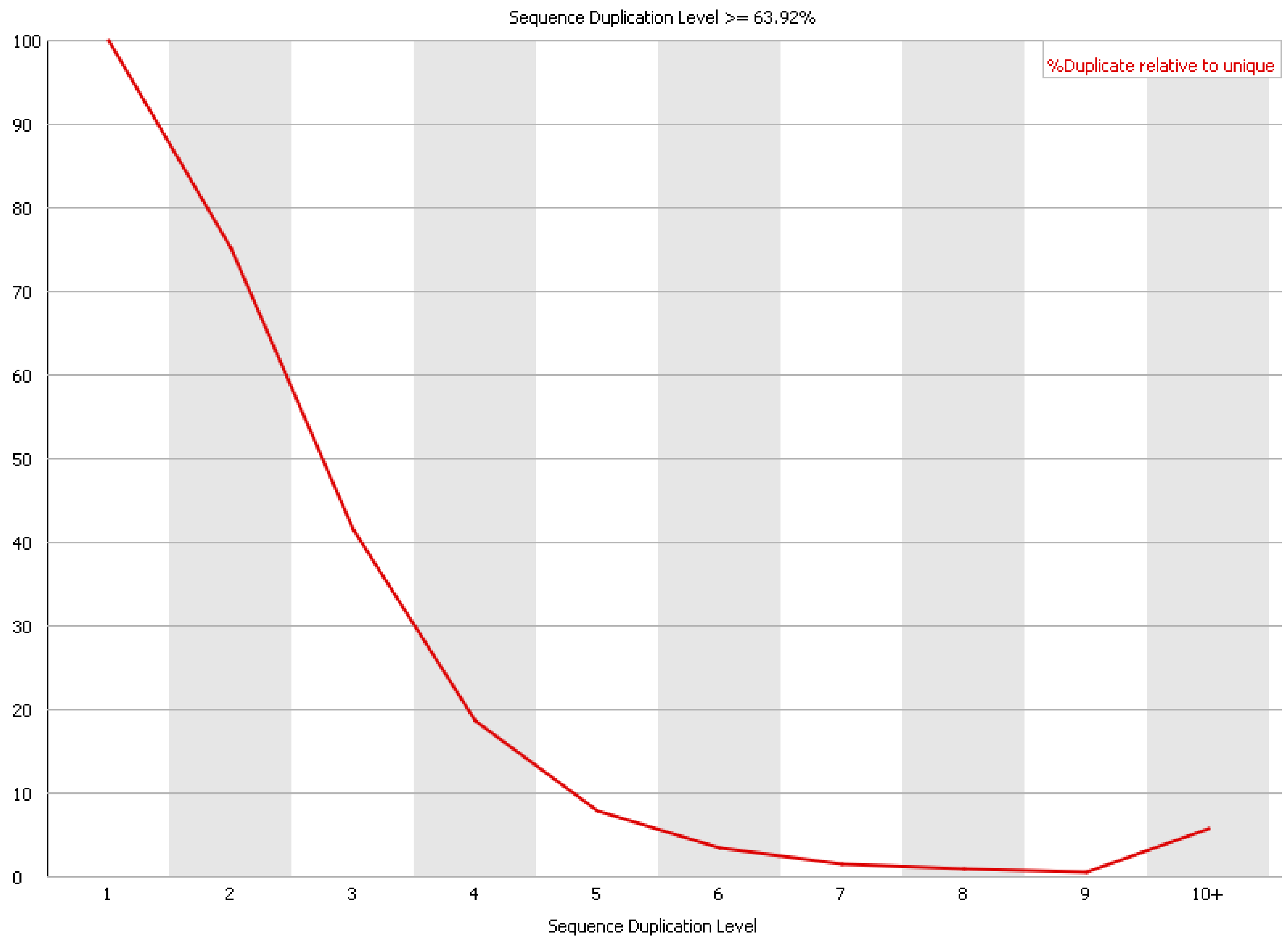

[Back to summary]

Overrepresented sequences

No overrepresented sequences

[Back to summary]

Kmer Content

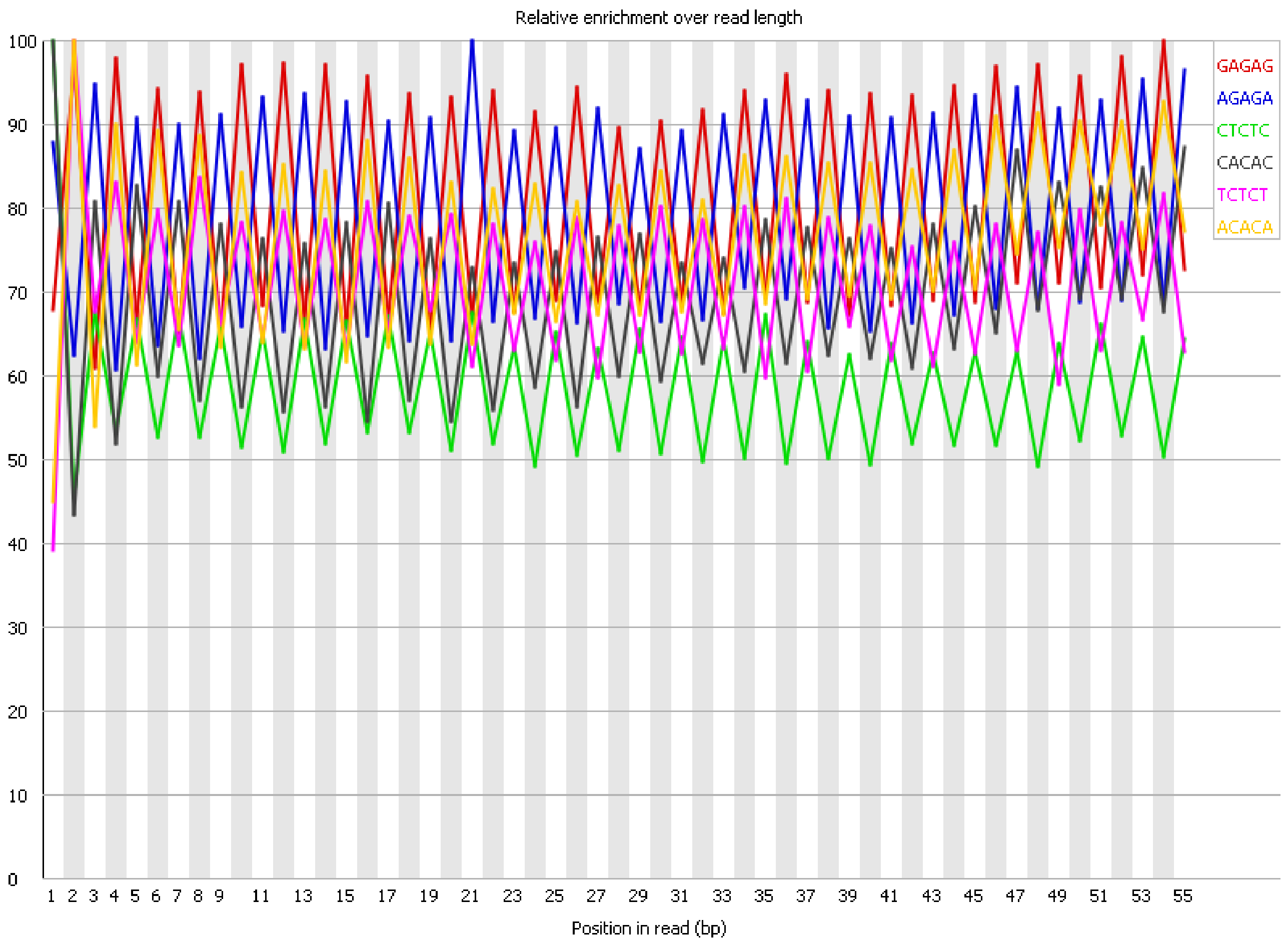

Sequence	Count	Obs/Exp Overall	Obs/Exp Max	Max Obs/Exp Position
GAGAG	1428690	8.5290985	10.483994	54
AGAGA	1546595	6.6827054	8.415197	21
CTCTC	816125	4.6517086	7.8920164	1
CACAC	888970	4.2003827	6.0200033	1
TCTCT	842050	4.124638	5.804397	2
ACACA	950610	3.5145457	4.581321	2
TTTTT	957515	3.4639993	5.1673374	42
GAGGG	375165	3.0944028	4.550071	1
GGGAG	368255	3.0374086	3.8401148	3
GGAGA	503550	3.0061297	3.587148	1
CTCTG	418740	2.5802083	5.3766656	22

Back to summary

Produced by FastQC (version 0.9.1)
