# Supplementary material for: Dynamic Changes in Ezh2 Gene Occupancy Underlie Its Involvement in Neural Stem Cell Self-Renewal and Differentiation towards Oligodendrocytes
Source: PLoS One. 2012 Jul 12;7(7):e40399. doi: 10.1371/journal.pone.0040399 (PMC3395718; doi:10.1371/journal.pone.0040399)
Supplement: Information S5 — ChIP-Seq NSCs FASTQC report. (PDF) [file pone.0040399.s014.pdf]

FastQC Report

Wed 13 Jul 2011

ChIP-Seq-NSCs.fastqsanger

Summary

- Basic Statistics
- Per base sequence quality
- Per sequence quality scores
- Per base sequence content
- Per base GC content
- Per sequence GC content
- Per base N content
- Sequence Length Distribution
- Sequence Duplication Levels
- Overrepresented sequences
- Kmer Content

Basic Statistics

Measure	Value
Filename	ChIP-Seq-NSCs.fastqsanger
File type	Conventional base calls
Encoding	Sanger / Illumina 1.9
Total Sequences	5548680
Sequence length	60
%GC	43

Back to summary

Per base sequence quality

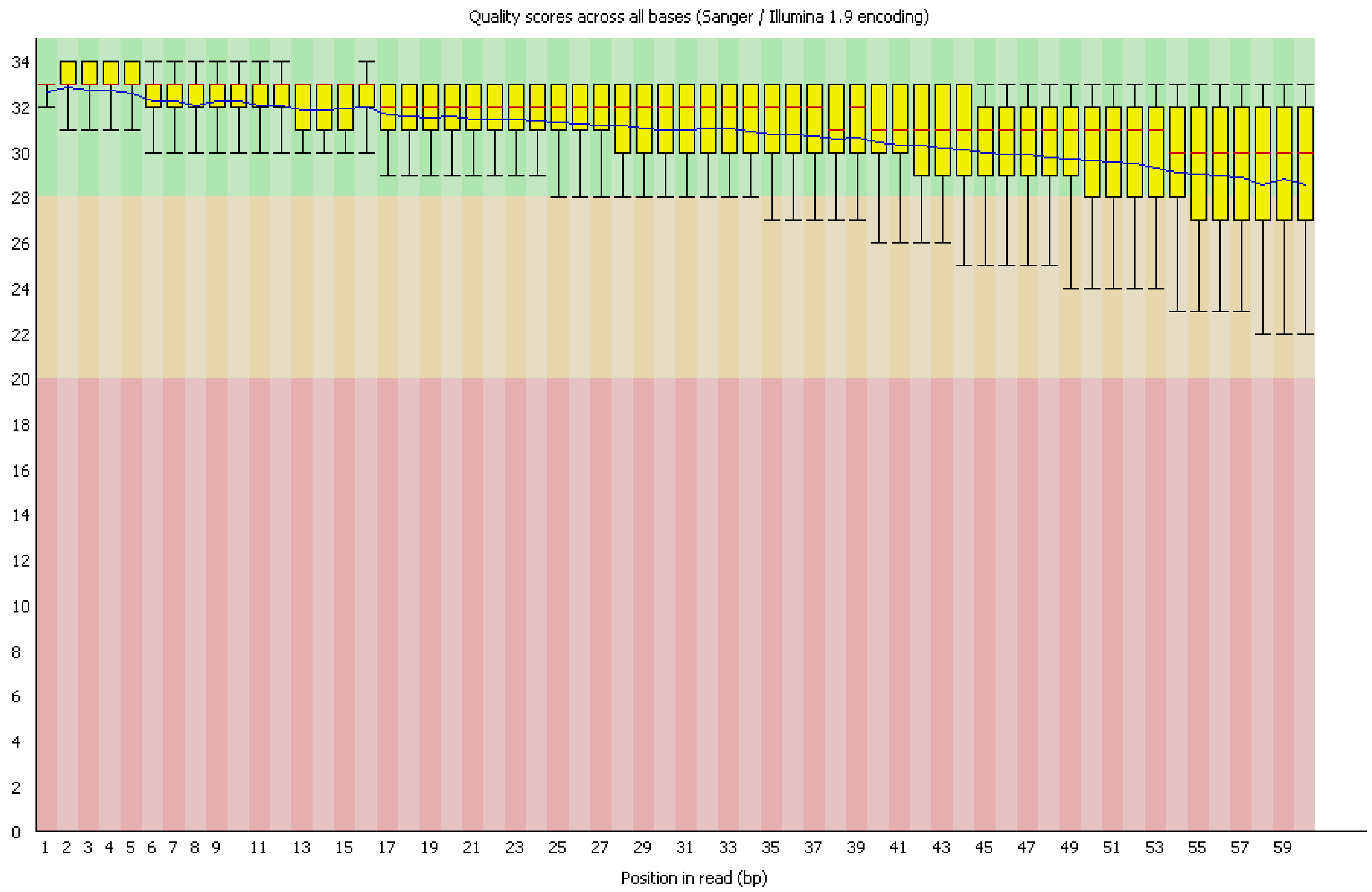

Back to summary

Per sequence quality scores

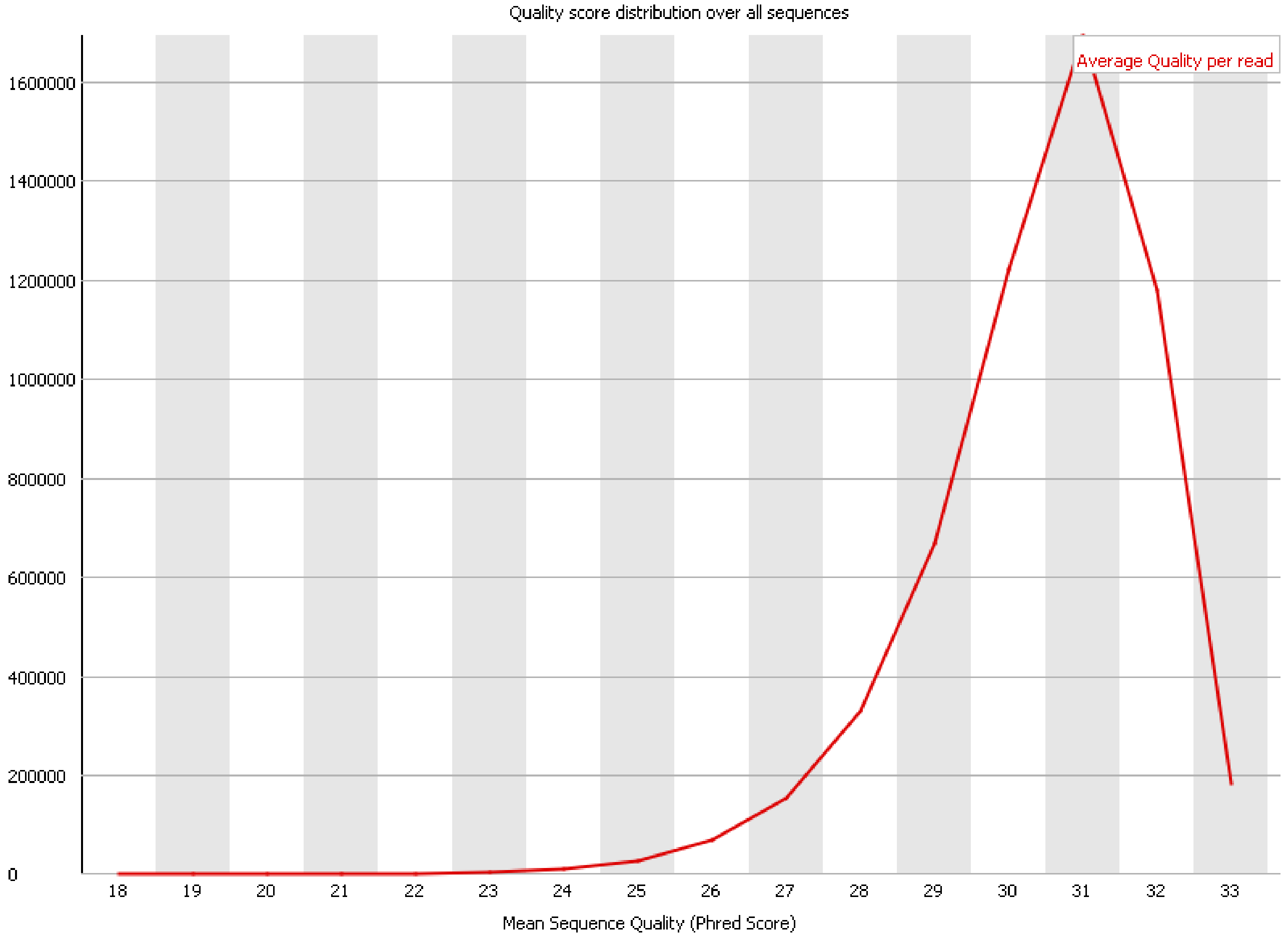

[Back to summary]

Per base sequence content

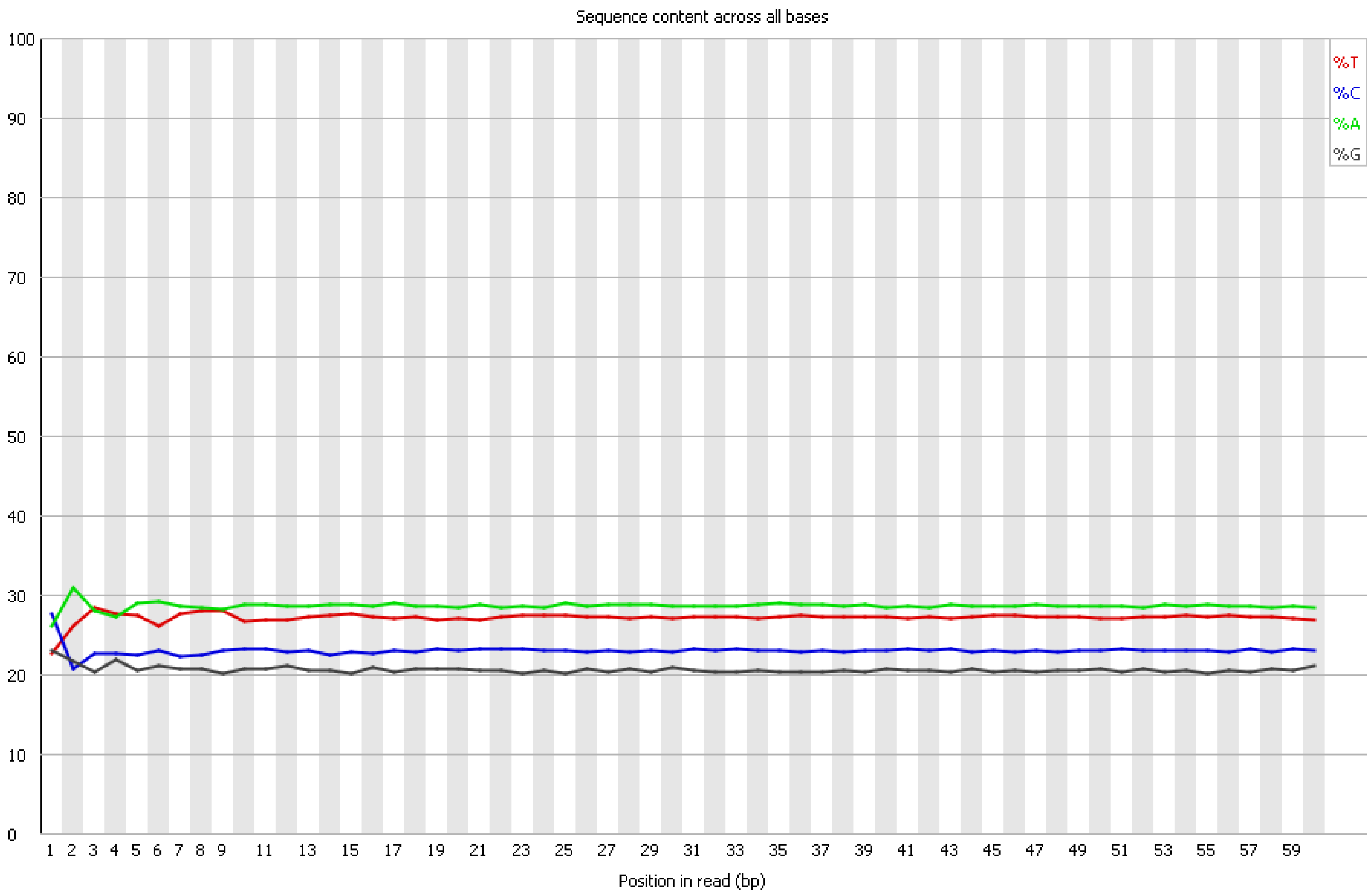

Back to summary

Per base GC content

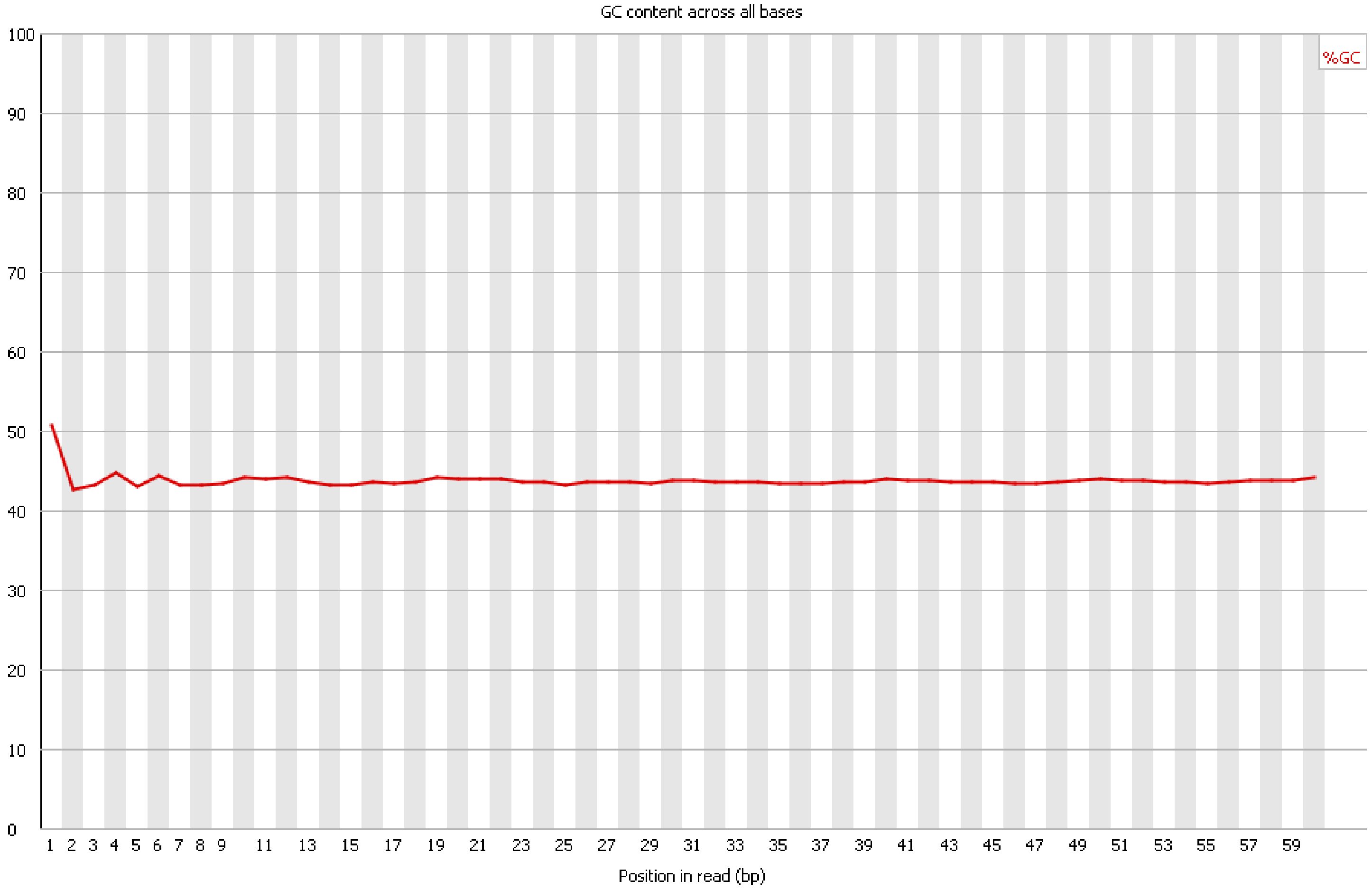

[Back to summary]

Per sequence GC content

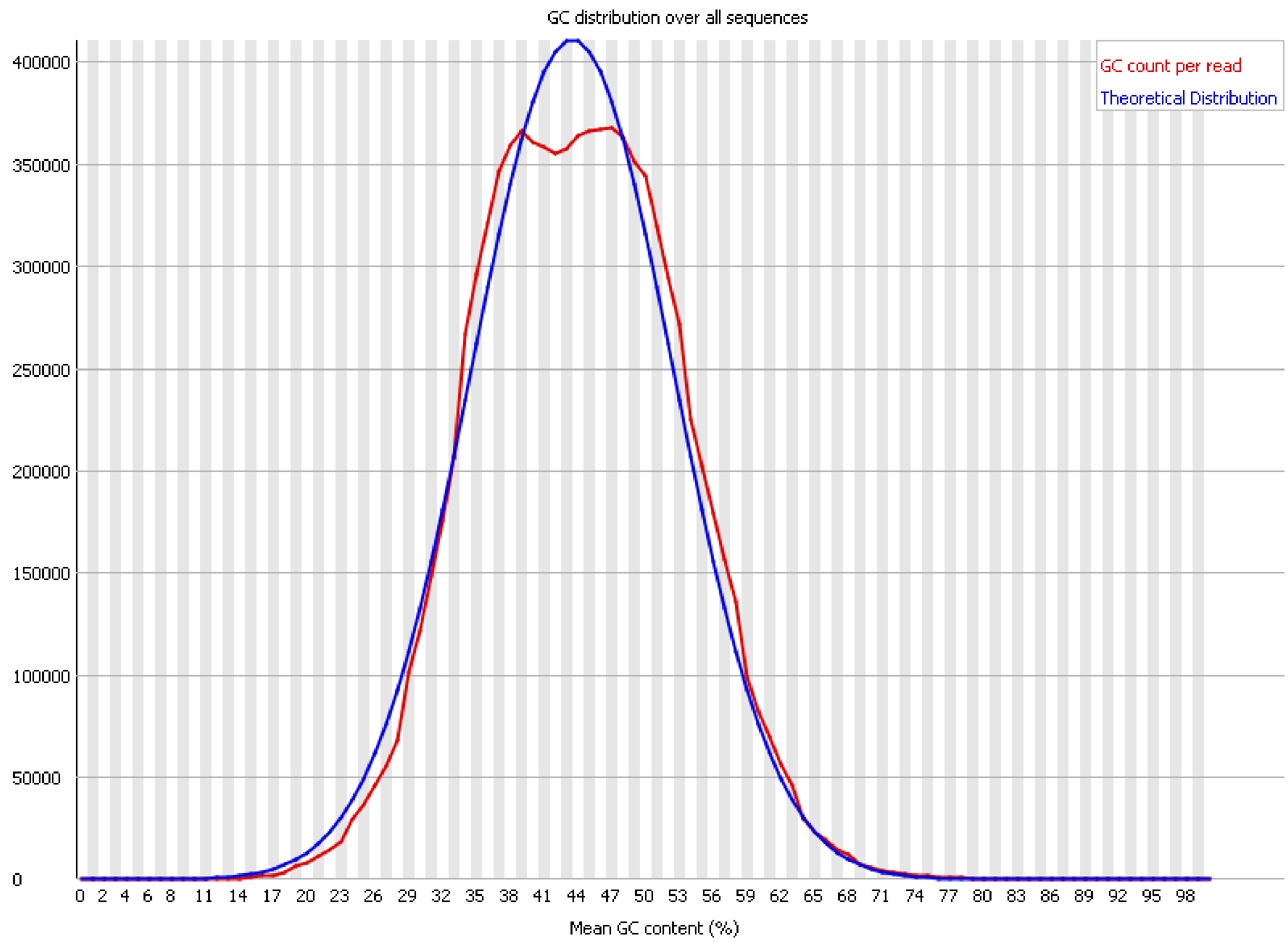

[Back to summary]

Per base N content

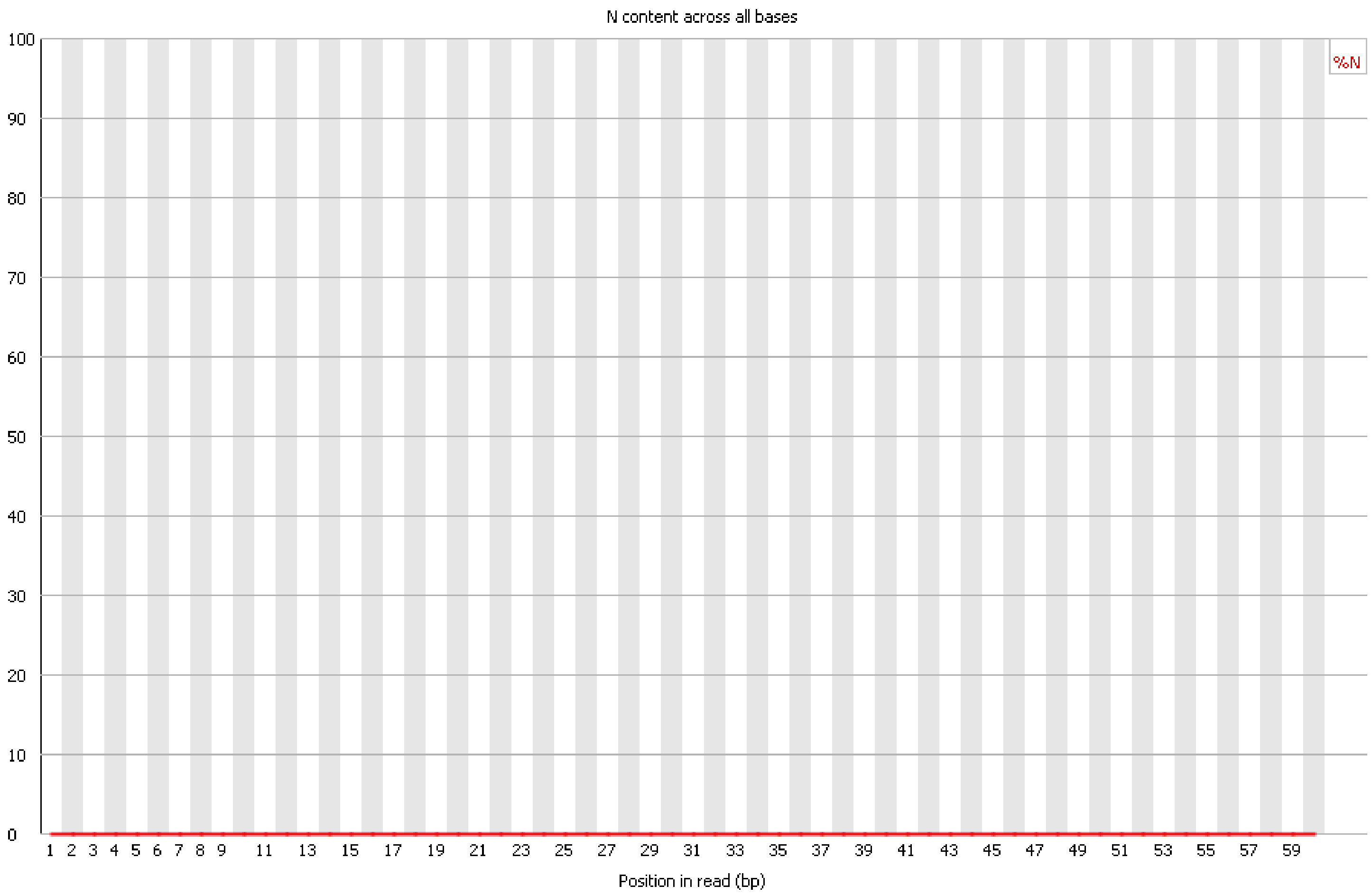

[Back to summary]

Sequence Length Distribution

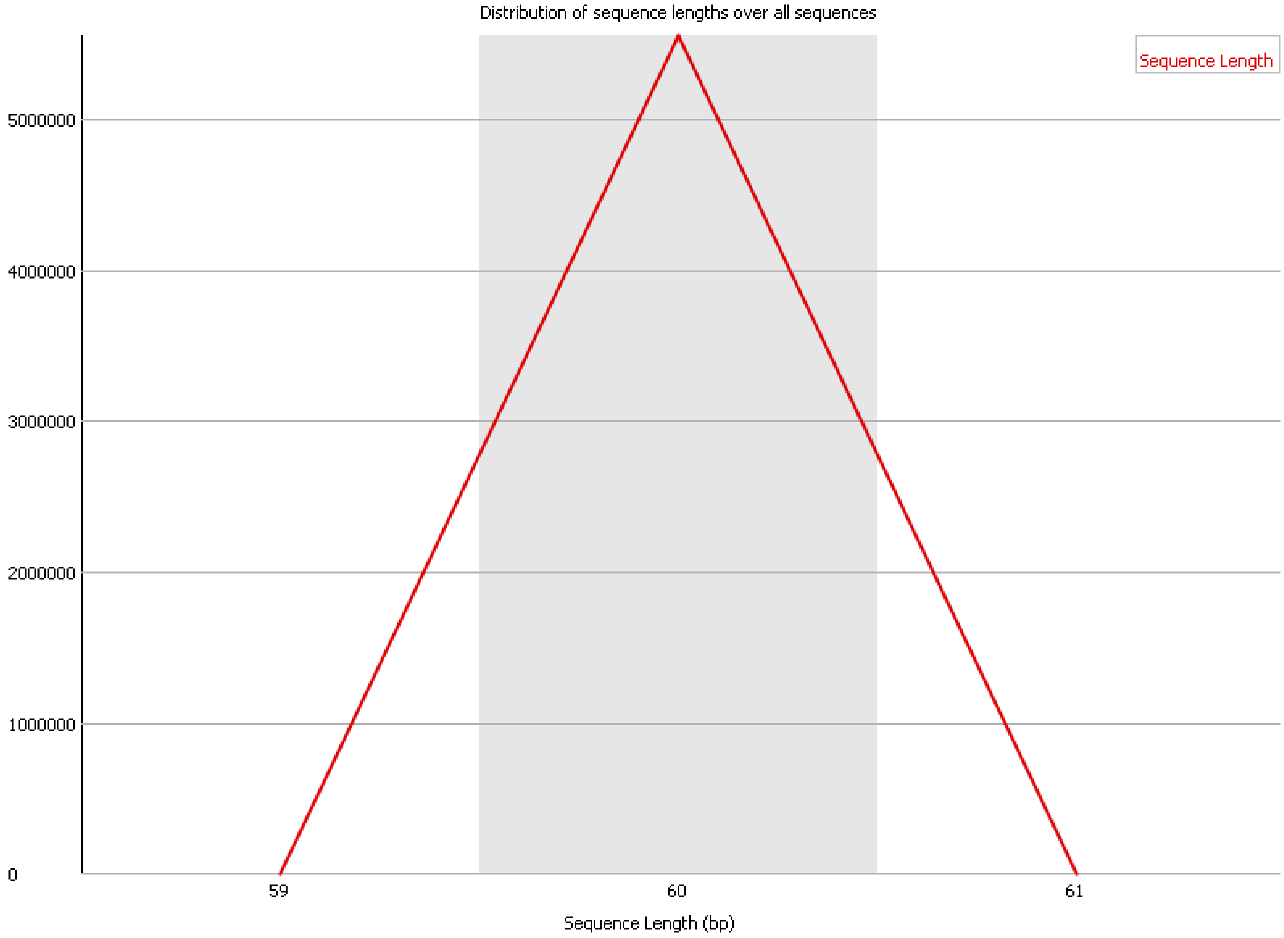

Back to summary

Sequence Duplication Levels

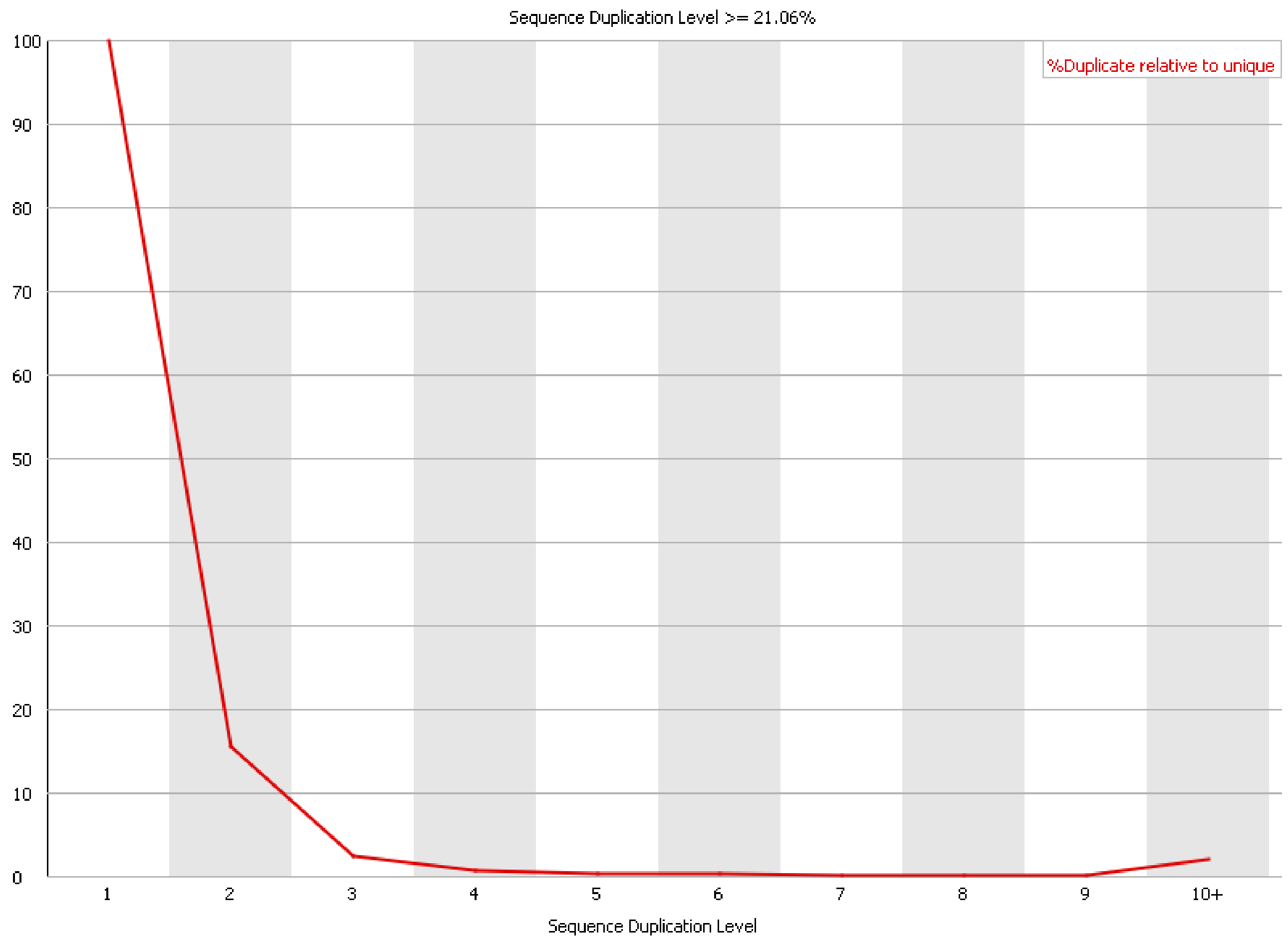

[Back to summary]

Overrepresented sequences

No overrepresented sequences

[Back to summary]

Kmer Content

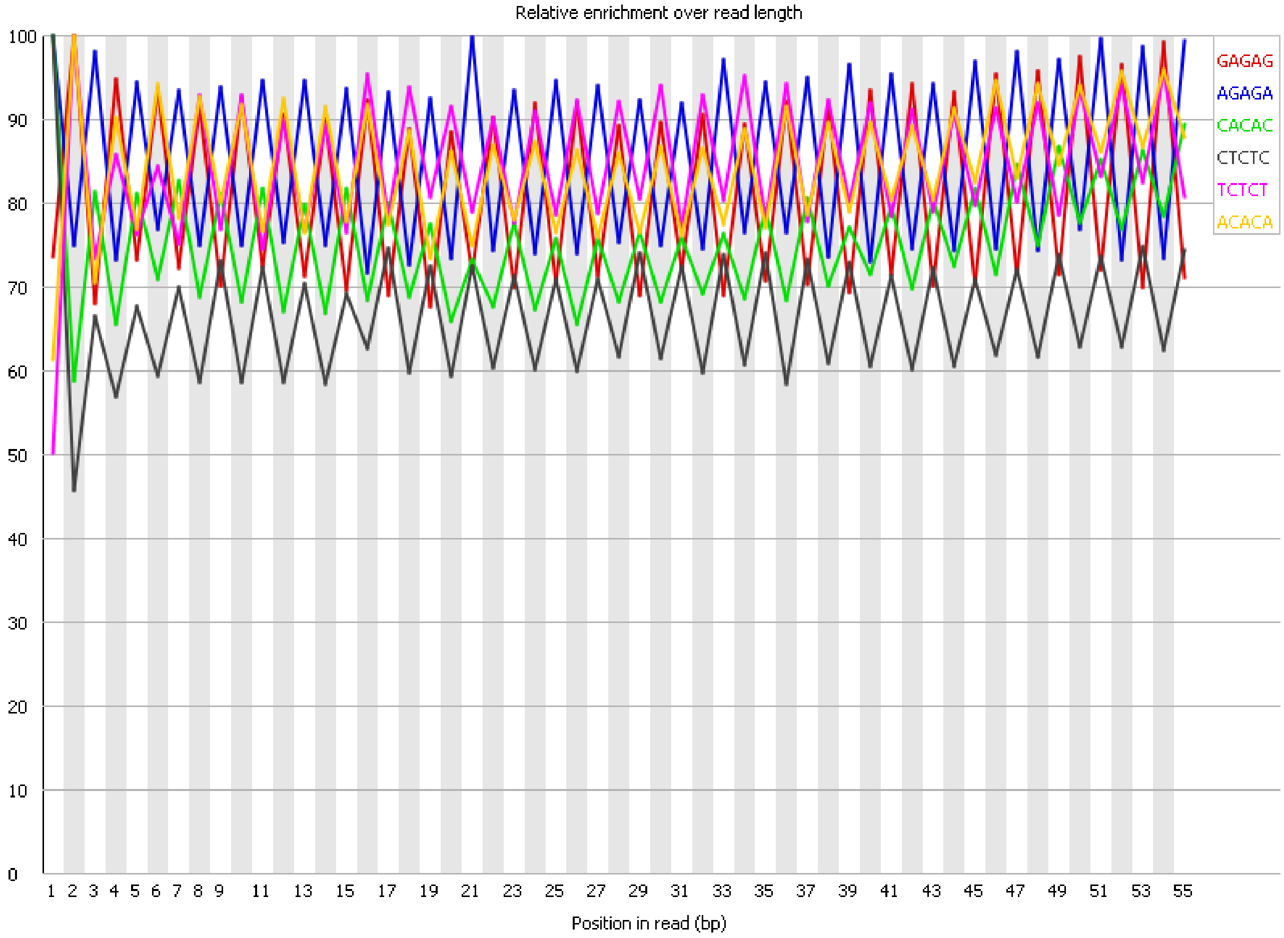

Sequence	Count	Obs/Exp Overall	Obs/Exp Max	Max Obs/Exp Position
GAGAG	1250695	5.548921	6.7970896	2
AGAGA	1445660	4.6301126	5.4237266	1
CACAC	1075935	3.4298215	4.5435925	1
CTCTC	969405	3.4188979	5.127703	1
TCTCT	1054250	3.1520834	3.7156014	2
ACACA	1204605	3.094964	3.6599352	2

Back to summary

Produced by FastQC (version 0.9.1)
