# Supplementary material for: Dynamic Changes in Ezh2 Gene Occupancy Underlie Its Involvement in Neural Stem Cell Self-Renewal and Differentiation towards Oligodendrocytes
Source: PLoS One. 2012 Jul 12;7(7):e40399. doi: 10.1371/journal.pone.0040399 (PMC3395718; doi:10.1371/journal.pone.0040399)
Supplement: Information S6 — ChIP-Seq pOLs FASTQC report. (PDF) [file pone.0040399.s015.pdf]

# FastQC Report

Wed 13 Jul 2011

ChIP-Seq-pOLs.fastqsanger

## Summary


- Basic Statistics
- Per base sequence quality
- Per sequence quality scores
- Per base sequence content
- Per base GC content
- Per sequence GC content
- Per base N content
- Sequence Length Distribution
- Sequence Duplication Levels
- Overrepresented sequences
- Kmer Content


## Basic Statistics

| Measure | Value |
|---|---|
| Filename | ChIP-Seq-pOLs.fastqsanger |
| File type | Conventional base calls |
| Encoding | Sanger / Illumina 1.9 |
| Total Sequences | 5095659 |
| Sequence length | 60 |
| %GC | 45 |

Back to summary

## Per base sequence quality

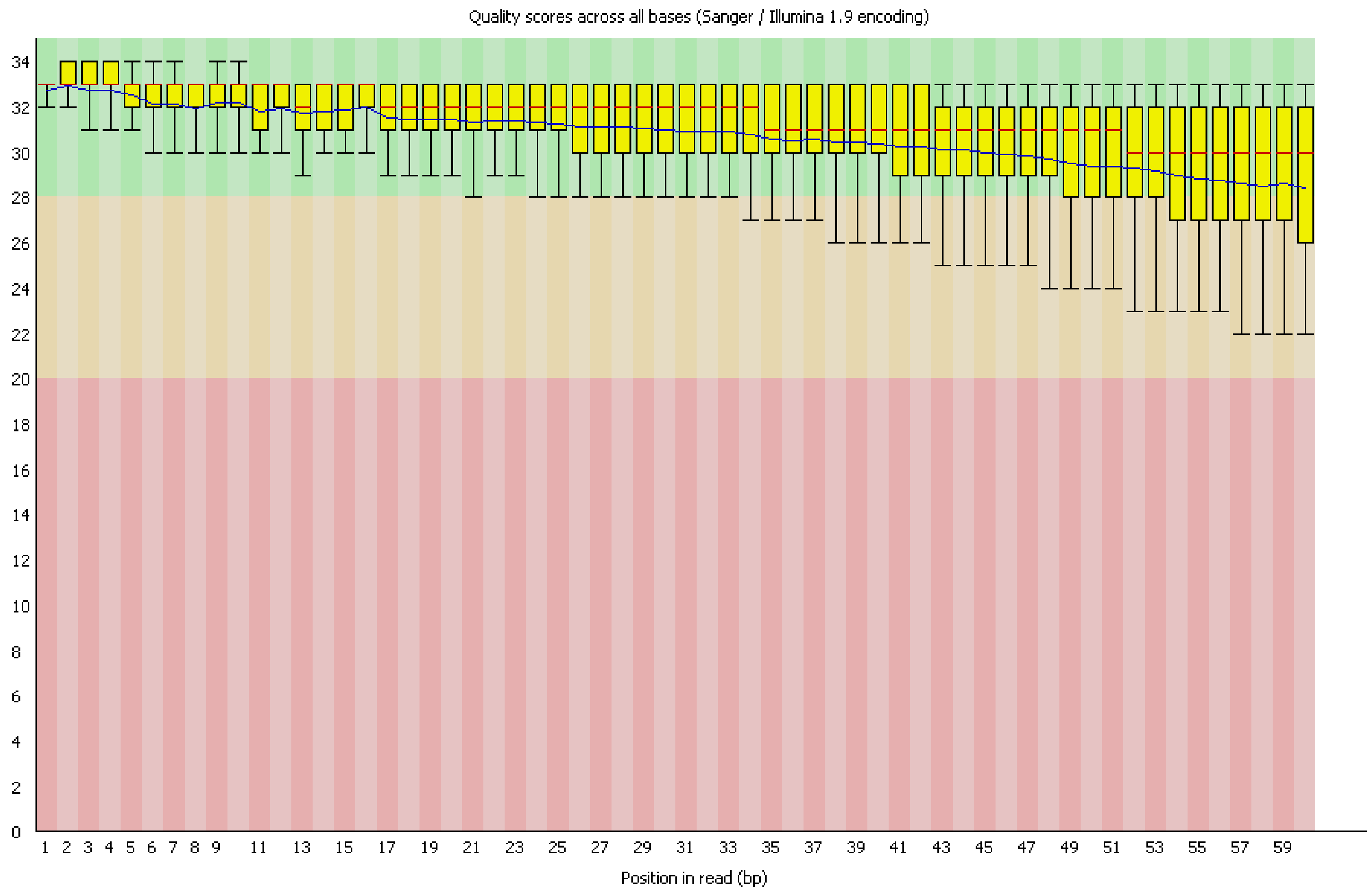


Back to summary

## Per sequence quality scores

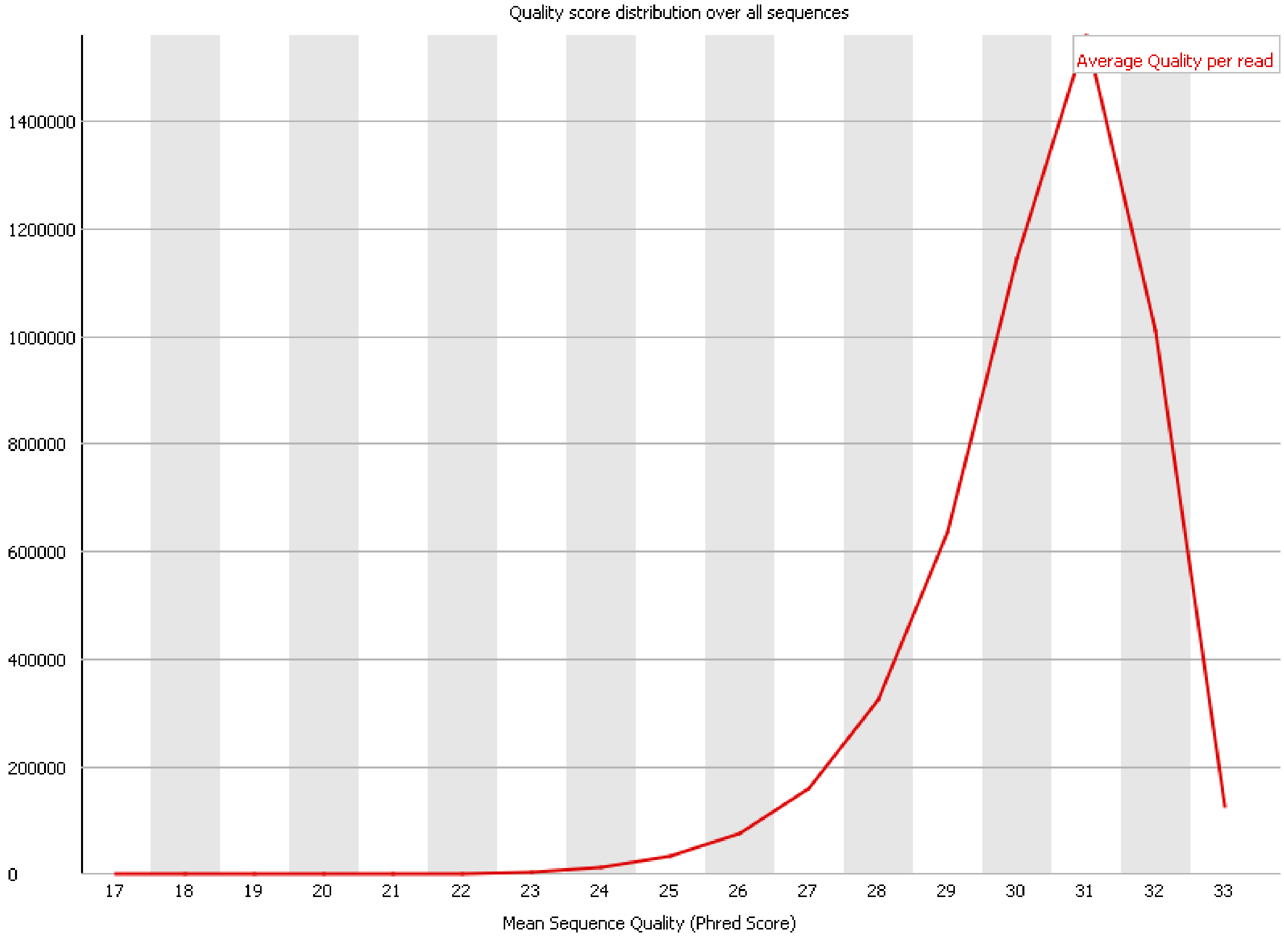


[Back to summary]

## Per base sequence content

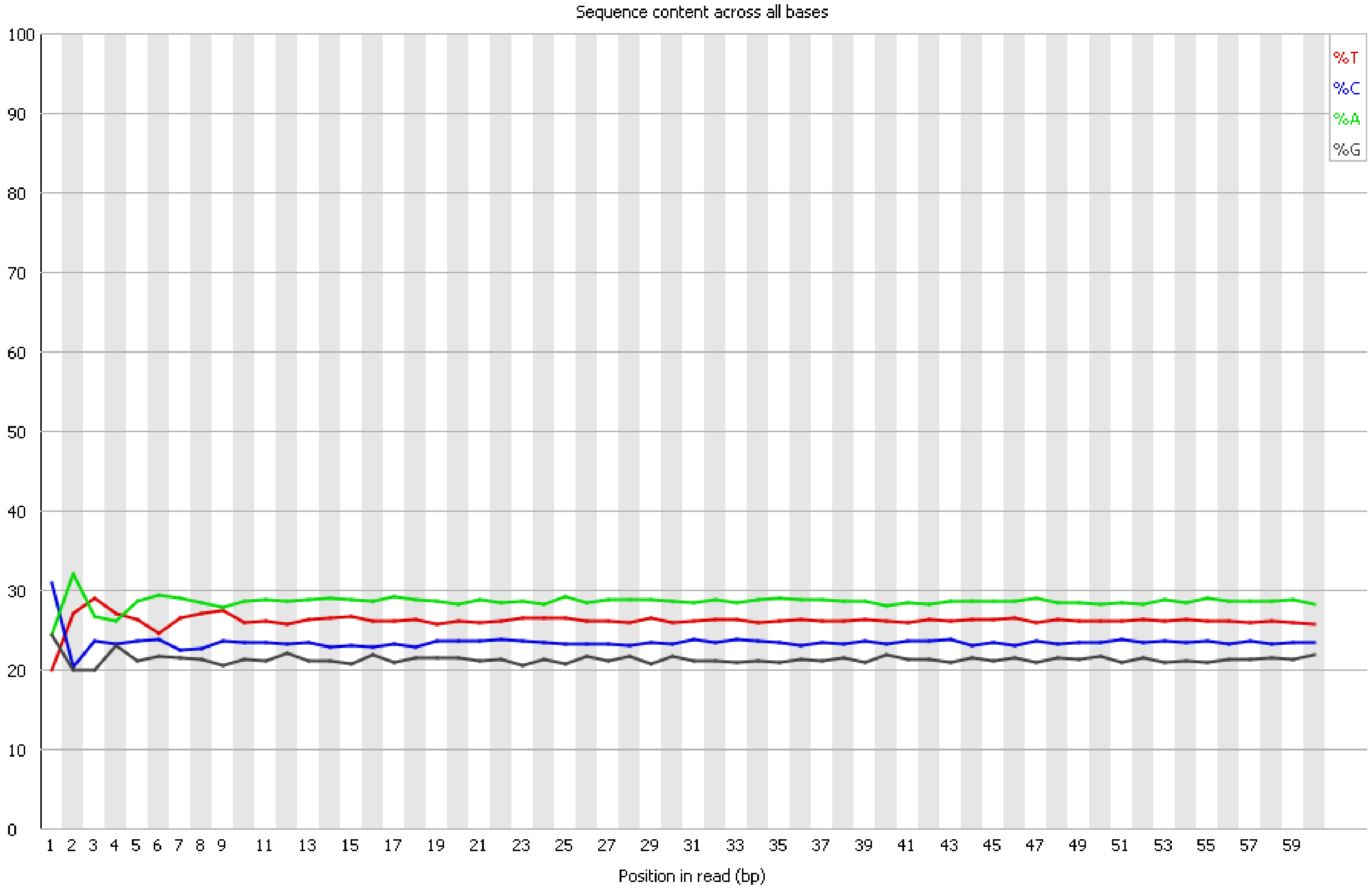


Back to summary

## Per base GC content

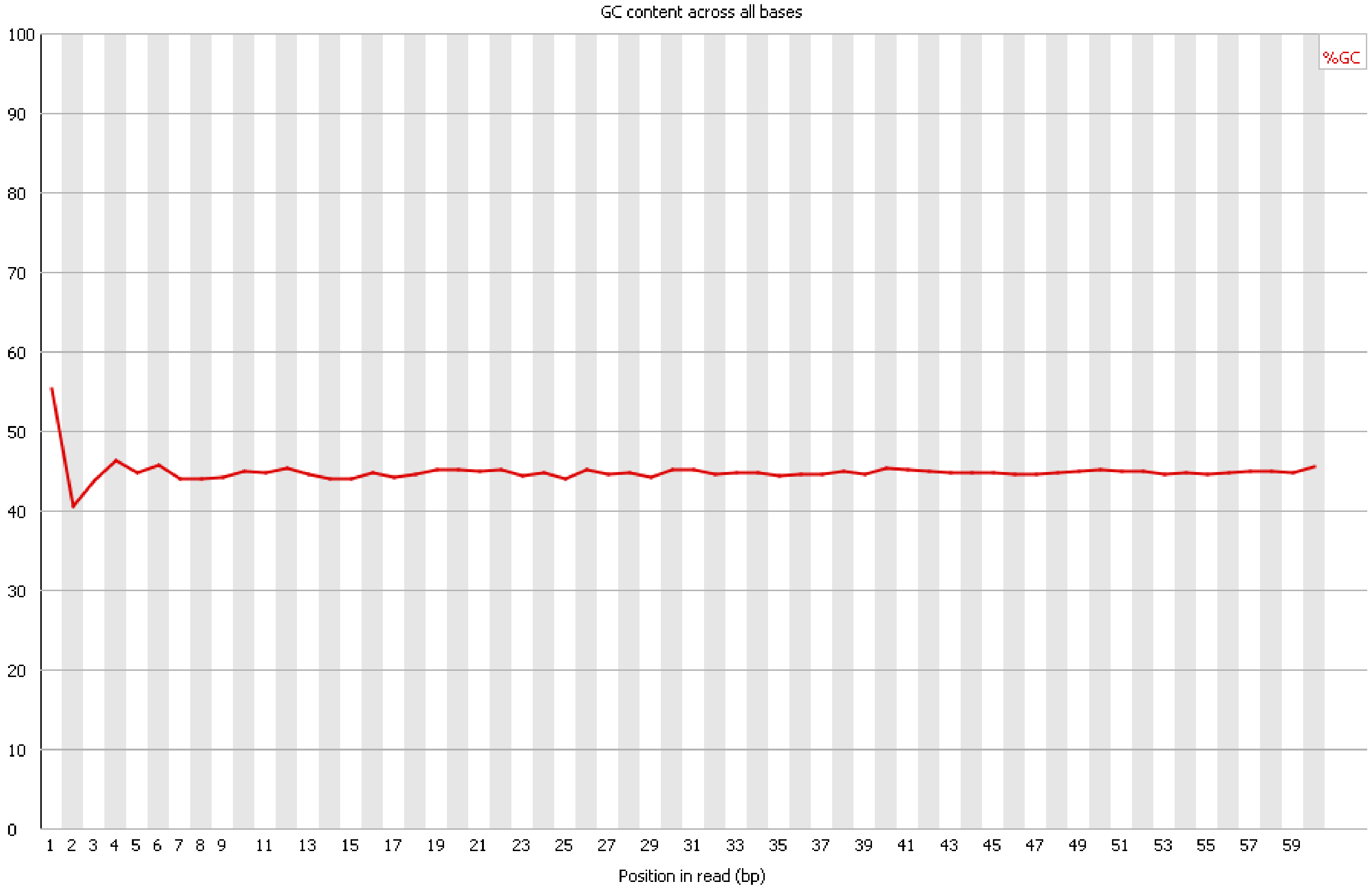


[Back to summary](#)

## Per sequence GC content

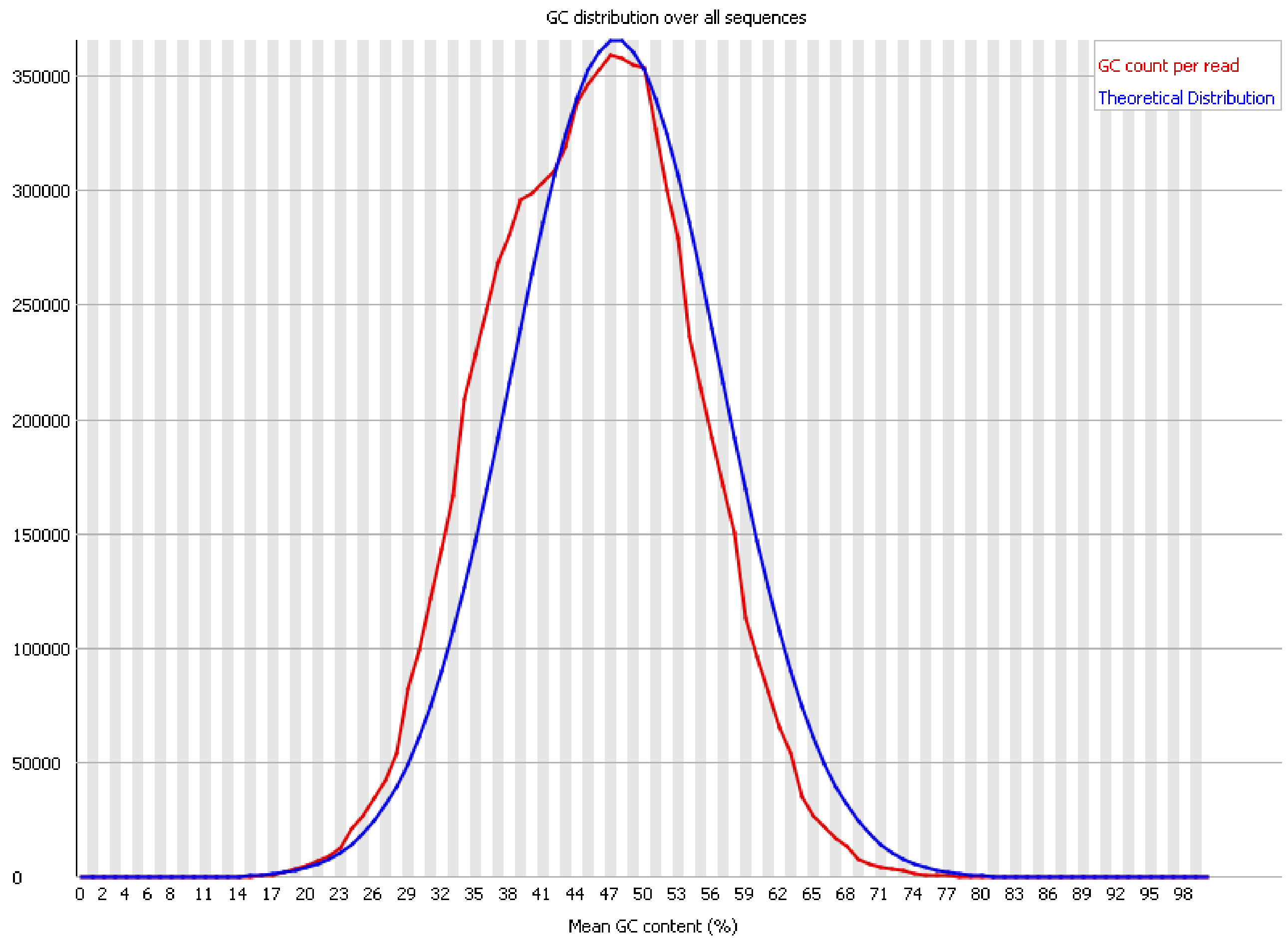


Back to summary

## Per base N content

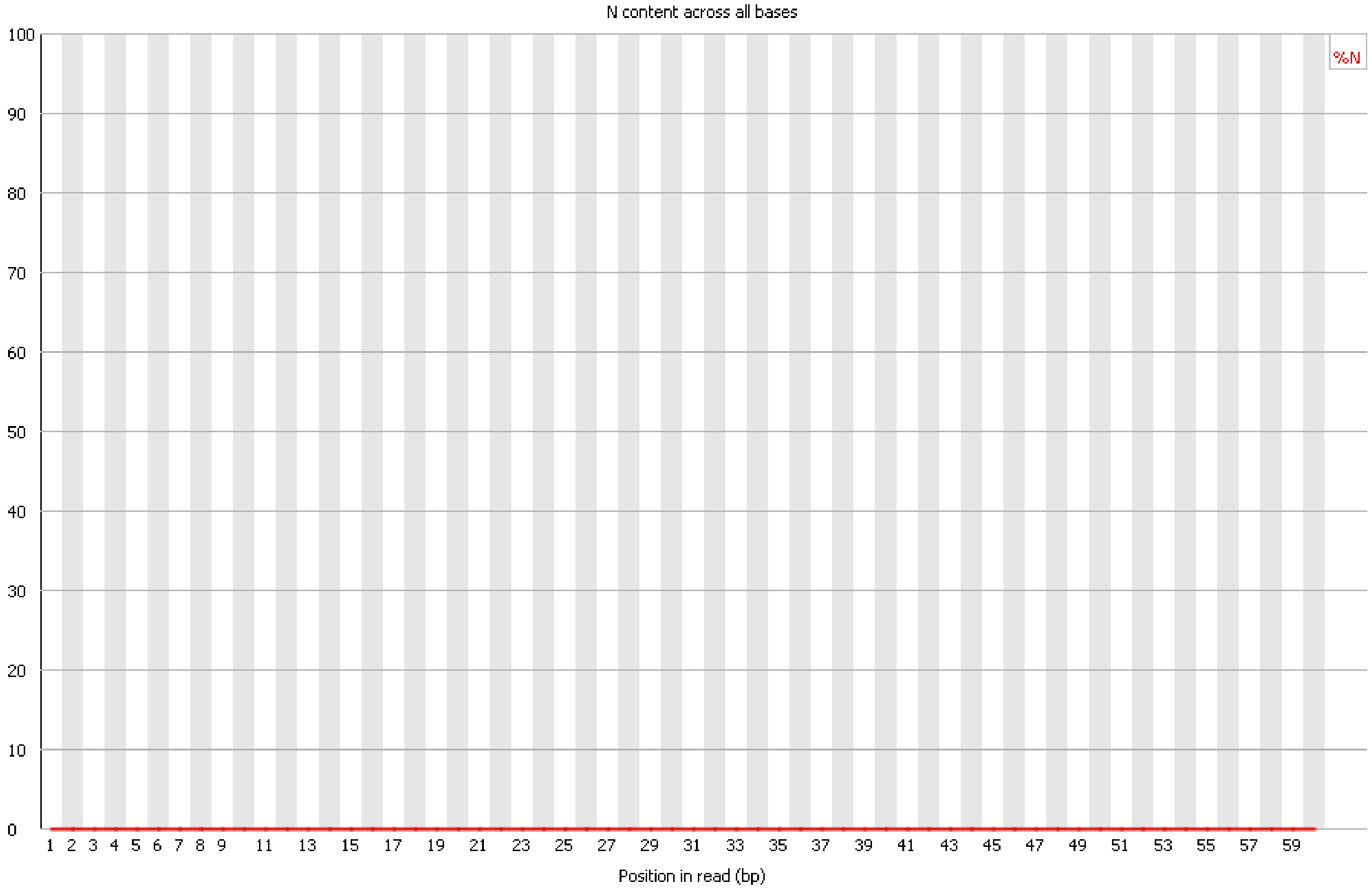


[Back to summary]

## Sequence Length Distribution

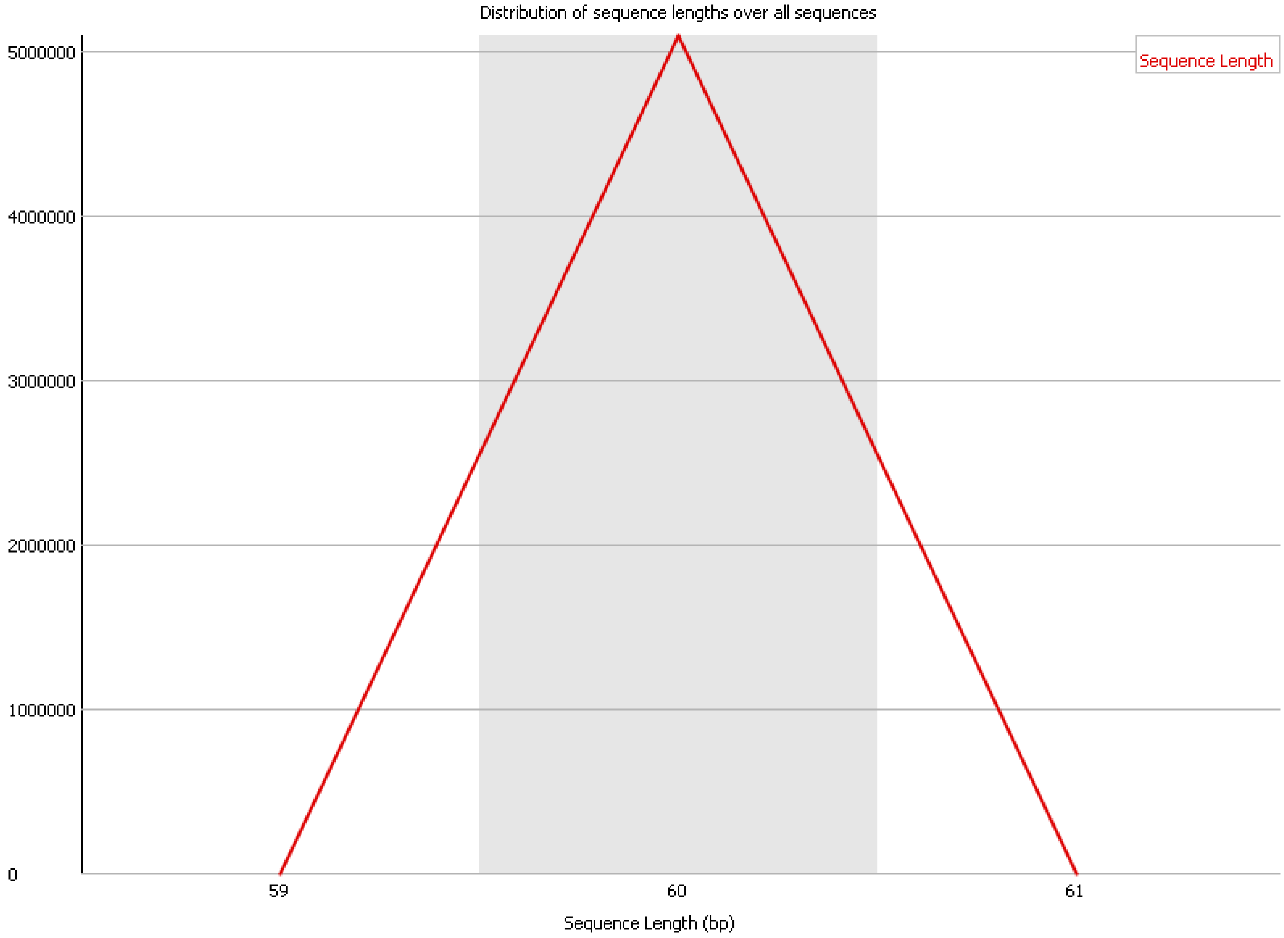


[Back to summary]

## Sequence Duplication Levels

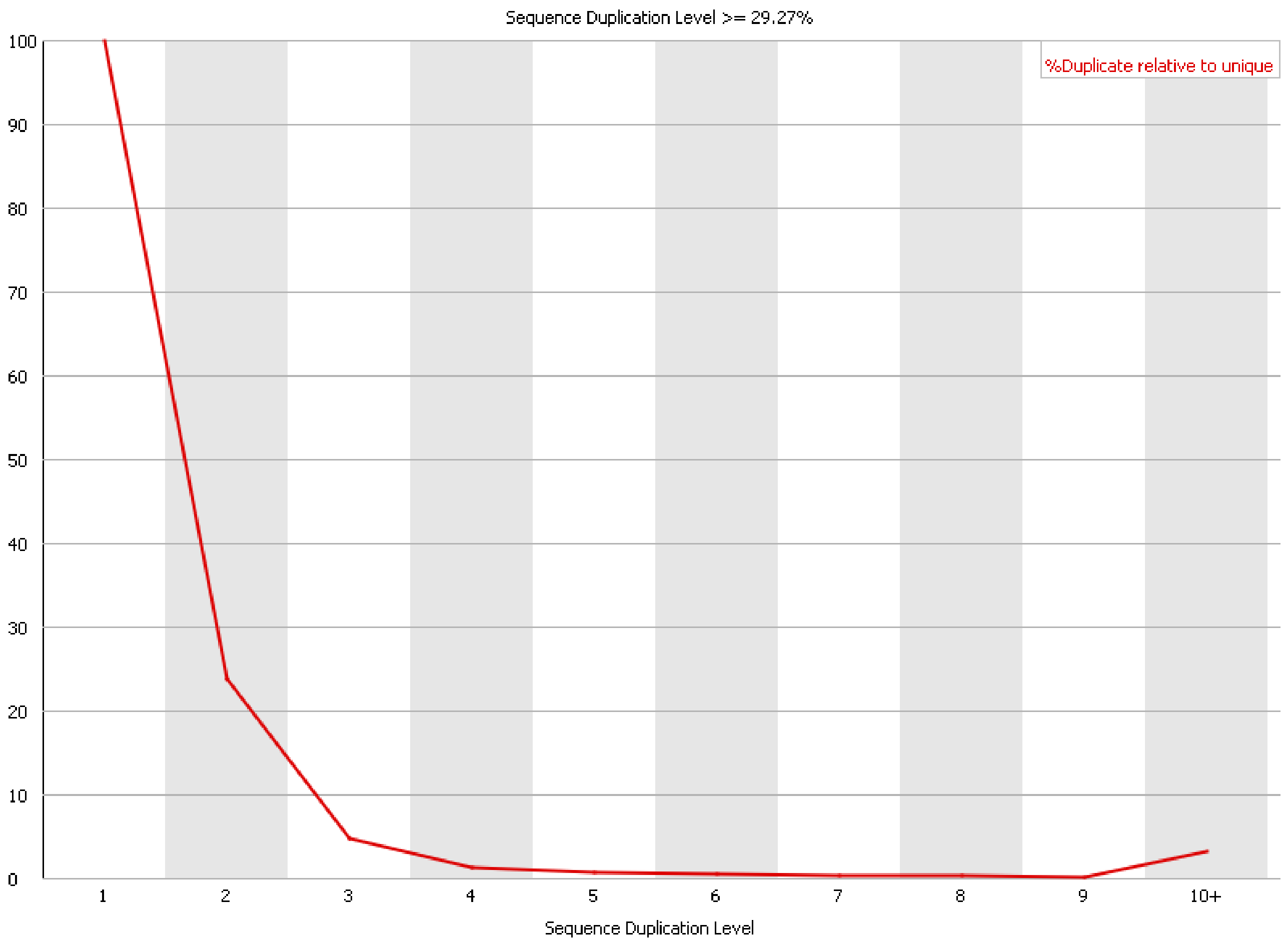


Back to summary

## Overrepresented sequences

No overrepresented sequences

Back to summary

## Kmer Content

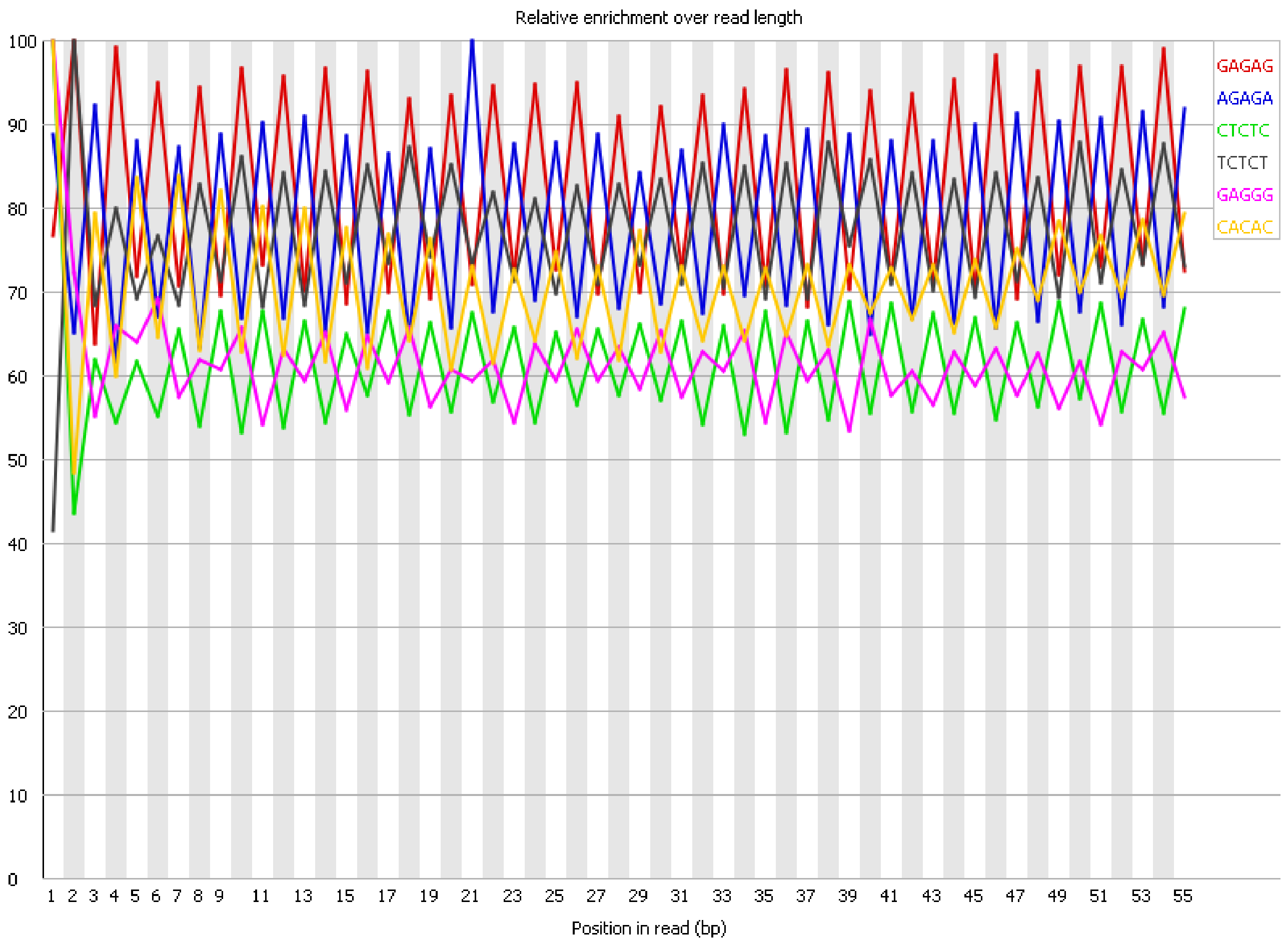


| Sequence | Count | Obs/Exp Overall | Obs/Exp Max | Max Obs/Exp Position |
|---|---|---|---|---|
| GAGAG | 1995480 | 8.804197 | 10.597129 | 2 |
| AGAGA | 2123230 | 6.993099 | 8.931539 | 21 |
| CTCTC | 1232670 | 4.842374 | 7.8667407 | 1 |
| TCTCT | 1243120 | 4.381121 | 5.6668015 | 2 |
| GAGGG | 612870 | 3.6222668 | 5.856107 | 1 |
| CACAC | 1097305 | 3.6217186 | 5.1173496 | 1 |
| GGAGA | 809570 | 3.5718794 | 4.114365 | 4 |
| AGAGG | 794160 | 3.5038893 | 3.9942465 | 27 |
| GGGAG | 574860 | 3.397615 | 4.344539 | 3 |
| ACACA | 1229790 | 3.337851 | 4.182797 | 2 |
| GGAGG | 556070 | 3.2865598 | 3.8211792 | 1 |
| AGGAG | 725135 | 3.1993463 | 4.121645 | 2 |
| GAGGA | 723495 | 3.1921103 | 4.417695 | 1 |
| CTCTG | 633585 | 2.7417934 | 6.119199 | 22 |
| TGTTT | 647720 | 2.255997 | 5.7210584 | 11 |
| CTGGT | 460675 | 2.196053 | 5.1323066 | 1 |
| TCTGG | 405640 | 1.9336994 | 5.327636 | 23 |
| GTTTG | 361110 | 1.5443594 | 5.646421 | 12 |
| TGATG | 391375 | 1.5342278 | 5.0311503 | 15 |

Back to summary

Produced by FastQC (version 0.9.1)
